# Small rodents, huge impact: A scoping review of European *Apodemus* species in the ecology of directly transmitted, environmentally mediated, and interface-associated zoonotic pathogens

**DOI:** 10.1016/j.onehlt.2026.101532

**Published:** 2026-07-28

**Authors:** Daniele Fabbri, Laura Grassi, Luca Dorigo, Stefania Leopardi, Valentina Tagliapietra, Paola Beraldo

**Affiliations:** a Department of Agricultural, Food, Environmental and Animal Sciences, University of Udine, Via Sondrio 2/A, Udine, UD, Italy; b NBFC, National Biodiversity Future Center, Piazza Marina 61, Palermo, PM, Italy; c Istituto Zooprofilattico Sperimentale delle Venezie, Via della Roggia, 100, Campoformido, UD, Italy; d Museo Friulano di Storia Naturale, Via C.G. Sabbadini, Udine, UD, Italy; e Istituto Zooprofilattico Sperimentale delle Venezie, Viale dell'Università, 10, Legnaro, PD, Italy; f Research and Innovation Center, Fondazione Edmund Mach, Via E. Mach 1, 38098 San Michele all'Adige, TN, Italy

**Keywords:** *Apodemus*, Zoonotic pathogens, One health, Reservoir competence, Environmental transmission, Ecological determinants, Scoping review

## Abstract

European *Apodemus* rodents host zoonotic pathogens across wildlife, agricultural and peri-urban interfaces, but the evidence remains fragmented. We conducted a PRISMA-guided scoping review of English-language, peer-reviewed primary studies published during 2000–2025 reporting positive *Apodemus*-specific evidence for zoonotic viral or bacterial pathogens in Europe. Europe PMC, PubMed, Web of Science and CABI Digital Library were searched, with supplementary citation searching. Of 4051 pathogen-specific records screened, 166 eligible pathogen-specific article records were retained, representing 141 unique publications and 241 pathogen-study records. We charted pathogen, host, geography, diagnostic method, evidence type and ecological determinants. Evidence was strongest for Hantaviridae and *Leptospira* spp.; moderate or context-dependent for Arenaviridae, Poxviridae, *Francisella tularensis*, *Yersinia* spp., *Campylobacter* spp., *Escherichia coli* and *Staphylococcus aureus*; and sparse or principally detection-oriented for Astroviridae, Coronaviridae, Paramyxoviridae, *Clostridioides difficile* and *Mycobacterium* spp. Reported determinants included host traits and abundance, seasonality, resource pulses, habitat and land use, rainfall, temperature, humidity, flooding and synanthropic exposure. Substantial heterogeneity in study design, assays, host identification, denominators and ecological covariates precluded pooled prevalence or effect synthesis. Detection or seropositivity was therefore not interpreted as proof of reservoir competence. European *Apodemus* species are best regarded as potential reservoirs in selected systems and, more broadly, as ecological interface hosts connecting wildlife, environmental, livestock, peri-urban and human-exposure pathways. One Health surveillance should integrate host monitoring and identification with pathogen diagnostics, environmental indicators, veterinary reporting, public-health data and human-exposure surveillance at shared spatial and temporal scales.

## Highlights

• PRISMA-guided scoping review of 168 European studies of zoonotic pathogens in Apodemus spp. (2000–2025).
• Evidence was mapped by pathogen, host, geography, diagnostics, prevalence, and ecological determinants.
• Apodemus spp. were often associated with hantaviruses, arenaviruses, cowpox virus, Leptospira, Francisella and Yersinia.
• Other viruses and bacteria, including coronaviruses and antimicrobial-resistant E. coli, showed broader diversity.
• Host traits, density, seasonality, mast, habitat, and climate were recurrent ecological determinants.
• Detection alone did not establish reservoir competence, underscoring integrated One Health surveillance.

## 1 Introduction

Rodents comprise approximately 40% of known mammal species [1] and 16% of global wild mammal biomass [2]. They support ecosystem functioning but can also damage crops and host zoonotic pathogens [3], [4], [5], [6]. Population fluctuations, habitat change and host traits can influence pathogen circulation, although these relationships are species- and context-dependent [4], [7], [8], [9], [10], [11], [12], [13], [14], [15], [16], [17], [18], [19], [20]. Across European rodent systems, host–pathogen associations and reservoir competence vary substantially among species and pathogens [21], [22], [23].

*Apodemus* species are widespread and locally abundant in natural, agricultural, peri-domestic and urban-interface habitats [24], [25]. Their taxonomy remains debated: some classifications suggest broader or potentially paraphyletic groupings [26], whereas others use *Apodemus* sensu lato to include *Sylvaemus* and *Apodemus* sensu stricto lineages [27], [28].

We use this pragmatic definition and include *A. alpicola*, *A. witherbyi* (= *A. arianus*), *A. flavicollis*, *A. sylvaticus*, *A. uralensis*, *A. epimelas*, *A. mystacinus* and *A. agrarius* [29]. Overlapping distributions and difficult morphological identification can, however, limit species-level epidemiological inference.

The breadth of habitats occupied by *Apodemus*, including human-modified environments, makes these rodents useful sentinels of pathogen circulation at wildlife–human interfaces [30], [31], [32]. Climate and land-use change can modify *Apodemus* abundance, distribution and contact with people, livestock, domestic animals and contaminated substrates, although effects are pathogen-, species- and context-dependent [14], [33], [34], [35], [36], [37]. In Mediterranean systems, rainfall has been linked to *A. sylvaticus* reproduction and abundance [38], [39], while warming may facilitate the northwestern expansion of *A. agrarius* and associated hantaviruses and *Leptospira* spp. [40], [41]. Flooding or altered mast dynamics may also modify exposure and transmission opportunities in *Apodemus*-associated systems [43], [44], [45], [46].

These processes require an operational One Health framework linking standardised rodent trapping and *Apodemus* identification with pathogen diagnostics and environmental, veterinary, public-health and human-exposure data in shared spatial and temporal units. This positive-evidence scoping review maps zoonotic viral and bacterial pathogens reported in European *Apodemus* populations, distinguishes detection, exposure, carriage and reservoir-consistent evidence, and charts reported ecological determinants. Because methods and reporting are heterogeneous, the review provides a qualitative evidence map rather than pooled prevalence estimates or meta-analysis.

## 2 Methods

### 2.1 Review design

This positive-evidence PRISMA-guided scoping review was conducted between February and December 2025 to map evidence on zoonotic viral and bacterial pathogens reported in European *Apodemus* populations. A scoping-review design was selected because the available literature differed substantially in pathogen group, diagnostic method, host attribution, sampling design, geographic resolution, and reporting of detection or prevalence outcomes. Reporting was aligned with PRISMA-ScR principles where applicable [47].

The review question followed a population–concept–context structure. The population was European *Apodemus* spp.; the concept was diagnostic, serological, molecular, culture-based, metagenomic, prevalence, or ecological evidence for zoonotic viral and bacterial pathogens; and the context was wild, free-ranging, synanthropic, or peri-domestic *Apodemus* populations sampled in Europe. The evidence-charting form was piloted on a subset of studies representing viral, bacterial, denominator-based, and detection-only records, then refined before full extraction.

### 2.2 Protocol and registration

No review protocol was registered. The review methods, eligibility criteria, evidence-charting fields, and evidence-classification approach were defined before the revised evidence-charting stage and are reported here to support transparency and reproducibility.

No formal critical appraisal or risk-of-bias scoring was conducted because the purpose was to map the extent, type, and interpretability of evidence rather than to rank studies for quantitative synthesis. Instead, evidence limitations were appraised descriptively according to diagnostic type, host-identification resolution, denominator availability, spatial resolution, and reservoir-inference strength.

### 2.3 Search strategy

Structured searches were conducted in Europe PMC, PubMed, Web of Science, and CABI Digital Library. Searches covered peer-reviewed articles published in English between 2000 and 2025 and combined host terms, pathogen-specific terms and geographic terms. Host terms included *Apodemus*, “wood mouse”, “yellow-necked mouse”, and “striped field mouse”. Geographic terms included “Europe” and individual European country names. Pathogen-specific search blocks were built for all viral and bacterial pathogens included in the review. The final formal database searches were completed in December 2025. Complete database-specific queries are provided in Supplementary Table S1.

Additional sensitivity searches used broader terms including zoonotic, zoonosis, pathogen, infection, detection, prevalence, seroprevalence and reservoir, together with selected country–pathogen combinations, to check retrieval coverage. These searches yielded no additional eligible records beyond the formal pathogen-specific database searches and were therefore not treated as a separate PRISMA retrieval stream. Ecological-determinant information was charted when reported in otherwise eligible pathogen studies; no separate systematic search for determinant studies was undertaken.

### 2.4 Eligibility criteria

Studies were included when they were peer-reviewed primary research articles published in English between 2000 and 2025; reported data from Europe, defined according to the UNSD M49 classification and excluding the Asian portion of the Russian Federation; sampled wild, free-ranging, synanthropic, or peri-domestic *Apodemus* spp.; identified hosts at least at genus level; and reported at least one positive diagnostic, serological, molecular, culture-based, metagenomic, or otherwise interpretable pathogen record attributable to *Apodemus* spp. for a zoonotic viral or bacterial pathogen.

Studies were excluded when they were reviews, editorials, book chapters, conference abstracts, theses, grey literature, unavailable full texts, non-English full texts, outside the geographic scope, lacked original animal- or sample-level data, did not include *Apodemus* spp., or did not allow pathogen results to be attributed to *Apodemus* at genus or species level. Laboratory studies were excluded unless they included original data from naturally infected European *Apodemus* populations. Studies reporting pathogens only in non-*Apodemus* hosts, studies without *Apodemus* data, studies with results pooled across hosts without *Apodemus*-specific attribution, and studies in which *Apodemus* spp. were reported only as negative were excluded because the review was designed to map documented positive *Apodemus*-associated pathogen evidence. The objective was to map documented *Apodemus*-associated pathogen detection, exposure, carriage, or prevalence records, not to estimate pathogen absence, zero prevalence, pooled prevalence, or comparative host competence. Detailed inclusion and exclusion criteria are provided in Supplementary Table S2.

### 2.5 Screening and study selection

Records were imported into Rayyan for duplicate detection and screening [48]. After duplicate removal, titles and abstracts were screened against the eligibility criteria, followed by full-text assessment of potentially eligible articles. Reference lists of included articles were manually screened, and backward citation searching was performed in Zotero. Records identified through citation searching were assessed using the same criteria and recorded separately in the PRISMA flow diagram.

Title-and-abstract screening and full-text eligibility assessment were conducted by one reviewer, DF. Records of uncertain eligibility during title-and-abstract screening were retained for full-text assessment, and final decisions were made by applying the predefined inclusion and exclusion criteria. The review scope and interpretation were discussed among the author team, but no independent duplicate screening was undertaken.

### 2.6 Data extraction and unit of analysis

Data were extracted by one reviewer into a structured evidence-charting table; no independent duplicate data extraction was undertaken. Extracted variables included author and year, country, sampling location, host species, host-identification level, pathogen group, pathogen species/genotype/serovar/strain, sample type, diagnostic method, number of *Apodemus* individuals or samples tested, number positive, prevalence or seroprevalence, study design, sampling context, sampling season or year, ecological determinants assessed, direction of reported associations, and evidence relevant to host-role interpretation.

Three related units were distinguished. A **unique publication** was a bibliographically distinct article counted once across the entire review. A **pathogen-specific article record** was an eligible article within one pathogen search block; consequently, the same publication could contribute one article record to more than one pathogen block. A **pathogen-study record** was an extracted pathogen-specific observation, normally representing a distinct combination of publication, pathogen, *Apodemus* taxon, country, diagnostic method, sample type and outcome. One pathogen-specific article record could therefore generate multiple pathogen-study records. Records identifying the host only as *Apodemus* spp. were retained at genus level and were not assigned retrospectively to a species.

Duplicate removal was performed separately within each of the 15 pathogen-specific search blocks. Duplicate database records for the same article within a block were removed, but an eligible publication retrieved for different pathogens was retained once in each relevant pathogen block. Consequently, the PRISMA diagram reports pathogen-specific article records rather than globally deduplicated publications. The Results additionally report the number of unique publications, while the supplementary evidence tables report pathogen-study records.

### 2.7 Evidence classification and synthesis

No formal numerical risk-of-bias score was used to exclude or rank studies, consistent with the evidence-mapping purpose of the scoping review. Instead, study interpretability was appraised descriptively according to sampling design, host-identification resolution, diagnostic method, denominator availability, pathogen taxonomic resolution, spatial resolution and whether ecological covariates or statistical associations were reported. These domains informed the interpretation of evidence strength but were not combined into a numerical quality score.

Evidence was classified using non-mutually exclusive descriptive categories. Detection-only evidence comprised direct identification of a pathogen, pathogen component or genetic material in an *Apodemus* sample without evidence of persistence, shedding or transmission. Exposure evidence comprised serological evidence indicating previous contact with the pathogen. Repeated association evidence referred to detection or exposure reported across multiple sites, years or independent studies. Ecological-association evidence required a reported or formally tested relationship between pathogen detection or exposure and a host, population, environmental or interface variable. Evidence consistent with reservoir involvement required repeated host association together with information relevant to pathogen maintenance, persistence, shedding or transmission. Detection, PCR positivity, seropositivity or prevalence alone was not interpreted as proof of reservoir competence.

Results were synthesised qualitatively by pathogen group and summarized according to *Apodemus* taxon, country, diagnostic method, number positive over number tested and detection or exposure outcome. Ecological-determinant information was charted when it was reported or formally tested in an otherwise eligible pathogen study; no separate systematic search for determinant studies was undertaken. Where authors explicitly reported an association, its direction was recorded as positive, negative, null or mixed/nonlinear, as appropriate. A determinant that was not reported or assessed was treated as unassessed rather than as evidence of no association. This component therefore maps reported ecological associations and does not constitute a complete denominator-based assessment of every determinant across all included records.

## 3 Results

### 3.1 Study selection

**Fig. 1:**
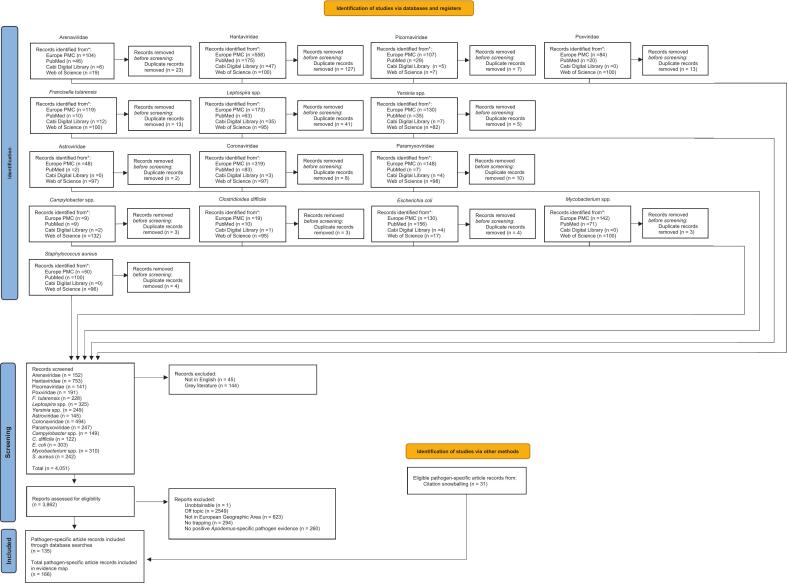
Flowchart summarising identification, screening, eligibility assessment and inclusion in the scoping review. Database searches retrieved 4317 records before within-block deduplication. Removal of 266 duplicates left 4051 pathogen-specific records for screening. At the initial screening stage, 190 records were excluded because they were in a language other than English, were grey literature, or were unobtainable, leaving 3861 records for assessment against the eligibility criteria. The combined total is verified, but the available screening information did not support a reliable retrospective allocation among the three reason categories. Eligibility assessment excluded 3726 records and retained 135 pathogen-specific article records from database searches. Backward citation searching added 31 eligible pathogen-specific article records, giving 166 eligible pathogen-specific article records, representing 141 unique publications and 241 extracted pathogen-study records. Counts are pathogen-specific records; duplicates were removed within rather than across pathogen blocks.

**Fig. 2:**
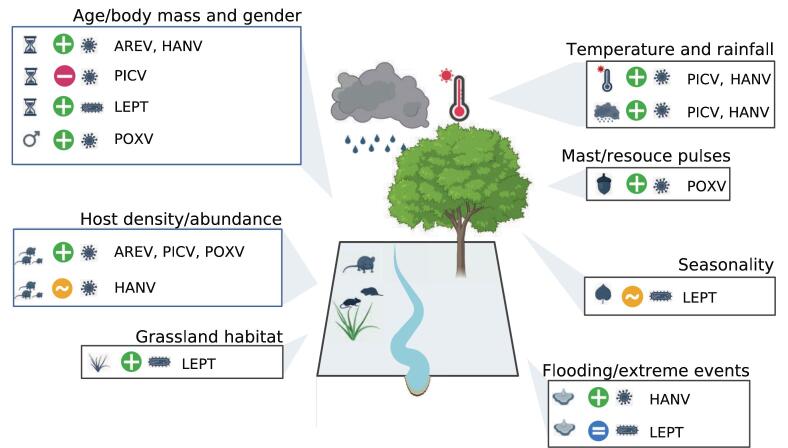
Selected ecological determinants reported in association with pathogen detection or exposure in European Apodemus populations. This evidence-map graphic visualises selected determinant–pathogen patterns reported by included studies; the supporting publications and complete determinant synthesis are provided in Table 3. Symbols show the directions reported in individual studies and do not represent pooled effect sizes, the number of supporting studies or overall strength of evidence. A green plus indicates a positive association; a red minus indicates a negative association; a blue equals sign indicates that no association was detected; and an orange wavy arrow indicates a mixed, nonlinear, lagged or temporally variable association. Abbreviations: AREV, arenaviruses; HANV, hantaviruses; LEPT, Leptospira spp.; PICV, picornaviruses; POXV, poxviruses. (For interpretation of the references to colour in this figure legend, the reader is referred to the web version of this article.)

After removal of duplicates within the 15 pathogen-specific search blocks, 4051 pathogen-specific records were screened. At the initial screening stage, 190 records were excluded because they were in a language other than English, were grey literature, or were unobtainable, leaving 3861 records for assessment against the eligibility criteria. The archived screening information did not support a reliable retrospective allocation of these 190 records among the three reason categories. Eligibility assessment retained 135 pathogen-specific article records from database searches, and backward citation searching added 31 eligible pathogen-specific article records, giving 166 eligible pathogen-specific article records in the evidence map (Fig. 1). These represented 141 unique publications and yielded 241 pathogen-study records in the detailed evidence tables (Supplementary Tables S3–S6). A publication retrieved under more than one pathogen block was counted once per relevant block, and an article could generate multiple pathogen-study records when it reported multiple host species, countries, diagnostic methods, sample types, or pathogen-specific outcomes. Consistent with the positive-evidence design, records without positive *Apodemus*-specific evidence were outside the review scope. (See Fig. 2.)

#### 3.1.1 Characteristics of included evidence

The evidence map covered 15 pathogen groups: seven viral groups and eight bacterial groups. Viral evidence comprised Arenaviridae, Hantaviridae, Picornaviridae, Poxviridae, Astroviridae, Coronaviridae and Paramyxoviridae. Bacterial evidence comprised *Francisella tularensis*, *Leptospira* spp., *Yersinia* spp., *Campylobacter* spp., *Clostridioides difficile*, *Escherichia coli*, *Mycobacterium* spp. and *Staphylococcus aureus*. The 166 pathogen-specific article records comprised 78 viral and 88 bacterial article records and generated 103 viral and 138 bacterial pathogen-study records, respectively (Table 1, Table 2).

**Table 1:** Study characteristics and evidence summary for viral pathogens reported in European *Apodemus* spp. *Counts are distinct source citations represented in the current detailed evidence tables for each pathogen group. †Ranges are based only on records with extractable denominators and are ordered by positivity proportion. Negative-only *Apodemus* records were excluded by design and are therefore not tabulated as zero-positive records. Positive/denominator ranges are reported only for records with extractable denominators. Detailed article-level records and source citations are provided in Supplementary Tables S3–S6.

Pathogen group	Pathogen-specific article records	Extracted pathogen-study records*	Countries represented	*Apodemus* species	Main assay types	Positive/denominator range†	Evidence strength / interpretive status
Arenaviridae	10	13	8	*A. agrarius; A. flavicollis; A. sylvaticus*	IFA; antibody assays; RT-PCR	6/217 to 4/16	Repeated exposure evidence; limited molecular confirmation
Hantaviridae	48	64	24	*A. agrarius; A. flavicollis; A. sylvaticus; Apodemus* spp.	ELISA; IFA; RT-PCR; PCR; WB; antibody assays; genetic sequencing	3/1416 to 16/28	Strongest viral evidence base; repeated molecular and serological detection
Picornaviridae	6	8	4	*A. agrarius; A. flavicollis; A. sylvaticus; Apodemus* spp.	RT-PCR; PCR; VNT; genetic sequencing	5/135 to 13/66	Limited detection/exposure evidence; host role unresolved
Poxviridae	5	9	4	*A. agrarius; A. flavicollis; A. sylvaticus*	IFA; SNT	2/47 to 14/22	Repeated exposure evidence; reservoir involvement plausible but not competence-proof
Astroviridae	2	2	2	*A. flavicollis*	Genetic sequencing	Not consistently extractable	Sparse discovery-oriented molecular evidence; host role unresolved
Coronaviridae	3	3	3	*A. sylvaticus; Apodemus* spp.	IFA; genetic sequencing	Not consistently extractable	Sparse serological/molecular evidence; host role unresolved
Paramyxoviridae	4	4	3	*A. flavicollis; A. sylvaticus*	RT-PCR; genetic sequencing	1/3 to 1/1	Sparse molecular detection; host role and zoonotic relevance unresolved

**Table 2:** Summary of bacterial pathogen evidence reported in European *Apodemus* spp. *Counts are distinct source citations represented in the current detailed evidence tables for each pathogen group. †Ranges are based only on records with extractable denominators and are ordered by positivity proportion. Negative-only *Apodemus* records were excluded by design and are therefore not tabulated as zero-positive records. Positive/denominator ranges are reported only for records with extractable denominators. Detailed article-level records and source citations are provided in Supplementary Tables S3–S6.

Pathogen group	Pathogen-specific article records	Extracted pathogen-study records	Countries represented	*Apodemus* species	Main assay types	Positive/denominator range†	Evidence strength / interpretive status
*Francisella tularensis*	13	18	7	*A. agrarius*; *A. flavicollis*; *A. sylvaticus*	PCR; RT-PCR; ELISA	1/336 to 15/39	Repeated detection/exposure; geographically localised and assay-dependent
*Leptospira* spp.	36	69	17	*A. agrarius*; *A. flavicollis*; *A. sylvaticus*; *A. uralensis*; *Apodemus* spp.	PCR; RT-PCR; MAT; antibody titre; serological assay	2/235 approx. to 7/7	Strongest bacterial evidence base; repeated molecular and serological detection
*Yersinia* spp.	8	12	5	*A. agrarius*; *A. flavicollis*; *A. sylvaticus*; *A. uralensis*	PCR; MAT	52/8793 to 2/7	Repeated detection of pathogenic and non-pathogenic taxa; host role unclear
*Campylobacter* spp.	6	9	3	*A. flavicollis*; *A. sylvaticus*; *A. uralensis*	PCR; culture; WGS; genetic sequencing	1/45 to 59/89	Interface carriage/dissemination evidence; primary reservoir status unsupported
*Clostridioides difficile*	3	3	2	*A. sylvaticus*; *Apodemus* spp.	PCR	1/19 to 2/13	Sparse molecular evidence; environmental carriage more likely than independent maintenance
*Escherichia coli*	11	14	10	*A. agrarius*; *A. flavicollis*; *A. sylvaticus*; *Apodemus* spp.	PCR; culture; WGS	5/482 to 3/7	AMR/virulence-carriage evidence; direct transmission role unclear
*Mycobacterium* spp.	4	5	5	*A. flavicollis*; *A. sylvaticus*	Culture; RT-PCR; PCR and culture	1/30 to 3/15	Sparse evidence; mainly environmental or opportunistic mycobacterial exposure.
*Staphylococcus aureus*	7	8	5	*A. agrarius*; *A. flavicollis*; *A. sylvaticus*	PCR; WGS	1/25 to 11/61	Carriage, strain diversity, and antimicrobial-resistance evidence at wildlife-human or wildlife-livestock interfaces.

Hantaviridae accounted for 48 pathogen-specific article records and 64 pathogen-study records, while *Leptospira* spp. accounted for 36 and 69, respectively. Each remaining pathogen group contributed 13 or fewer pathogen-specific article records. These frequencies reflect the distribution of published positive evidence and surveillance effort and should not be interpreted as standardised differences in pathogen prevalence or *Apodemus* reservoir competence.

Table 1, Table 2 summarise pathogen-specific article records, extracted pathogen-study records, countries represented, *Apodemus* taxa, principal diagnostic methods, positive/denominator ranges and the interpretive status of the evidence for each pathogen group. Detailed denominator-based and detection-only records are provided in Supplementary Tables S3–S6.

### 3.2 Host species and geographic patterns

Across the 241 pathogen-study records, *A. flavicollis* was represented in 92 records, *A. sylvaticus* in 68, *A. agrarius* in 63 and *A. uralensis* in three; 15 records identified the host only as *Apodemus* spp. These are counts of pathogen-study records rather than unique publications, studies or individual animals. The greater representation of particular host taxa therefore reflects the distribution of published positive evidence and sampling effort and should not be interpreted as comparative reservoir competence.

Positive *Apodemus*-associated pathogen evidence was reported from 28 European countries. When multi-country records were counted for each country listed, the most frequently represented countries were Croatia (31 pathogen-study records), Germany (28), Czechia (22), Hungary (13), Italy, Poland and the United Kingdom (12 each), Finland (11), and France, Slovakia and Ukraine (10 each). The remaining countries were represented by fewer than 10 pathogen-study records. Geographic differences reflect uneven surveillance, sampling and publication effort rather than standardised differences in pathogen occurrence or prevalence.

### 3.3 Diagnostic methods and evidence type

Across the 241 pathogen-study records, PCR was reported in 92 records, RT-PCR in 44, IFA or indirect immunofluorescence in 28, MAT in 21 and ELISA in 17. Genetic sequencing was reported in 12 records, WGS in four and culture alone in four. Seventeen additional records used other antibody, serological, neutralisation or Western blot assays; one reported combined PCR and culture, and one did not specify the diagnostic method. These are pathogen-study-record counts rather than numbers of unique publications, and an article using multiple diagnostic approaches could contribute more than one record.

Serological findings were interpreted as evidence of exposure rather than current infection, pathogen shedding or host competence. PCR and RT-PCR indicated the presence of pathogen nucleic acid but did not by themselves demonstrate organism viability or transmission. Culture provided evidence of viable organisms, while sequencing and WGS increased pathogen, genotype or strain resolution. No diagnostic method, considered alone, was interpreted as proof of reservoir competence.

### 3.4 Ecological determinants of infection and exposure

Ecological-determinant information was reported or tested in 26 of the 141 unique included publications (Table 3). The same publication could contribute evidence to more than one determinant domain. Because determinant information was charted when reported in otherwise eligible pathogen studies, rather than identified through a separate determinant-specific search, the numbers below represent supporting included publications and not the total number of studies that could potentially have assessed each determinant. Variables that were not reported were treated as unassessed rather than as null associations.

**Table 3:** Reported ecological determinants of *Apodemus*-associated pathogen detection or exposure in the included evidence base. *n* denotes the number of unique included publications reporting or testing each determinant. Individual publications could contribute to more than one determinant domain; consequently, the row totals do not sum to the 26 publications containing ecological-determinant information. Variables not reported were treated as unassessed rather than as null associations.

Determinant	Pathogen(s)	Studies reporting or testing the determinant	Reported direction or pattern
Sex	Arenaviridae; Hantaviridae; Poxviridae; *Leptospira* spp.	[75], [77], [107], [108], [116], [144], [145]	Mostly male-biased where reported; variable among pathogens
Age/body mass	Arenaviridae; Hantaviridae; Poxviridae; Picornaviridae; *Leptospira* spp.	[75], [77], [82], [107], [108], [116], [144], [145]	Generally higher positivity in older/heavier individuals; exception reported for picornaviruses/Ljungan virus
Reproductive status	Arenaviridae; *Leptospira* spp.; possibly Hantaviridae	[75], [107], [108], [145]	Positive or mixed association depending on pathogen and season
Host density/abundance	Arenaviridae; Hantaviridae; Poxviridae; Picornaviridae; *Leptospira* spp.	[63], [75], [80], [107], [108], [111]	Positive, lagged, nonlinear, or temporally variable
Season	Hantaviridae; Poxviridae; Picornaviridae; *Leptospira* spp.; Arenaviridae	[63], [75], [80], [107], [108], [111], [145]	Seasonal variation reported, but peak timing differs among pathogens and sites
Mast/resource pulses	Poxviridae	[80]	Usually positive or lagged through host demographic increase
Habitat type	Hantaviridae; Poxviridae; *Leptospira* spp.	[52], [80], [116], [146], [147], [148]	Mixed; forest, grassland, and habitat-type effects vary by pathogen
Forest cover/land use	Hantaviridae; *Leptospira* spp.; *Campylobacter* spp.; *E. coli*; *S. aureus*; *C. difficile*	[52], [61], [92], [100], [102], [103], [116], [148]	Mixed; natural, agricultural, peri-urban, and farm-interface contexts linked to different pathogen systems
Rainfall/temperature/humidity	Hantaviridae; Picornaviridae; *Leptospira* spp.	[63], [82], [107], [108], [116]	Mostly positive or mixed; often indirect through host survival, abundance, or environmental persistence
Flooding/extreme events	Hantaviridae; *Leptospira* spp.	[119], [120]	Mixed; higher hantavirus positivity in flood-affected areas, no clear *Leptospira* difference in cited example
Synanthropy/urban or farm exposure	*Leptospira* spp.; *Campylobacter* spp.; *C. difficile*; *E. coli*; *Mycobacterium* spp.; *S. aureus*; *Yersinia* spp.	[61], [64], [92], [98], [100], [102], [103], [107], [108], [117], [149]	Mostly descriptive or interface-associated; rarely causal

Host-level determinants included sex, reported in seven included publications; age or body mass, reported in eight; and reproductive status, reported in four. Higher positivity in older or heavier individuals was reported for Arenaviridae, Hantaviridae, Poxviridae and *Leptospira* spp., whereas a contrasting sub-adult-biased pattern was reported for Picornaviridae. Sex- and reproductive-status associations were inconsistent and should not be generalised across pathogen groups.

Population-level determinants included host density or abundance in six publications, season in seven and mast or other resource pulses in one. Reported relationships were variously positive, delayed, nonlinear or temporally variable. Seasonal associations were reported for Arenaviridae, Hantaviridae, Poxviridae, Picornaviridae and *Leptospira* spp., but the timing and direction of peaks differed among pathogens and study locations.

Environmental and interface determinants included habitat type in six publications, forest cover or land use in eight, rainfall, temperature or humidity in five, flooding or extreme events in two, and synanthropic, urban or farm exposure in 11. Directions were mixed and context-dependent. For foodborne, environmental and interface-associated bacteria, the evidence was generally descriptive of exposure or carriage settings rather than demonstrative of independent pathogen maintenance by *Apodemus* populations.

## 4 Discussion

### 4.1 Main findings and evidence gradient

This scoping review mapped positive evidence of zoonotic viral and bacterial pathogens in European *Apodemus* populations. Because negative-only records were outside the review scope, comparisons reflect the breadth of published positive evidence rather than pathogen occurrence or absence.

Evidence was strongest and most geographically extensive for Hantaviridae and *Leptospira* spp., with repeated molecular and serological findings across European countries; however, detection or exposure did not establish reservoir competence for every host–pathogen combination [49], [50], [51], [52], [53], [54], [55]. Evidence for Arenaviridae, Poxviridae, *Francisella tularensis*, *Yersinia* spp., *Campylobacter* spp., *Escherichia coli* and *Staphylococcus aureus* was moderate or context-dependent, whereas evidence for Astroviridae, Coronaviridae, Paramyxoviridae, *Clostridioides difficile* and *Mycobacterium* spp. was sparse or discovery-oriented [56], [57], [58], [59], [60], [61], [62], [63], [64].

The predominance of *A. flavicollis*, *A. sylvaticus* and *A. agrarius* in the evidence base is consistent with their broad European distributions and frequent occurrence in natural, agricultural, peri-domestic and urban-interface settings. Nevertheless, their greater representation may also reflect geographic accessibility, research priorities and unequal sampling effort. It should therefore not be interpreted as evidence that these species have greater reservoir competence than less frequently studied *Apodemus* taxa [24], [25], [29], [30], [31].

An evidence gradient was used: serology indicated exposure; molecular or culture-based findings supported detection or carriage; and repeated evidence across studies, sites or periods strengthened host-association inference. Reservoir involvement required additional evidence of maintenance, persistence, shedding or onward transmission, distinguishing it from spillover, transient carriage or sentinel detection.

### 4.2 Pathogen-specific interpretation

For Hantaviridae, repeated molecular and serological evidence across several *Apodemus* taxa supported host associations, although inference varied by virus, host, region and assay [53], [65], [66], [67], [68], [69]. For pathogenic *Leptospira* taxa, reservoir attribution additionally requires evidence of renal colonisation, persistence, urinary shedding and transmission [50], [51], [54], [70], [71].

Arenavirus evidence in *A. sylvaticus* and *A. flavicollis* indicated exposure or detection despite the primary association of LCMV with *Mus musculus* [72], [73], [74], [75]. Cowpox findings supported woodland-rodent involvement, but outcomes, seasonality and host-fitness effects varied [76], [77], [78], [79], [80]. Picornavirus detections, including Ljungan virus and encephalomyocarditis virus, were too limited to resolve reservoir involvement or zoonotic relevance [81], [82], [83], [84].

*Francisella tularensis* findings suggested participation in environmental, arthropod-associated, waterborne and multi-host circulation rather than independent maintenance by *Apodemus* [85], [86], [87], [88], [89], [90]. For *Yersinia*, species-resolved *Y. enterocolitica* or *Y. pseudotuberculosis* records were considered potentially zoonotic, whereas pooled or unresolved *Yersinia* records provided detection context only [91], [92], [93], [94], [95], [96].

For *Campylobacter* spp., *Clostridioides difficile*, *Escherichia coli*, *Mycobacterium* spp. and *Staphylococcus aureus*, an interface-host framework was more appropriate than a strict reservoir framework. The evidence supported carriage, strain sharing, antimicrobial-resistance surveillance or exposure at farm, peri-urban and wildlife–livestock interfaces. It did not consistently demonstrate independent maintenance or direct transmission from *Apodemus* populations to humans or livestock [61], [92], [97], [98], [99], [100], [101], [102], [103], [104].

Astroviridae, Coronaviridae and Paramyxoviridae were represented primarily by sparse molecular, metagenomic or serological records. These findings are best interpreted as host-range or pathogen-discovery evidence unless supported by repeated detection, tissue tropism, shedding, viable-virus recovery and epidemiological linkage. Their maintenance within European *Apodemus* populations and their zoonotic significance therefore remain unresolved [56], [58], [59], [60], [62], [63], [105], [106].

### 4.3 Diagnostic and taxonomic limits of inference

Diagnostic heterogeneity limited cross-study comparison because serology, nucleic-acid detection, culture and sequencing provide different forms of evidence, and positivity estimates from different assays or sample types are not equivalent. Repeated serological and molecular evidence strengthened host-association inference for hantaviruses and arenaviruses but did not establish reservoir competence [49], [53], [55], [74], [75]. Renal-tissue or urine detection was more informative than serology for *Leptospira* shedding [51], [52], [54], [70], [71], while strain typing or WGS was needed to assess interface-bacteria transmission [98], [99], [100], [101], [102], [104]. Metagenomic viral detections remained discovery evidence without repeated detection, shedding or epidemiological linkage [58], [59], [60], [62], [63], [106].

Genus-level *Apodemus* records could not be assigned retrospectively to species, reducing ecological precision because major taxa differ in distribution, habitat use, abundance and synanthropy [24], [25], [29]. Evidence was therefore interpreted from exposure or detection through repeated host association to reservoir-consistent evidence when diagnostic, ecological and transmission information converged.

### 4.4 Ecological determinants of infection and exposure

Table 3 provides a qualitative map of reported associations; unassessed determinants cannot be interpreted as null associations. Older or heavier animals often showed greater positivity, but Ljungan virus showed a sub-adult bias, and sex or reproductive associations were inconsistent [75], [77], [82], [107], [108]. Density and reproductive activity were associated with arenavirus transmission, while hantavirus patterns were seasonal, lagged or nonlinear [75], [109], [110], [111]. Cowpox provided an included mast example, while broader hantavirus-mast relationships were contextual [14], [45], [46], [80], [112], seasonal peaks differed among pathogens and locations [49], [107], [113], [114].

Habitat and land-use associations varied among hantavirus, poxvirus and *Leptospira* studies [42], [44], [98], [107], [108], [113], [114], [115], [116], while farm, urban and peri-urban findings for *Campylobacter*, *C. difficile*, *E. coli*, *Mycobacterium* and *S. aureus* more often indicated exposure or contamination interfaces than independent maintenance [61], [64], [92], [97], [98], [101], [102], [103], [117]. Temperature, rainfall and humidity acted directly through environmental persistence or indirectly through host demography, with context-dependent directions and timing [14], [19], [63], [82], [107], [108], [116], [118]; flood-area studies reported higher hantavirus positivity but no corresponding *Leptospira* difference [119], [120].

### 4.5 Climate change, synanthropy, and One Health implications

The evidence supports viewing European *Apodemus* species as ecological interface hosts rather than treating them uniformly as classical reservoirs. Their occurrence across forests, grasslands, agricultural landscapes, peri-domestic environments and urban interfaces allows them to connect habitat conditions, environmental contamination, domestic animals and human exposure [24], [25], [30], [31]. For directly transmitted viruses, particularly hantaviruses and arenaviruses, risk is linked to aerosolised excreta and contaminated nesting or occupational environments, including forestry, agriculture and entry into rodent-contaminated buildings [49], [115], [121], [122]. Cowpox virus adds a companion-animal bridge because domestic cats may acquire infection from wild rodents and transmit it to humans [123], [124].

For environmentally mediated bacteria, *Apodemus* spp. may be more important as carriers or sentinels of contamination than as independent reservoirs. *Leptospira* spp. provide the clearest example because renal shedding can contaminate water, soil or food substrates [70], [71], [108]. Detection of *Campylobacter* spp., *C. difficile*, *E. coli* and *S. aureus* often reflects farm or peri-urban exposure, environmental dissemination or antimicrobial-resistance pathways [61], [92], [97], [98], [101], [102], [103], [104], [125].

Climate and land-use change can modify rodent abundance, distribution, survival and contact with people, livestock and domestic animals, but their effects are species- and ecosystem-specific and may operate indirectly through resources and trophic interactions [12], [37], [126], [127], [128], [129], [130]. *A. agrarius* may expand westward or northwestward and increasingly occupy human-modified environments, while *A. flavicollis* shows climate-, mast- and resource-associated demographic responses [40], [41], [131], [132], [133], [134]. Altered rainfall, snow cover, spring melt and mast dynamics may therefore reshape transmission opportunities, although responses can be nonlinear and locally contingent [135], [136], [137], [138], [139].

Urbanisation and synanthropy can further increase contact among wild rodents, domestic animals, livestock and people, while farm and peri-urban environments facilitate microbial exchange across host and environmental compartments [140], [141], [142].

In this context, an operational One Health framework should integrate:

• standardised wild-rodent trapping and reliable *Apodemus* identification, including molecular confirmation where morphology is ambiguous;
• pathogen diagnostics reported with assay metadata, sample type, denominators and positive counts;
• environmental covariates, including land cover, mast years, rainfall, temperature, flooding history and synanthropy;
• veterinary, public-health and human-exposure data from relevant occupational, agricultural and peri-urban settings; and
• compatible spatial databases and sampling periods capable of supporting early-warning models.

Without such integration, wildlife detections, environmental drivers, veterinary findings and human cases remain fragmented, limiting identification of the conditions associated with zoonotic spillover.

### 4.6 Limitations

This review was designed to map documented positive *Apodemus*-associated pathogen evidence. Studies reporting exclusively negative *Apodemus* results were outside its scope; consequently, the review cannot estimate pathogen absence, zero prevalence or the relative frequency of positive and negative studies. The positive-evidence design may overrepresent reported host–pathogen associations and should not be interpreted as a prevalence-standardised synthesis or comparative assessment of reservoir competence.

Study selection and data extraction were performed by one reviewer, so selection errors, extraction errors and subjective eligibility decisions cannot be excluded. No review protocol was registered, and no formal numerical risk-of-bias tool was applied. Predefined eligibility criteria, Rayyan-assisted screening, full-text assessment of uncertain records and descriptive appraisal of study interpretability reduced but did not eliminate these risks.

Searches and duplicate removal were organised within pathogen-specific blocks rather than globally across the review. The PRISMA diagram therefore reports pathogen-specific article records, which are not equivalent to globally unique publications. Although the number of unique publications is reported separately, these counts are not directly comparable with reviews using global deduplication.

The evidence base was heterogeneous in sampling design, diagnostic method, sample type, host-identification resolution, denominator availability, spatial scale and reporting detail, precluding pooled prevalence or effect estimation. Serology, PCR, RT-PCR, culture, sequencing, WGS and metagenomics provide different evidence and cannot be treated as equivalent measures of infection, exposure, shedding or transmission. Detection or seropositivity alone does not demonstrate reservoir competence.

Host and geographic representation were also uneven. Genus-level *Apodemus* records could not be assigned retrospectively to species, reducing ecological precision because *Apodemus* taxa differ in distribution, habitat use and synanthropic behaviour [24], [25], [29]. Differences among host species, countries and pathogen groups also reflect unequal surveillance and publication effort rather than standardised epidemiological differences. Geographic and pathogen-group gaps should therefore be interpreted cautiously and may reflect surveillance deficits rather than pathogen absence.

Ecological determinants were not assessed consistently, and no separate systematic search for determinant studies was undertaken. Many publications reported pathogen occurrence without testing host, population, habitat, climatic or interface variables. Table 3 therefore maps determinants reported or tested in identified studies; it does not provide a complete denominator-based or meta-analytic estimate of their direction or effect strength.

Finally, the review was restricted to peer-reviewed English-language literature from Europe published between 2000 and 2025. Relevant evidence in non-English publications, grey literature, surveillance reports, theses and local public-health datasets may have been missed. This restriction, together with the positive-evidence design, increases susceptibility to geographic and publication bias.

### 4.7 Research priorities

Future research should move beyond opportunistic detection toward standardised longitudinal designs capable of distinguishing exposure, transient carriage, persistent infection, shedding and transmission. Studies should use reproducible trapping effort, reliable *Apodemus* identification, supported by molecular confirmation where necessary, pathogen-resolved diagnostics, explicit denominators and repeated sampling. Reservoir competence should be evaluated using evidence of persistence, viable-pathogen shedding and transmission rather than detection or seropositivity alone.

Ecological covariates should be specified before analysis and recorded consistently, including age or body mass, sex, reproductive status, host abundance, season, habitat, land use, mast or other resource pulses, rainfall, temperature, humidity, flooding and synanthropic exposure. Studies should report positive, negative, null and mixed associations, together with the statistical methods and temporal lags examined. This would permit denominator-based comparisons and help distinguish recurrent determinants from isolated or context-specific observations.

Surveillance should integrate rodent, environmental, veterinary and human-exposure data. For hantaviruses and arenaviruses, priorities include host abundance, species identity, infection or exposure status, habitat management, occupational exposure and excreta-contamination risk [115], [122], [140]. For *Leptospira* spp. and interface-associated bacteria, monitoring should include water, soil, livestock, farm infrastructure, antimicrobial resistance and peri-urban exposure [70], [92], [100], [101], [102], [108]. Harmonised spatial units and sampling periods would support early-warning models, especially in regions affected by *A. agrarius* expansion, *A. flavicollis* demographic change, altered mast regimes, changing rainfall and increasing urban or farm contact [40], [41], [131], [132], [133], [143].

## 5 Conclusion

This scoping review found that European *Apodemus*-associated evidence was strongest for Hantaviridae and *Leptospira* spp., context-dependent for Arenaviridae, Poxviridae and several interface-associated bacteria, and sparse or primarily detection-oriented for other viral and bacterial groups. Reported determinants included host demography, abundance, seasonality, habitat, land use, resource pulses and climate, but few were tested using comparable designs. Accordingly, pathogen detection or seropositivity should not be interpreted as proof of reservoir competence. European *Apodemus* species are best viewed as potential reservoirs in specific pathogen systems and, more broadly, as ecological interface hosts linking wildlife, environmental contamination, livestock or peri-urban settings and human exposure. One Health surveillance should therefore integrate standardised host identification and pathogen diagnostics with ecological, environmental, veterinary, public-health and human-exposure data within shared spatial and temporal frameworks.

## Declaration of generative AI and AI-assisted technologies in the manuscript preparation process

During preparation of this manuscript, the authors used generative AI-assisted tools for language editing, concision, and table and figure caption drafting. After using these tools, the authors reviewed and edited the content as needed and take full responsibility for the published manuscript.

## CRediT authorship contribution statement

**Daniele Fabbri:** Writing – review & editing, Writing – original draft, Validation, Project administration, Investigation, Formal analysis, Data curation, Conceptualization. **Laura Grassi:** Writing – review & editing, Methodology, Investigation, Conceptualization. **Luca Dorigo:** Writing – review & editing, Writing – original draft, Methodology, Data curation. **Stefania Leopardi:** Writing – review & editing. **Valentina Tagliapietra:** Writing – review & editing. **Paola Beraldo:** Writing – review & editing, Supervision, Resources, Project administration, Funding acquisition, Conceptualization.

## Funding

This activity was part of the National Biodiversity Future Center (NBFC), Project funded under the National Recovery and Resilience Plan (NRRP), Mission 4 Component 2 Investment 1.4 - Call for tender No. 3138 of 16 December 2021, rectified by Decree n.3175 of 18 December 2021 of Italian Ministry of University and Research funded by the European Union – NextGenerationEU (Project code CN_00000033).

## Declaration of competing interest

The authors declare that they have no known competing financial interests or personal relationships that could have appeared to influence the work reported in this paper.

## Data Availability

All data extracted and synthesised in this scoping review are provided within the article and Supplementary Tables S3–S6. The complete database search strategies and eligibility criteria are provided in Supplementary Tables S1 and S2, respectively. No new primary data were generated.

## Appendix A Supplementary data

Supplementary material 1 Database-specific search strategies and pathogen-block retrieval totals used for the structured literature search. Formal searches across PubMed, Europe PMC, Web of Science, and CABI Digital Library covered 15 pathogen blocks; after within-block deduplication, these searches yielded the 4,051 records screened in the PRISMA flow diagram. IMAGE 1

Supplementary material 2 Inclusion and exclusion criteria applied during study selection. The review mapped documented positive Apodemus-associated evidence of zoonotic viral and bacterial pathogens; studies reporting exclusively negative results were outside the scope of the main evidence map. IMAGE 2

Supplementary material 3 Denominator-based records of viral pathogen detection in European Apodemus populations. Positive records with extractable denominators are reported as n/N and percentage (n/N × 100), rounded to one decimal place. Broad pathogen labels denote records without species- or genotype-level resolution. †Sample-level result, where n/N refers to positive samples rather than animals; ns, not specified. Positive Apodemus-specific records without extractable denominators are presented separately in the detection-only supplementary data. IMAGE 3
