# Supplementary material for: Small rodents, huge impact: A scoping review of European Apodemus species in the ecology of directly transmitted, environmentally mediated, and interface-associated zoonotic pathogens
Source: One Health. 2026 Jul 28;23:101532. doi: 10.1016/j.onehlt.2026.101532 (PMC13452164; doi:10.1016/j.onehlt.2026.101532)
Supplement: Supplementary file 1 [file mmc1.docx]

# Supplementary Material

## Table S1. Database-specific search queries used for structured literature retrieval

The pathogen-specific database searches constituted the formal retrieval stream used for PRISMA accounting. Broad host–pathogen sensitivity searches and selected country–pathogen combinations were used only as sensitivity checks; they identified no additional eligible records and were not counted as a separate retrieval stream. Ecological-determinant information was charted when reported in otherwise eligible pathogen studies; no separate systematic determinant search was undertaken. The final formal database searches were completed in December 2025. All search queries featured the same limitations: Publication years 2000-2025; English-language and peer-reviewed primary-study restrictions applied during screening.

## Arenaviridae / LCMV

| **Database** | **Full search query** |
| --- | --- |
| PubMed | (Apodemus[Title/Abstract] OR "wood mouse"[Title/Abstract] OR "yellow-necked mouse"[Title/Abstract] OR "striped field mouse"[Title/Abstract]) AND (Arenaviridae[Title/Abstract] OR arenavirus[Title/Abstract] OR arenaviruses[Title/Abstract] OR "lymphocytic choriomeningitis virus"[Title/Abstract] OR LCMV[Title/Abstract] OR Mammarenavirus[Title/Abstract] OR "Mammarenavirus choriomeningitidis"[Title/Abstract]) AND (Europe[Title/Abstract] OR European[Title/Abstract] OR Austria[Title/Abstract] OR Belarus[Title/Abstract] OR Belgium[Title/Abstract] OR Bosnia[Title/Abstract] OR Bulgaria[Title/Abstract] OR Croatia[Title/Abstract] OR Czechia[Title/Abstract] OR "Czech Republic"[Title/Abstract] OR Denmark[Title/Abstract] OR Estonia[Title/Abstract] OR Finland[Title/Abstract] OR France[Title/Abstract] OR Germany[Title/Abstract] OR Greece[Title/Abstract] OR Hungary[Title/Abstract] OR Ireland[Title/Abstract] OR Italy[Title/Abstract] OR Latvia[Title/Abstract] OR Lithuania[Title/Abstract] OR Luxembourg[Title/Abstract] OR Moldova[Title/Abstract] OR Montenegro[Title/Abstract] OR Netherlands[Title/Abstract] OR Norway[Title/Abstract] OR Poland[Title/Abstract] OR Portugal[Title/Abstract] OR Romania[Title/Abstract] OR Serbia[Title/Abstract] OR Slovakia[Title/Abstract] OR Slovenia[Title/Abstract] OR Spain[Title/Abstract] OR Sweden[Title/Abstract] OR Switzerland[Title/Abstract] OR Ukraine[Title/Abstract] OR "United Kingdom"[Title/Abstract] OR England[Title/Abstract] OR Scotland[Title/Abstract] OR Wales[Title/Abstract]) |
| Europe PMC | (TITLE_ABS:Apodemus OR TITLE_ABS:"wood mouse" OR TITLE_ABS:"yellow-necked mouse" OR TITLE_ABS:"striped field mouse") AND (TITLE_ABS:Arenaviridae OR TITLE_ABS:arenavirus OR TITLE_ABS:arenaviruses OR TITLE_ABS:"lymphocytic choriomeningitis virus" OR TITLE_ABS:LCMV OR TITLE_ABS:Mammarenavirus OR TITLE_ABS:"Mammarenavirus choriomeningitidis") AND (TITLE_ABS:Europe OR TITLE_ABS:European OR TITLE_ABS:Austria OR TITLE_ABS:Belarus OR TITLE_ABS:Belgium OR TITLE_ABS:Bosnia OR TITLE_ABS:Bulgaria OR TITLE_ABS:Croatia OR TITLE_ABS:Czechia OR TITLE_ABS:"Czech Republic" OR TITLE_ABS:Denmark OR TITLE_ABS:Estonia OR TITLE_ABS:Finland OR TITLE_ABS:France OR TITLE_ABS:Germany OR TITLE_ABS:Greece OR TITLE_ABS:Hungary OR TITLE_ABS:Ireland OR TITLE_ABS:Italy OR TITLE_ABS:Latvia OR TITLE_ABS:Lithuania OR TITLE_ABS:Luxembourg OR TITLE_ABS:Moldova OR TITLE_ABS:Montenegro OR TITLE_ABS:Netherlands OR TITLE_ABS:Norway OR TITLE_ABS:Poland OR TITLE_ABS:Portugal OR TITLE_ABS:Romania OR TITLE_ABS:Serbia OR TITLE_ABS:Slovakia OR TITLE_ABS:Slovenia OR TITLE_ABS:Spain OR TITLE_ABS:Sweden OR TITLE_ABS:Switzerland OR TITLE_ABS:Ukraine OR TITLE_ABS:"United Kingdom" OR TITLE_ABS:England OR TITLE_ABS:Scotland OR TITLE_ABS:Wales) |
| Web of Science | TS=((Apodemus OR "wood mouse" OR "yellow-necked mouse" OR "striped field mouse") AND (Arenaviridae OR arenavirus OR arenaviruses OR "lymphocytic choriomeningitis virus" OR LCMV OR Mammarenavirus OR "Mammarenavirus choriomeningitidis") AND (Europe OR European OR Austria OR Belarus OR Belgium OR Bosnia OR Bulgaria OR Croatia OR Czechia OR "Czech Republic" OR Denmark OR Estonia OR Finland OR France OR Germany OR Greece OR Hungary OR Ireland OR Italy OR Latvia OR Lithuania OR Luxembourg OR Moldova OR Montenegro OR Netherlands OR Norway OR Poland OR Portugal OR Romania OR Serbia OR Slovakia OR Slovenia OR Spain OR Sweden OR Switzerland OR Ukraine OR "United Kingdom" OR England OR Scotland OR Wales)) |
| CABI Digital Library | (Apodemus OR "wood mouse" OR "yellow-necked mouse" OR "striped field mouse") AND (Arenaviridae OR arenavirus OR arenaviruses OR "lymphocytic choriomeningitis virus" OR LCMV OR Mammarenavirus OR "Mammarenavirus choriomeningitidis") AND (Europe OR European OR Austria OR Belarus OR Belgium OR Bosnia OR Bulgaria OR Croatia OR Czechia OR "Czech Republic" OR Denmark OR Estonia OR Finland OR France OR Germany OR Greece OR Hungary OR Ireland OR Italy OR Latvia OR Lithuania OR Luxembourg OR Moldova OR Montenegro OR Netherlands OR Norway OR Poland OR Portugal OR Romania OR Serbia OR Slovakia OR Slovenia OR Spain OR Sweden OR Switzerland OR Ukraine OR "United Kingdom" OR England OR Scotland OR Wales) |

## Hantaviridae / orthohantaviruses

| **Database** | **Full search query** |
| --- | --- |
| PubMed | (Apodemus[Title/Abstract] OR "wood mouse"[Title/Abstract] OR "yellow-necked mouse"[Title/Abstract] OR "striped field mouse"[Title/Abstract]) AND (Hantaviridae[Title/Abstract] OR hantavirus[Title/Abstract] OR hantaviruses[Title/Abstract] OR Orthohantavirus[Title/Abstract] OR orthohantavirus[Title/Abstract] OR Dobrava[Title/Abstract] OR "Dobrava-Belgrade"[Title/Abstract] OR DOBV[Title/Abstract] OR Saaremaa[Title/Abstract] OR SAAV[Title/Abstract] OR Kurkino[Title/Abstract] OR KURV[Title/Abstract] OR Sochi[Title/Abstract] OR SOCV[Title/Abstract] OR Hantaan[Title/Abstract] OR Tula[Title/Abstract]) AND (Europe[Title/Abstract] OR European[Title/Abstract] OR Austria[Title/Abstract] OR Belarus[Title/Abstract] OR Belgium[Title/Abstract] OR Bosnia[Title/Abstract] OR Bulgaria[Title/Abstract] OR Croatia[Title/Abstract] OR Czechia[Title/Abstract] OR "Czech Republic"[Title/Abstract] OR Denmark[Title/Abstract] OR Estonia[Title/Abstract] OR Finland[Title/Abstract] OR France[Title/Abstract] OR Germany[Title/Abstract] OR Greece[Title/Abstract] OR Hungary[Title/Abstract] OR Ireland[Title/Abstract] OR Italy[Title/Abstract] OR Latvia[Title/Abstract] OR Lithuania[Title/Abstract] OR Luxembourg[Title/Abstract] OR Moldova[Title/Abstract] OR Montenegro[Title/Abstract] OR Netherlands[Title/Abstract] OR Norway[Title/Abstract] OR Poland[Title/Abstract] OR Portugal[Title/Abstract] OR Romania[Title/Abstract] OR Serbia[Title/Abstract] OR Slovakia[Title/Abstract] OR Slovenia[Title/Abstract] OR Spain[Title/Abstract] OR Sweden[Title/Abstract] OR Switzerland[Title/Abstract] OR Ukraine[Title/Abstract] OR "United Kingdom"[Title/Abstract] OR England[Title/Abstract] OR Scotland[Title/Abstract] OR Wales[Title/Abstract]) |
| Europe PMC | (TITLE_ABS:Apodemus OR TITLE_ABS:"wood mouse" OR TITLE_ABS:"yellow-necked mouse" OR TITLE_ABS:"striped field mouse") AND (TITLE_ABS:Hantaviridae OR TITLE_ABS:hantavirus OR TITLE_ABS:hantaviruses OR TITLE_ABS:Orthohantavirus OR TITLE_ABS:orthohantavirus OR TITLE_ABS:Dobrava OR TITLE_ABS:"Dobrava-Belgrade" OR TITLE_ABS:DOBV OR TITLE_ABS:Saaremaa OR TITLE_ABS:SAAV OR TITLE_ABS:Kurkino OR TITLE_ABS:KURV OR TITLE_ABS:Sochi OR TITLE_ABS:SOCV OR TITLE_ABS:Hantaan OR TITLE_ABS:Tula) AND (TITLE_ABS:Europe OR TITLE_ABS:European OR TITLE_ABS:Austria OR TITLE_ABS:Belarus OR TITLE_ABS:Belgium OR TITLE_ABS:Bosnia OR TITLE_ABS:Bulgaria OR TITLE_ABS:Croatia OR TITLE_ABS:Czechia OR TITLE_ABS:"Czech Republic" OR TITLE_ABS:Denmark OR TITLE_ABS:Estonia OR TITLE_ABS:Finland OR TITLE_ABS:France OR TITLE_ABS:Germany OR TITLE_ABS:Greece OR TITLE_ABS:Hungary OR TITLE_ABS:Ireland OR TITLE_ABS:Italy OR TITLE_ABS:Latvia OR TITLE_ABS:Lithuania OR TITLE_ABS:Luxembourg OR TITLE_ABS:Moldova OR TITLE_ABS:Montenegro OR TITLE_ABS:Netherlands OR TITLE_ABS:Norway OR TITLE_ABS:Poland OR TITLE_ABS:Portugal OR TITLE_ABS:Romania OR TITLE_ABS:Serbia OR TITLE_ABS:Slovakia OR TITLE_ABS:Slovenia OR TITLE_ABS:Spain OR TITLE_ABS:Sweden OR TITLE_ABS:Switzerland OR TITLE_ABS:Ukraine OR TITLE_ABS:"United Kingdom" OR TITLE_ABS:England OR TITLE_ABS:Scotland OR TITLE_ABS:Wales) |
| Web of Science | TS=((Apodemus OR "wood mouse" OR "yellow-necked mouse" OR "striped field mouse") AND (Hantaviridae OR hantavirus OR hantaviruses OR Orthohantavirus OR orthohantavirus OR Dobrava OR "Dobrava-Belgrade" OR DOBV OR Saaremaa OR SAAV OR Kurkino OR KURV OR Sochi OR SOCV OR Hantaan OR Tula) AND (Europe OR European OR Austria OR Belarus OR Belgium OR Bosnia OR Bulgaria OR Croatia OR Czechia OR "Czech Republic" OR Denmark OR Estonia OR Finland OR France OR Germany OR Greece OR Hungary OR Ireland OR Italy OR Latvia OR Lithuania OR Luxembourg OR Moldova OR Montenegro OR Netherlands OR Norway OR Poland OR Portugal OR Romania OR Serbia OR Slovakia OR Slovenia OR Spain OR Sweden OR Switzerland OR Ukraine OR "United Kingdom" OR England OR Scotland OR Wales)) |
| CABI Digital Library | (Apodemus OR "wood mouse" OR "yellow-necked mouse" OR "striped field mouse") AND (Hantaviridae OR hantavirus OR hantaviruses OR Orthohantavirus OR orthohantavirus OR Dobrava OR "Dobrava-Belgrade" OR DOBV OR Saaremaa OR SAAV OR Kurkino OR KURV OR Sochi OR SOCV OR Hantaan OR Tula) AND (Europe OR European OR Austria OR Belarus OR Belgium OR Bosnia OR Bulgaria OR Croatia OR Czechia OR "Czech Republic" OR Denmark OR Estonia OR Finland OR France OR Germany OR Greece OR Hungary OR Ireland OR Italy OR Latvia OR Lithuania OR Luxembourg OR Moldova OR Montenegro OR Netherlands OR Norway OR Poland OR Portugal OR Romania OR Serbia OR Slovakia OR Slovenia OR Spain OR Sweden OR Switzerland OR Ukraine OR "United Kingdom" OR England OR Scotland OR Wales) |

## Picornaviridae

| **Database** | **Full search query** |
| --- | --- |
| PubMed | (Apodemus[Title/Abstract] OR "wood mouse"[Title/Abstract] OR "yellow-necked mouse"[Title/Abstract] OR "striped field mouse"[Title/Abstract]) AND (Picornaviridae[Title/Abstract] OR picornavirus[Title/Abstract] OR "Ljungan virus"[Title/Abstract] OR Ljungan[Title/Abstract] OR LV[Title/Abstract] OR Parechovirus[Title/Abstract] OR "Parechovirus belijungari"[Title/Abstract] OR "encephalomyocarditis virus"[Title/Abstract] OR EMCV[Title/Abstract]) AND (Europe[Title/Abstract] OR European[Title/Abstract] OR Austria[Title/Abstract] OR Belarus[Title/Abstract] OR Belgium[Title/Abstract] OR Bosnia[Title/Abstract] OR Bulgaria[Title/Abstract] OR Croatia[Title/Abstract] OR Czechia[Title/Abstract] OR "Czech Republic"[Title/Abstract] OR Denmark[Title/Abstract] OR Estonia[Title/Abstract] OR Finland[Title/Abstract] OR France[Title/Abstract] OR Germany[Title/Abstract] OR Greece[Title/Abstract] OR Hungary[Title/Abstract] OR Ireland[Title/Abstract] OR Italy[Title/Abstract] OR Latvia[Title/Abstract] OR Lithuania[Title/Abstract] OR Luxembourg[Title/Abstract] OR Moldova[Title/Abstract] OR Montenegro[Title/Abstract] OR Netherlands[Title/Abstract] OR Norway[Title/Abstract] OR Poland[Title/Abstract] OR Portugal[Title/Abstract] OR Romania[Title/Abstract] OR Serbia[Title/Abstract] OR Slovakia[Title/Abstract] OR Slovenia[Title/Abstract] OR Spain[Title/Abstract] OR Sweden[Title/Abstract] OR Switzerland[Title/Abstract] OR Ukraine[Title/Abstract] OR "United Kingdom"[Title/Abstract] OR England[Title/Abstract] OR Scotland[Title/Abstract] OR Wales[Title/Abstract]) |
| Europe PMC | (TITLE_ABS:Apodemus OR TITLE_ABS:"wood mouse" OR TITLE_ABS:"yellow-necked mouse" OR TITLE_ABS:"striped field mouse") AND (TITLE_ABS:Picornaviridae OR TITLE_ABS:picornavirus OR TITLE_ABS:"Ljungan virus" OR TITLE_ABS:Ljungan OR TITLE_ABS:LV OR TITLE_ABS:Parechovirus OR TITLE_ABS:"Parechovirus belijungari" OR TITLE_ABS:"encephalomyocarditis virus" OR TITLE_ABS:EMCV) AND (TITLE_ABS:Europe OR TITLE_ABS:European OR TITLE_ABS:Austria OR TITLE_ABS:Belarus OR TITLE_ABS:Belgium OR TITLE_ABS:Bosnia OR TITLE_ABS:Bulgaria OR TITLE_ABS:Croatia OR TITLE_ABS:Czechia OR TITLE_ABS:"Czech Republic" OR TITLE_ABS:Denmark OR TITLE_ABS:Estonia OR TITLE_ABS:Finland OR TITLE_ABS:France OR TITLE_ABS:Germany OR TITLE_ABS:Greece OR TITLE_ABS:Hungary OR TITLE_ABS:Ireland OR TITLE_ABS:Italy OR TITLE_ABS:Latvia OR TITLE_ABS:Lithuania OR TITLE_ABS:Luxembourg OR TITLE_ABS:Moldova OR TITLE_ABS:Montenegro OR TITLE_ABS:Netherlands OR TITLE_ABS:Norway OR TITLE_ABS:Poland OR TITLE_ABS:Portugal OR TITLE_ABS:Romania OR TITLE_ABS:Serbia OR TITLE_ABS:Slovakia OR TITLE_ABS:Slovenia OR TITLE_ABS:Spain OR TITLE_ABS:Sweden OR TITLE_ABS:Switzerland OR TITLE_ABS:Ukraine OR TITLE_ABS:"United Kingdom" OR TITLE_ABS:England OR TITLE_ABS:Scotland OR TITLE_ABS:Wales) |
| Web of Science | TS=((Apodemus OR "wood mouse" OR "yellow-necked mouse" OR "striped field mouse") AND (Picornaviridae OR picornavirus OR "Ljungan virus" OR Ljungan OR LV OR Parechovirus OR "Parechovirus belijungari" OR "encephalomyocarditis virus" OR EMCV) AND (Europe OR European OR Austria OR Belarus OR Belgium OR Bosnia OR Bulgaria OR Croatia OR Czechia OR "Czech Republic" OR Denmark OR Estonia OR Finland OR France OR Germany OR Greece OR Hungary OR Ireland OR Italy OR Latvia OR Lithuania OR Luxembourg OR Moldova OR Montenegro OR Netherlands OR Norway OR Poland OR Portugal OR Romania OR Serbia OR Slovakia OR Slovenia OR Spain OR Sweden OR Switzerland OR Ukraine OR "United Kingdom" OR England OR Scotland OR Wales)) |
| CABI Digital Library | (Apodemus OR "wood mouse" OR "yellow-necked mouse" OR "striped field mouse") AND (Picornaviridae OR picornavirus OR "Ljungan virus" OR Ljungan OR LV OR Parechovirus OR "Parechovirus belijungari" OR "encephalomyocarditis virus" OR EMCV) AND (Europe OR European OR Austria OR Belarus OR Belgium OR Bosnia OR Bulgaria OR Croatia OR Czechia OR "Czech Republic" OR Denmark OR Estonia OR Finland OR France OR Germany OR Greece OR Hungary OR Ireland OR Italy OR Latvia OR Lithuania OR Luxembourg OR Moldova OR Montenegro OR Netherlands OR Norway OR Poland OR Portugal OR Romania OR Serbia OR Slovakia OR Slovenia OR Spain OR Sweden OR Switzerland OR Ukraine OR "United Kingdom" OR England OR Scotland OR Wales) |

## Poxviridae / cowpox virus

| **Database** | **Full search query** |
| --- | --- |
| PubMed | (Apodemus[Title/Abstract] OR "wood mouse"[Title/Abstract] OR "yellow-necked mouse"[Title/Abstract] OR "striped field mouse"[Title/Abstract]) AND (Poxviridae[Title/Abstract] OR poxvirus[Title/Abstract] OR Orthopoxvirus[Title/Abstract] OR "cowpox virus"[Title/Abstract] OR cowpox[Title/Abstract] OR CPXV[Title/Abstract]) AND (Europe[Title/Abstract] OR European[Title/Abstract] OR Austria[Title/Abstract] OR Belarus[Title/Abstract] OR Belgium[Title/Abstract] OR Bosnia[Title/Abstract] OR Bulgaria[Title/Abstract] OR Croatia[Title/Abstract] OR Czechia[Title/Abstract] OR "Czech Republic"[Title/Abstract] OR Denmark[Title/Abstract] OR Estonia[Title/Abstract] OR Finland[Title/Abstract] OR France[Title/Abstract] OR Germany[Title/Abstract] OR Greece[Title/Abstract] OR Hungary[Title/Abstract] OR Ireland[Title/Abstract] OR Italy[Title/Abstract] OR Latvia[Title/Abstract] OR Lithuania[Title/Abstract] OR Luxembourg[Title/Abstract] OR Moldova[Title/Abstract] OR Montenegro[Title/Abstract] OR Netherlands[Title/Abstract] OR Norway[Title/Abstract] OR Poland[Title/Abstract] OR Portugal[Title/Abstract] OR Romania[Title/Abstract] OR Serbia[Title/Abstract] OR Slovakia[Title/Abstract] OR Slovenia[Title/Abstract] OR Spain[Title/Abstract] OR Sweden[Title/Abstract] OR Switzerland[Title/Abstract] OR Ukraine[Title/Abstract] OR "United Kingdom"[Title/Abstract] OR England[Title/Abstract] OR Scotland[Title/Abstract] OR Wales[Title/Abstract]) |
| Europe PMC | (TITLE_ABS:Apodemus OR TITLE_ABS:"wood mouse" OR TITLE_ABS:"yellow-necked mouse" OR TITLE_ABS:"striped field mouse") AND (TITLE_ABS:Poxviridae OR TITLE_ABS:poxvirus OR TITLE_ABS:Orthopoxvirus OR TITLE_ABS:"cowpox virus" OR TITLE_ABS:cowpox OR TITLE_ABS:CPXV) AND (TITLE_ABS:Europe OR TITLE_ABS:European OR TITLE_ABS:Austria OR TITLE_ABS:Belarus OR TITLE_ABS:Belgium OR TITLE_ABS:Bosnia OR TITLE_ABS:Bulgaria OR TITLE_ABS:Croatia OR TITLE_ABS:Czechia OR TITLE_ABS:"Czech Republic" OR TITLE_ABS:Denmark OR TITLE_ABS:Estonia OR TITLE_ABS:Finland OR TITLE_ABS:France OR TITLE_ABS:Germany OR TITLE_ABS:Greece OR TITLE_ABS:Hungary OR TITLE_ABS:Ireland OR TITLE_ABS:Italy OR TITLE_ABS:Latvia OR TITLE_ABS:Lithuania OR TITLE_ABS:Luxembourg OR TITLE_ABS:Moldova OR TITLE_ABS:Montenegro OR TITLE_ABS:Netherlands OR TITLE_ABS:Norway OR TITLE_ABS:Poland OR TITLE_ABS:Portugal OR TITLE_ABS:Romania OR TITLE_ABS:Serbia OR TITLE_ABS:Slovakia OR TITLE_ABS:Slovenia OR TITLE_ABS:Spain OR TITLE_ABS:Sweden OR TITLE_ABS:Switzerland OR TITLE_ABS:Ukraine OR TITLE_ABS:"United Kingdom" OR TITLE_ABS:England OR TITLE_ABS:Scotland OR TITLE_ABS:Wales) |
| Web of Science | TS=((Apodemus OR "wood mouse" OR "yellow-necked mouse" OR "striped field mouse") AND (Poxviridae OR poxvirus OR Orthopoxvirus OR "cowpox virus" OR cowpox OR CPXV) AND (Europe OR European OR Austria OR Belarus OR Belgium OR Bosnia OR Bulgaria OR Croatia OR Czechia OR "Czech Republic" OR Denmark OR Estonia OR Finland OR France OR Germany OR Greece OR Hungary OR Ireland OR Italy OR Latvia OR Lithuania OR Luxembourg OR Moldova OR Montenegro OR Netherlands OR Norway OR Poland OR Portugal OR Romania OR Serbia OR Slovakia OR Slovenia OR Spain OR Sweden OR Switzerland OR Ukraine OR "United Kingdom" OR England OR Scotland OR Wales)) |
| CABI Digital Library | (Apodemus OR "wood mouse" OR "yellow-necked mouse" OR "striped field mouse") AND (Poxviridae OR poxvirus OR Orthopoxvirus OR "cowpox virus" OR cowpox OR CPXV) AND (Europe OR European OR Austria OR Belarus OR Belgium OR Bosnia OR Bulgaria OR Croatia OR Czechia OR "Czech Republic" OR Denmark OR Estonia OR Finland OR France OR Germany OR Greece OR Hungary OR Ireland OR Italy OR Latvia OR Lithuania OR Luxembourg OR Moldova OR Montenegro OR Netherlands OR Norway OR Poland OR Portugal OR Romania OR Serbia OR Slovakia OR Slovenia OR Spain OR Sweden OR Switzerland OR Ukraine OR "United Kingdom" OR England OR Scotland OR Wales) |

## Francisella tularensis

| **Database** | **Full search query** |
| --- | --- |
| PubMed | (Apodemus[Title/Abstract] OR "wood mouse"[Title/Abstract] OR "yellow-necked mouse"[Title/Abstract] OR "striped field mouse"[Title/Abstract]) AND ("Francisella tularensis"[Title/Abstract] OR "F. tularensis"[Title/Abstract] OR tularemia[Title/Abstract] OR tularaemia[Title/Abstract]) AND (Europe[Title/Abstract] OR European[Title/Abstract] OR Austria[Title/Abstract] OR Belarus[Title/Abstract] OR Belgium[Title/Abstract] OR Bosnia[Title/Abstract] OR Bulgaria[Title/Abstract] OR Croatia[Title/Abstract] OR Czechia[Title/Abstract] OR "Czech Republic"[Title/Abstract] OR Denmark[Title/Abstract] OR Estonia[Title/Abstract] OR Finland[Title/Abstract] OR France[Title/Abstract] OR Germany[Title/Abstract] OR Greece[Title/Abstract] OR Hungary[Title/Abstract] OR Ireland[Title/Abstract] OR Italy[Title/Abstract] OR Latvia[Title/Abstract] OR Lithuania[Title/Abstract] OR Luxembourg[Title/Abstract] OR Moldova[Title/Abstract] OR Montenegro[Title/Abstract] OR Netherlands[Title/Abstract] OR Norway[Title/Abstract] OR Poland[Title/Abstract] OR Portugal[Title/Abstract] OR Romania[Title/Abstract] OR Serbia[Title/Abstract] OR Slovakia[Title/Abstract] OR Slovenia[Title/Abstract] OR Spain[Title/Abstract] OR Sweden[Title/Abstract] OR Switzerland[Title/Abstract] OR Ukraine[Title/Abstract] OR "United Kingdom"[Title/Abstract] OR England[Title/Abstract] OR Scotland[Title/Abstract] OR Wales[Title/Abstract]) |
| Europe PMC | (TITLE_ABS:Apodemus OR TITLE_ABS:"wood mouse" OR TITLE_ABS:"yellow-necked mouse" OR TITLE_ABS:"striped field mouse") AND (TITLE_ABS:"Francisella tularensis" OR TITLE_ABS:"F. tularensis" OR TITLE_ABS:tularemia OR TITLE_ABS:tularaemia) AND (TITLE_ABS:Europe OR TITLE_ABS:European OR TITLE_ABS:Austria OR TITLE_ABS:Belarus OR TITLE_ABS:Belgium OR TITLE_ABS:Bosnia OR TITLE_ABS:Bulgaria OR TITLE_ABS:Croatia OR TITLE_ABS:Czechia OR TITLE_ABS:"Czech Republic" OR TITLE_ABS:Denmark OR TITLE_ABS:Estonia OR TITLE_ABS:Finland OR TITLE_ABS:France OR TITLE_ABS:Germany OR TITLE_ABS:Greece OR TITLE_ABS:Hungary OR TITLE_ABS:Ireland OR TITLE_ABS:Italy OR TITLE_ABS:Latvia OR TITLE_ABS:Lithuania OR TITLE_ABS:Luxembourg OR TITLE_ABS:Moldova OR TITLE_ABS:Montenegro OR TITLE_ABS:Netherlands OR TITLE_ABS:Norway OR TITLE_ABS:Poland OR TITLE_ABS:Portugal OR TITLE_ABS:Romania OR TITLE_ABS:Serbia OR TITLE_ABS:Slovakia OR TITLE_ABS:Slovenia OR TITLE_ABS:Spain OR TITLE_ABS:Sweden OR TITLE_ABS:Switzerland OR TITLE_ABS:Ukraine OR TITLE_ABS:"United Kingdom" OR TITLE_ABS:England OR TITLE_ABS:Scotland OR TITLE_ABS:Wales) |
| Web of Science | TS=((Apodemus OR "wood mouse" OR "yellow-necked mouse" OR "striped field mouse") AND ("Francisella tularensis" OR "F. tularensis" OR tularemia OR tularaemia) AND (Europe OR European OR Austria OR Belarus OR Belgium OR Bosnia OR Bulgaria OR Croatia OR Czechia OR "Czech Republic" OR Denmark OR Estonia OR Finland OR France OR Germany OR Greece OR Hungary OR Ireland OR Italy OR Latvia OR Lithuania OR Luxembourg OR Moldova OR Montenegro OR Netherlands OR Norway OR Poland OR Portugal OR Romania OR Serbia OR Slovakia OR Slovenia OR Spain OR Sweden OR Switzerland OR Ukraine OR "United Kingdom" OR England OR Scotland OR Wales)) |
| CABI Digital Library | (Apodemus OR "wood mouse" OR "yellow-necked mouse" OR "striped field mouse") AND ("Francisella tularensis" OR "F. tularensis" OR tularemia OR tularaemia) AND (Europe OR European OR Austria OR Belarus OR Belgium OR Bosnia OR Bulgaria OR Croatia OR Czechia OR "Czech Republic" OR Denmark OR Estonia OR Finland OR France OR Germany OR Greece OR Hungary OR Ireland OR Italy OR Latvia OR Lithuania OR Luxembourg OR Moldova OR Montenegro OR Netherlands OR Norway OR Poland OR Portugal OR Romania OR Serbia OR Slovakia OR Slovenia OR Spain OR Sweden OR Switzerland OR Ukraine OR "United Kingdom" OR England OR Scotland OR Wales) |

## Leptospira spp.

| **Database** | **Full search query** |
| --- | --- |
| PubMed | (Apodemus[Title/Abstract] OR "wood mouse"[Title/Abstract] OR "yellow-necked mouse"[Title/Abstract] OR "striped field mouse"[Title/Abstract]) AND (Leptospira[Title/Abstract] OR leptospirosis[Title/Abstract] OR "Leptospira interrogans"[Title/Abstract] OR "Leptospira kirschneri"[Title/Abstract] OR "Leptospira borgpetersenii"[Title/Abstract] OR serovar[Title/Abstract] OR serovars[Title/Abstract]) AND (Europe[Title/Abstract] OR European[Title/Abstract] OR Austria[Title/Abstract] OR Belarus[Title/Abstract] OR Belgium[Title/Abstract] OR Bosnia[Title/Abstract] OR Bulgaria[Title/Abstract] OR Croatia[Title/Abstract] OR Czechia[Title/Abstract] OR "Czech Republic"[Title/Abstract] OR Denmark[Title/Abstract] OR Estonia[Title/Abstract] OR Finland[Title/Abstract] OR France[Title/Abstract] OR Germany[Title/Abstract] OR Greece[Title/Abstract] OR Hungary[Title/Abstract] OR Ireland[Title/Abstract] OR Italy[Title/Abstract] OR Latvia[Title/Abstract] OR Lithuania[Title/Abstract] OR Luxembourg[Title/Abstract] OR Moldova[Title/Abstract] OR Montenegro[Title/Abstract] OR Netherlands[Title/Abstract] OR Norway[Title/Abstract] OR Poland[Title/Abstract] OR Portugal[Title/Abstract] OR Romania[Title/Abstract] OR Serbia[Title/Abstract] OR Slovakia[Title/Abstract] OR Slovenia[Title/Abstract] OR Spain[Title/Abstract] OR Sweden[Title/Abstract] OR Switzerland[Title/Abstract] OR Ukraine[Title/Abstract] OR "United Kingdom"[Title/Abstract] OR England[Title/Abstract] OR Scotland[Title/Abstract] OR Wales[Title/Abstract]) |
| Europe PMC | (TITLE_ABS:Apodemus OR TITLE_ABS:"wood mouse" OR TITLE_ABS:"yellow-necked mouse" OR TITLE_ABS:"striped field mouse") AND (TITLE_ABS:Leptospira OR TITLE_ABS:leptospirosis OR TITLE_ABS:"Leptospira interrogans" OR TITLE_ABS:"Leptospira kirschneri" OR TITLE_ABS:"Leptospira borgpetersenii" OR TITLE_ABS:serovar OR TITLE_ABS:serovars) AND (TITLE_ABS:Europe OR TITLE_ABS:European OR TITLE_ABS:Austria OR TITLE_ABS:Belarus OR TITLE_ABS:Belgium OR TITLE_ABS:Bosnia OR TITLE_ABS:Bulgaria OR TITLE_ABS:Croatia OR TITLE_ABS:Czechia OR TITLE_ABS:"Czech Republic" OR TITLE_ABS:Denmark OR TITLE_ABS:Estonia OR TITLE_ABS:Finland OR TITLE_ABS:France OR TITLE_ABS:Germany OR TITLE_ABS:Greece OR TITLE_ABS:Hungary OR TITLE_ABS:Ireland OR TITLE_ABS:Italy OR TITLE_ABS:Latvia OR TITLE_ABS:Lithuania OR TITLE_ABS:Luxembourg OR TITLE_ABS:Moldova OR TITLE_ABS:Montenegro OR TITLE_ABS:Netherlands OR TITLE_ABS:Norway OR TITLE_ABS:Poland OR TITLE_ABS:Portugal OR TITLE_ABS:Romania OR TITLE_ABS:Serbia OR TITLE_ABS:Slovakia OR TITLE_ABS:Slovenia OR TITLE_ABS:Spain OR TITLE_ABS:Sweden OR TITLE_ABS:Switzerland OR TITLE_ABS:Ukraine OR TITLE_ABS:"United Kingdom" OR TITLE_ABS:England OR TITLE_ABS:Scotland OR TITLE_ABS:Wales) |
| Web of Science | TS=((Apodemus OR "wood mouse" OR "yellow-necked mouse" OR "striped field mouse") AND (Leptospira OR leptospirosis OR "Leptospira interrogans" OR "Leptospira kirschneri" OR "Leptospira borgpetersenii" OR serovar OR serovars) AND (Europe OR European OR Austria OR Belarus OR Belgium OR Bosnia OR Bulgaria OR Croatia OR Czechia OR "Czech Republic" OR Denmark OR Estonia OR Finland OR France OR Germany OR Greece OR Hungary OR Ireland OR Italy OR Latvia OR Lithuania OR Luxembourg OR Moldova OR Montenegro OR Netherlands OR Norway OR Poland OR Portugal OR Romania OR Serbia OR Slovakia OR Slovenia OR Spain OR Sweden OR Switzerland OR Ukraine OR "United Kingdom" OR England OR Scotland OR Wales)) |
| CABI Digital Library | (Apodemus OR "wood mouse" OR "yellow-necked mouse" OR "striped field mouse") AND (Leptospira OR leptospirosis OR "Leptospira interrogans" OR "Leptospira kirschneri" OR "Leptospira borgpetersenii" OR serovar OR serovars) AND (Europe OR European OR Austria OR Belarus OR Belgium OR Bosnia OR Bulgaria OR Croatia OR Czechia OR "Czech Republic" OR Denmark OR Estonia OR Finland OR France OR Germany OR Greece OR Hungary OR Ireland OR Italy OR Latvia OR Lithuania OR Luxembourg OR Moldova OR Montenegro OR Netherlands OR Norway OR Poland OR Portugal OR Romania OR Serbia OR Slovakia OR Slovenia OR Spain OR Sweden OR Switzerland OR Ukraine OR "United Kingdom" OR England OR Scotland OR Wales) |

## Yersinia spp.

| **Database** | **Full search query** |
| --- | --- |
| PubMed | (Apodemus[Title/Abstract] OR "wood mouse"[Title/Abstract] OR "yellow-necked mouse"[Title/Abstract] OR "striped field mouse"[Title/Abstract]) AND (Yersinia[Title/Abstract] OR "Yersinia enterocolitica"[Title/Abstract] OR "Yersinia pseudotuberculosis"[Title/Abstract] OR "Yersinia pestis"[Title/Abstract] OR yersiniosis[Title/Abstract] OR plague[Title/Abstract]) AND (Europe[Title/Abstract] OR European[Title/Abstract] OR Austria[Title/Abstract] OR Belarus[Title/Abstract] OR Belgium[Title/Abstract] OR Bosnia[Title/Abstract] OR Bulgaria[Title/Abstract] OR Croatia[Title/Abstract] OR Czechia[Title/Abstract] OR "Czech Republic"[Title/Abstract] OR Denmark[Title/Abstract] OR Estonia[Title/Abstract] OR Finland[Title/Abstract] OR France[Title/Abstract] OR Germany[Title/Abstract] OR Greece[Title/Abstract] OR Hungary[Title/Abstract] OR Ireland[Title/Abstract] OR Italy[Title/Abstract] OR Latvia[Title/Abstract] OR Lithuania[Title/Abstract] OR Luxembourg[Title/Abstract] OR Moldova[Title/Abstract] OR Montenegro[Title/Abstract] OR Netherlands[Title/Abstract] OR Norway[Title/Abstract] OR Poland[Title/Abstract] OR Portugal[Title/Abstract] OR Romania[Title/Abstract] OR Serbia[Title/Abstract] OR Slovakia[Title/Abstract] OR Slovenia[Title/Abstract] OR Spain[Title/Abstract] OR Sweden[Title/Abstract] OR Switzerland[Title/Abstract] OR Ukraine[Title/Abstract] OR "United Kingdom"[Title/Abstract] OR England[Title/Abstract] OR Scotland[Title/Abstract] OR Wales[Title/Abstract]) |
| Europe PMC | (TITLE_ABS:Apodemus OR TITLE_ABS:"wood mouse" OR TITLE_ABS:"yellow-necked mouse" OR TITLE_ABS:"striped field mouse") AND (TITLE_ABS:Yersinia OR TITLE_ABS:"Yersinia enterocolitica" OR TITLE_ABS:"Yersinia pseudotuberculosis" OR TITLE_ABS:"Yersinia pestis" OR TITLE_ABS:yersiniosis OR TITLE_ABS:plague) AND (TITLE_ABS:Europe OR TITLE_ABS:European OR TITLE_ABS:Austria OR TITLE_ABS:Belarus OR TITLE_ABS:Belgium OR TITLE_ABS:Bosnia OR TITLE_ABS:Bulgaria OR TITLE_ABS:Croatia OR TITLE_ABS:Czechia OR TITLE_ABS:"Czech Republic" OR TITLE_ABS:Denmark OR TITLE_ABS:Estonia OR TITLE_ABS:Finland OR TITLE_ABS:France OR TITLE_ABS:Germany OR TITLE_ABS:Greece OR TITLE_ABS:Hungary OR TITLE_ABS:Ireland OR TITLE_ABS:Italy OR TITLE_ABS:Latvia OR TITLE_ABS:Lithuania OR TITLE_ABS:Luxembourg OR TITLE_ABS:Moldova OR TITLE_ABS:Montenegro OR TITLE_ABS:Netherlands OR TITLE_ABS:Norway OR TITLE_ABS:Poland OR TITLE_ABS:Portugal OR TITLE_ABS:Romania OR TITLE_ABS:Serbia OR TITLE_ABS:Slovakia OR TITLE_ABS:Slovenia OR TITLE_ABS:Spain OR TITLE_ABS:Sweden OR TITLE_ABS:Switzerland OR TITLE_ABS:Ukraine OR TITLE_ABS:"United Kingdom" OR TITLE_ABS:England OR TITLE_ABS:Scotland OR TITLE_ABS:Wales) |
| Web of Science | TS=((Apodemus OR "wood mouse" OR "yellow-necked mouse" OR "striped field mouse") AND (Yersinia OR "Yersinia enterocolitica" OR "Yersinia pseudotuberculosis" OR "Yersinia pestis" OR yersiniosis OR plague) AND (Europe OR European OR Austria OR Belarus OR Belgium OR Bosnia OR Bulgaria OR Croatia OR Czechia OR "Czech Republic" OR Denmark OR Estonia OR Finland OR France OR Germany OR Greece OR Hungary OR Ireland OR Italy OR Latvia OR Lithuania OR Luxembourg OR Moldova OR Montenegro OR Netherlands OR Norway OR Poland OR Portugal OR Romania OR Serbia OR Slovakia OR Slovenia OR Spain OR Sweden OR Switzerland OR Ukraine OR "United Kingdom" OR England OR Scotland OR Wales)) |
| CABI Digital Library | (Apodemus OR "wood mouse" OR "yellow-necked mouse" OR "striped field mouse") AND (Yersinia OR "Yersinia enterocolitica" OR "Yersinia pseudotuberculosis" OR "Yersinia pestis" OR yersiniosis OR plague) AND (Europe OR European OR Austria OR Belarus OR Belgium OR Bosnia OR Bulgaria OR Croatia OR Czechia OR "Czech Republic" OR Denmark OR Estonia OR Finland OR France OR Germany OR Greece OR Hungary OR Ireland OR Italy OR Latvia OR Lithuania OR Luxembourg OR Moldova OR Montenegro OR Netherlands OR Norway OR Poland OR Portugal OR Romania OR Serbia OR Slovakia OR Slovenia OR Spain OR Sweden OR Switzerland OR Ukraine OR "United Kingdom" OR England OR Scotland OR Wales) |

## Astroviridae

| **Database** | **Full search query** |
| --- | --- |
| PubMed | (Apodemus[Title/Abstract] OR "wood mouse"[Title/Abstract] OR "yellow-necked mouse"[Title/Abstract] OR "striped field mouse"[Title/Abstract]) AND (Astroviridae[Title/Abstract] OR astrovirus[Title/Abstract] OR astroviruses[Title/Abstract] OR Mamastrovirus[Title/Abstract]) AND (Europe[Title/Abstract] OR European[Title/Abstract] OR Austria[Title/Abstract] OR Belarus[Title/Abstract] OR Belgium[Title/Abstract] OR Bosnia[Title/Abstract] OR Bulgaria[Title/Abstract] OR Croatia[Title/Abstract] OR Czechia[Title/Abstract] OR "Czech Republic"[Title/Abstract] OR Denmark[Title/Abstract] OR Estonia[Title/Abstract] OR Finland[Title/Abstract] OR France[Title/Abstract] OR Germany[Title/Abstract] OR Greece[Title/Abstract] OR Hungary[Title/Abstract] OR Ireland[Title/Abstract] OR Italy[Title/Abstract] OR Latvia[Title/Abstract] OR Lithuania[Title/Abstract] OR Luxembourg[Title/Abstract] OR Moldova[Title/Abstract] OR Montenegro[Title/Abstract] OR Netherlands[Title/Abstract] OR Norway[Title/Abstract] OR Poland[Title/Abstract] OR Portugal[Title/Abstract] OR Romania[Title/Abstract] OR Serbia[Title/Abstract] OR Slovakia[Title/Abstract] OR Slovenia[Title/Abstract] OR Spain[Title/Abstract] OR Sweden[Title/Abstract] OR Switzerland[Title/Abstract] OR Ukraine[Title/Abstract] OR "United Kingdom"[Title/Abstract] OR England[Title/Abstract] OR Scotland[Title/Abstract] OR Wales[Title/Abstract]) |
| Europe PMC | (TITLE_ABS:Apodemus OR TITLE_ABS:"wood mouse" OR TITLE_ABS:"yellow-necked mouse" OR TITLE_ABS:"striped field mouse") AND (TITLE_ABS:Astroviridae OR TITLE_ABS:astrovirus OR TITLE_ABS:astroviruses OR TITLE_ABS:Mamastrovirus) AND (TITLE_ABS:Europe OR TITLE_ABS:European OR TITLE_ABS:Austria OR TITLE_ABS:Belarus OR TITLE_ABS:Belgium OR TITLE_ABS:Bosnia OR TITLE_ABS:Bulgaria OR TITLE_ABS:Croatia OR TITLE_ABS:Czechia OR TITLE_ABS:"Czech Republic" OR TITLE_ABS:Denmark OR TITLE_ABS:Estonia OR TITLE_ABS:Finland OR TITLE_ABS:France OR TITLE_ABS:Germany OR TITLE_ABS:Greece OR TITLE_ABS:Hungary OR TITLE_ABS:Ireland OR TITLE_ABS:Italy OR TITLE_ABS:Latvia OR TITLE_ABS:Lithuania OR TITLE_ABS:Luxembourg OR TITLE_ABS:Moldova OR TITLE_ABS:Montenegro OR TITLE_ABS:Netherlands OR TITLE_ABS:Norway OR TITLE_ABS:Poland OR TITLE_ABS:Portugal OR TITLE_ABS:Romania OR TITLE_ABS:Serbia OR TITLE_ABS:Slovakia OR TITLE_ABS:Slovenia OR TITLE_ABS:Spain OR TITLE_ABS:Sweden OR TITLE_ABS:Switzerland OR TITLE_ABS:Ukraine OR TITLE_ABS:"United Kingdom" OR TITLE_ABS:England OR TITLE_ABS:Scotland OR TITLE_ABS:Wales) |
| Web of Science | TS=((Apodemus OR "wood mouse" OR "yellow-necked mouse" OR "striped field mouse") AND (Astroviridae OR astrovirus OR astroviruses OR Mamastrovirus) AND (Europe OR European OR Austria OR Belarus OR Belgium OR Bosnia OR Bulgaria OR Croatia OR Czechia OR "Czech Republic" OR Denmark OR Estonia OR Finland OR France OR Germany OR Greece OR Hungary OR Ireland OR Italy OR Latvia OR Lithuania OR Luxembourg OR Moldova OR Montenegro OR Netherlands OR Norway OR Poland OR Portugal OR Romania OR Serbia OR Slovakia OR Slovenia OR Spain OR Sweden OR Switzerland OR Ukraine OR "United Kingdom" OR England OR Scotland OR Wales)) |
| CABI Digital Library | (Apodemus OR "wood mouse" OR "yellow-necked mouse" OR "striped field mouse") AND (Astroviridae OR astrovirus OR astroviruses OR Mamastrovirus) AND (Europe OR European OR Austria OR Belarus OR Belgium OR Bosnia OR Bulgaria OR Croatia OR Czechia OR "Czech Republic" OR Denmark OR Estonia OR Finland OR France OR Germany OR Greece OR Hungary OR Ireland OR Italy OR Latvia OR Lithuania OR Luxembourg OR Moldova OR Montenegro OR Netherlands OR Norway OR Poland OR Portugal OR Romania OR Serbia OR Slovakia OR Slovenia OR Spain OR Sweden OR Switzerland OR Ukraine OR "United Kingdom" OR England OR Scotland OR Wales) |

## Coronaviridae

| **Database** | **Full search query** |
| --- | --- |
| PubMed | (Apodemus[Title/Abstract] OR "wood mouse"[Title/Abstract] OR "yellow-necked mouse"[Title/Abstract] OR "striped field mouse"[Title/Abstract]) AND (Coronaviridae[Title/Abstract] OR coronavirus[Title/Abstract] OR coronaviruses[Title/Abstract] OR Alphacoronavirus[Title/Abstract] OR Betacoronavirus[Title/Abstract] OR "SARS-CoV-2"[Title/Abstract] OR "Betacoronavirus muris"[Title/Abstract]) AND (Europe[Title/Abstract] OR European[Title/Abstract] OR Austria[Title/Abstract] OR Belarus[Title/Abstract] OR Belgium[Title/Abstract] OR Bosnia[Title/Abstract] OR Bulgaria[Title/Abstract] OR Croatia[Title/Abstract] OR Czechia[Title/Abstract] OR "Czech Republic"[Title/Abstract] OR Denmark[Title/Abstract] OR Estonia[Title/Abstract] OR Finland[Title/Abstract] OR France[Title/Abstract] OR Germany[Title/Abstract] OR Greece[Title/Abstract] OR Hungary[Title/Abstract] OR Ireland[Title/Abstract] OR Italy[Title/Abstract] OR Latvia[Title/Abstract] OR Lithuania[Title/Abstract] OR Luxembourg[Title/Abstract] OR Moldova[Title/Abstract] OR Montenegro[Title/Abstract] OR Netherlands[Title/Abstract] OR Norway[Title/Abstract] OR Poland[Title/Abstract] OR Portugal[Title/Abstract] OR Romania[Title/Abstract] OR Serbia[Title/Abstract] OR Slovakia[Title/Abstract] OR Slovenia[Title/Abstract] OR Spain[Title/Abstract] OR Sweden[Title/Abstract] OR Switzerland[Title/Abstract] OR Ukraine[Title/Abstract] OR "United Kingdom"[Title/Abstract] OR England[Title/Abstract] OR Scotland[Title/Abstract] OR Wales[Title/Abstract]) |
| Europe PMC | (TITLE_ABS:Apodemus OR TITLE_ABS:"wood mouse" OR TITLE_ABS:"yellow-necked mouse" OR TITLE_ABS:"striped field mouse") AND (TITLE_ABS:Coronaviridae OR TITLE_ABS:coronavirus OR TITLE_ABS:coronaviruses OR TITLE_ABS:Alphacoronavirus OR TITLE_ABS:Betacoronavirus OR TITLE_ABS:"SARS-CoV-2" OR TITLE_ABS:"Betacoronavirus muris") AND (TITLE_ABS:Europe OR TITLE_ABS:European OR TITLE_ABS:Austria OR TITLE_ABS:Belarus OR TITLE_ABS:Belgium OR TITLE_ABS:Bosnia OR TITLE_ABS:Bulgaria OR TITLE_ABS:Croatia OR TITLE_ABS:Czechia OR TITLE_ABS:"Czech Republic" OR TITLE_ABS:Denmark OR TITLE_ABS:Estonia OR TITLE_ABS:Finland OR TITLE_ABS:France OR TITLE_ABS:Germany OR TITLE_ABS:Greece OR TITLE_ABS:Hungary OR TITLE_ABS:Ireland OR TITLE_ABS:Italy OR TITLE_ABS:Latvia OR TITLE_ABS:Lithuania OR TITLE_ABS:Luxembourg OR TITLE_ABS:Moldova OR TITLE_ABS:Montenegro OR TITLE_ABS:Netherlands OR TITLE_ABS:Norway OR TITLE_ABS:Poland OR TITLE_ABS:Portugal OR TITLE_ABS:Romania OR TITLE_ABS:Serbia OR TITLE_ABS:Slovakia OR TITLE_ABS:Slovenia OR TITLE_ABS:Spain OR TITLE_ABS:Sweden OR TITLE_ABS:Switzerland OR TITLE_ABS:Ukraine OR TITLE_ABS:"United Kingdom" OR TITLE_ABS:England OR TITLE_ABS:Scotland OR TITLE_ABS:Wales) |
| Web of Science | TS=((Apodemus OR "wood mouse" OR "yellow-necked mouse" OR "striped field mouse") AND (Coronaviridae OR coronavirus OR coronaviruses OR Alphacoronavirus OR Betacoronavirus OR "SARS-CoV-2" OR "Betacoronavirus muris") AND (Europe OR European OR Austria OR Belarus OR Belgium OR Bosnia OR Bulgaria OR Croatia OR Czechia OR "Czech Republic" OR Denmark OR Estonia OR Finland OR France OR Germany OR Greece OR Hungary OR Ireland OR Italy OR Latvia OR Lithuania OR Luxembourg OR Moldova OR Montenegro OR Netherlands OR Norway OR Poland OR Portugal OR Romania OR Serbia OR Slovakia OR Slovenia OR Spain OR Sweden OR Switzerland OR Ukraine OR "United Kingdom" OR England OR Scotland OR Wales)) |
| CABI Digital Library | (Apodemus OR "wood mouse" OR "yellow-necked mouse" OR "striped field mouse") AND (Coronaviridae OR coronavirus OR coronaviruses OR Alphacoronavirus OR Betacoronavirus OR "SARS-CoV-2" OR "Betacoronavirus muris") AND (Europe OR European OR Austria OR Belarus OR Belgium OR Bosnia OR Bulgaria OR Croatia OR Czechia OR "Czech Republic" OR Denmark OR Estonia OR Finland OR France OR Germany OR Greece OR Hungary OR Ireland OR Italy OR Latvia OR Lithuania OR Luxembourg OR Moldova OR Montenegro OR Netherlands OR Norway OR Poland OR Portugal OR Romania OR Serbia OR Slovakia OR Slovenia OR Spain OR Sweden OR Switzerland OR Ukraine OR "United Kingdom" OR England OR Scotland OR Wales) |

## Paramyxoviridae

| **Database** | **Full search query** |
| --- | --- |
| PubMed | (Apodemus[Title/Abstract] OR "wood mouse"[Title/Abstract] OR "yellow-necked mouse"[Title/Abstract] OR "striped field mouse"[Title/Abstract]) AND (Paramyxoviridae[Title/Abstract] OR paramyxovirus[Title/Abstract] OR paramyxoviruses[Title/Abstract] OR Morbillivirus[Title/Abstract] OR Respirovirus[Title/Abstract] OR "Sendai virus"[Title/Abstract]) AND (Europe[Title/Abstract] OR European[Title/Abstract] OR Austria[Title/Abstract] OR Belarus[Title/Abstract] OR Belgium[Title/Abstract] OR Bosnia[Title/Abstract] OR Bulgaria[Title/Abstract] OR Croatia[Title/Abstract] OR Czechia[Title/Abstract] OR "Czech Republic"[Title/Abstract] OR Denmark[Title/Abstract] OR Estonia[Title/Abstract] OR Finland[Title/Abstract] OR France[Title/Abstract] OR Germany[Title/Abstract] OR Greece[Title/Abstract] OR Hungary[Title/Abstract] OR Ireland[Title/Abstract] OR Italy[Title/Abstract] OR Latvia[Title/Abstract] OR Lithuania[Title/Abstract] OR Luxembourg[Title/Abstract] OR Moldova[Title/Abstract] OR Montenegro[Title/Abstract] OR Netherlands[Title/Abstract] OR Norway[Title/Abstract] OR Poland[Title/Abstract] OR Portugal[Title/Abstract] OR Romania[Title/Abstract] OR Serbia[Title/Abstract] OR Slovakia[Title/Abstract] OR Slovenia[Title/Abstract] OR Spain[Title/Abstract] OR Sweden[Title/Abstract] OR Switzerland[Title/Abstract] OR Ukraine[Title/Abstract] OR "United Kingdom"[Title/Abstract] OR England[Title/Abstract] OR Scotland[Title/Abstract] OR Wales[Title/Abstract]) |
| Europe PMC | (TITLE_ABS:Apodemus OR TITLE_ABS:"wood mouse" OR TITLE_ABS:"yellow-necked mouse" OR TITLE_ABS:"striped field mouse") AND (TITLE_ABS:Paramyxoviridae OR TITLE_ABS:paramyxovirus OR TITLE_ABS:paramyxoviruses OR TITLE_ABS:Morbillivirus OR TITLE_ABS:Respirovirus OR TITLE_ABS:"Sendai virus") AND (TITLE_ABS:Europe OR TITLE_ABS:European OR TITLE_ABS:Austria OR TITLE_ABS:Belarus OR TITLE_ABS:Belgium OR TITLE_ABS:Bosnia OR TITLE_ABS:Bulgaria OR TITLE_ABS:Croatia OR TITLE_ABS:Czechia OR TITLE_ABS:"Czech Republic" OR TITLE_ABS:Denmark OR TITLE_ABS:Estonia OR TITLE_ABS:Finland OR TITLE_ABS:France OR TITLE_ABS:Germany OR TITLE_ABS:Greece OR TITLE_ABS:Hungary OR TITLE_ABS:Ireland OR TITLE_ABS:Italy OR TITLE_ABS:Latvia OR TITLE_ABS:Lithuania OR TITLE_ABS:Luxembourg OR TITLE_ABS:Moldova OR TITLE_ABS:Montenegro OR TITLE_ABS:Netherlands OR TITLE_ABS:Norway OR TITLE_ABS:Poland OR TITLE_ABS:Portugal OR TITLE_ABS:Romania OR TITLE_ABS:Serbia OR TITLE_ABS:Slovakia OR TITLE_ABS:Slovenia OR TITLE_ABS:Spain OR TITLE_ABS:Sweden OR TITLE_ABS:Switzerland OR TITLE_ABS:Ukraine OR TITLE_ABS:"United Kingdom" OR TITLE_ABS:England OR TITLE_ABS:Scotland OR TITLE_ABS:Wales) |
| Web of Science | TS=((Apodemus OR "wood mouse" OR "yellow-necked mouse" OR "striped field mouse") AND (Paramyxoviridae OR paramyxovirus OR paramyxoviruses OR Morbillivirus OR Respirovirus OR "Sendai virus") AND (Europe OR European OR Austria OR Belarus OR Belgium OR Bosnia OR Bulgaria OR Croatia OR Czechia OR "Czech Republic" OR Denmark OR Estonia OR Finland OR France OR Germany OR Greece OR Hungary OR Ireland OR Italy OR Latvia OR Lithuania OR Luxembourg OR Moldova OR Montenegro OR Netherlands OR Norway OR Poland OR Portugal OR Romania OR Serbia OR Slovakia OR Slovenia OR Spain OR Sweden OR Switzerland OR Ukraine OR "United Kingdom" OR England OR Scotland OR Wales)) |
| CABI Digital Library | (Apodemus OR "wood mouse" OR "yellow-necked mouse" OR "striped field mouse") AND (Paramyxoviridae OR paramyxovirus OR paramyxoviruses OR Morbillivirus OR Respirovirus OR "Sendai virus") AND (Europe OR European OR Austria OR Belarus OR Belgium OR Bosnia OR Bulgaria OR Croatia OR Czechia OR "Czech Republic" OR Denmark OR Estonia OR Finland OR France OR Germany OR Greece OR Hungary OR Ireland OR Italy OR Latvia OR Lithuania OR Luxembourg OR Moldova OR Montenegro OR Netherlands OR Norway OR Poland OR Portugal OR Romania OR Serbia OR Slovakia OR Slovenia OR Spain OR Sweden OR Switzerland OR Ukraine OR "United Kingdom" OR England OR Scotland OR Wales) |

## Campylobacter spp.

| **Database** | **Full search query** |
| --- | --- |
| PubMed | (Apodemus[Title/Abstract] OR "wood mouse"[Title/Abstract] OR "yellow-necked mouse"[Title/Abstract] OR "striped field mouse"[Title/Abstract]) AND (Campylobacter[Title/Abstract] OR "Campylobacter jejuni"[Title/Abstract] OR "Campylobacter coli"[Title/Abstract] OR "Campylobacter upsaliensis"[Title/Abstract] OR "C. jejuni"[Title/Abstract] OR "C. coli"[Title/Abstract] OR "C. upsaliensis"[Title/Abstract]) AND (Europe[Title/Abstract] OR European[Title/Abstract] OR Austria[Title/Abstract] OR Belarus[Title/Abstract] OR Belgium[Title/Abstract] OR Bosnia[Title/Abstract] OR Bulgaria[Title/Abstract] OR Croatia[Title/Abstract] OR Czechia[Title/Abstract] OR "Czech Republic"[Title/Abstract] OR Denmark[Title/Abstract] OR Estonia[Title/Abstract] OR Finland[Title/Abstract] OR France[Title/Abstract] OR Germany[Title/Abstract] OR Greece[Title/Abstract] OR Hungary[Title/Abstract] OR Ireland[Title/Abstract] OR Italy[Title/Abstract] OR Latvia[Title/Abstract] OR Lithuania[Title/Abstract] OR Luxembourg[Title/Abstract] OR Moldova[Title/Abstract] OR Montenegro[Title/Abstract] OR Netherlands[Title/Abstract] OR Norway[Title/Abstract] OR Poland[Title/Abstract] OR Portugal[Title/Abstract] OR Romania[Title/Abstract] OR Serbia[Title/Abstract] OR Slovakia[Title/Abstract] OR Slovenia[Title/Abstract] OR Spain[Title/Abstract] OR Sweden[Title/Abstract] OR Switzerland[Title/Abstract] OR Ukraine[Title/Abstract] OR "United Kingdom"[Title/Abstract] OR England[Title/Abstract] OR Scotland[Title/Abstract] OR Wales[Title/Abstract]) |
| Europe PMC | (TITLE_ABS:Apodemus OR TITLE_ABS:"wood mouse" OR TITLE_ABS:"yellow-necked mouse" OR TITLE_ABS:"striped field mouse") AND (TITLE_ABS:Campylobacter OR TITLE_ABS:"Campylobacter jejuni" OR TITLE_ABS:"Campylobacter coli" OR TITLE_ABS:"Campylobacter upsaliensis" OR TITLE_ABS:"C. jejuni" OR TITLE_ABS:"C. coli" OR TITLE_ABS:"C. upsaliensis") AND (TITLE_ABS:Europe OR TITLE_ABS:European OR TITLE_ABS:Austria OR TITLE_ABS:Belarus OR TITLE_ABS:Belgium OR TITLE_ABS:Bosnia OR TITLE_ABS:Bulgaria OR TITLE_ABS:Croatia OR TITLE_ABS:Czechia OR TITLE_ABS:"Czech Republic" OR TITLE_ABS:Denmark OR TITLE_ABS:Estonia OR TITLE_ABS:Finland OR TITLE_ABS:France OR TITLE_ABS:Germany OR TITLE_ABS:Greece OR TITLE_ABS:Hungary OR TITLE_ABS:Ireland OR TITLE_ABS:Italy OR TITLE_ABS:Latvia OR TITLE_ABS:Lithuania OR TITLE_ABS:Luxembourg OR TITLE_ABS:Moldova OR TITLE_ABS:Montenegro OR TITLE_ABS:Netherlands OR TITLE_ABS:Norway OR TITLE_ABS:Poland OR TITLE_ABS:Portugal OR TITLE_ABS:Romania OR TITLE_ABS:Serbia OR TITLE_ABS:Slovakia OR TITLE_ABS:Slovenia OR TITLE_ABS:Spain OR TITLE_ABS:Sweden OR TITLE_ABS:Switzerland OR TITLE_ABS:Ukraine OR TITLE_ABS:"United Kingdom" OR TITLE_ABS:England OR TITLE_ABS:Scotland OR TITLE_ABS:Wales) |
| Web of Science | TS=((Apodemus OR "wood mouse" OR "yellow-necked mouse" OR "striped field mouse") AND (Campylobacter OR "Campylobacter jejuni" OR "Campylobacter coli" OR "Campylobacter upsaliensis" OR "C. jejuni" OR "C. coli" OR "C. upsaliensis") AND (Europe OR European OR Austria OR Belarus OR Belgium OR Bosnia OR Bulgaria OR Croatia OR Czechia OR "Czech Republic" OR Denmark OR Estonia OR Finland OR France OR Germany OR Greece OR Hungary OR Ireland OR Italy OR Latvia OR Lithuania OR Luxembourg OR Moldova OR Montenegro OR Netherlands OR Norway OR Poland OR Portugal OR Romania OR Serbia OR Slovakia OR Slovenia OR Spain OR Sweden OR Switzerland OR Ukraine OR "United Kingdom" OR England OR Scotland OR Wales)) |
| CABI Digital Library | (Apodemus OR "wood mouse" OR "yellow-necked mouse" OR "striped field mouse") AND (Campylobacter OR "Campylobacter jejuni" OR "Campylobacter coli" OR "Campylobacter upsaliensis" OR "C. jejuni" OR "C. coli" OR "C. upsaliensis") AND (Europe OR European OR Austria OR Belarus OR Belgium OR Bosnia OR Bulgaria OR Croatia OR Czechia OR "Czech Republic" OR Denmark OR Estonia OR Finland OR France OR Germany OR Greece OR Hungary OR Ireland OR Italy OR Latvia OR Lithuania OR Luxembourg OR Moldova OR Montenegro OR Netherlands OR Norway OR Poland OR Portugal OR Romania OR Serbia OR Slovakia OR Slovenia OR Spain OR Sweden OR Switzerland OR Ukraine OR "United Kingdom" OR England OR Scotland OR Wales) |

## Clostridioides difficile

| **Database** | **Full search query** |
| --- | --- |
| PubMed | (Apodemus[Title/Abstract] OR "wood mouse"[Title/Abstract] OR "yellow-necked mouse"[Title/Abstract] OR "striped field mouse"[Title/Abstract]) AND ("Clostridioides difficile"[Title/Abstract] OR "Clostridium difficile"[Title/Abstract] OR "C. difficile"[Title/Abstract]) AND (Europe[Title/Abstract] OR European[Title/Abstract] OR Austria[Title/Abstract] OR Belarus[Title/Abstract] OR Belgium[Title/Abstract] OR Bosnia[Title/Abstract] OR Bulgaria[Title/Abstract] OR Croatia[Title/Abstract] OR Czechia[Title/Abstract] OR "Czech Republic"[Title/Abstract] OR Denmark[Title/Abstract] OR Estonia[Title/Abstract] OR Finland[Title/Abstract] OR France[Title/Abstract] OR Germany[Title/Abstract] OR Greece[Title/Abstract] OR Hungary[Title/Abstract] OR Ireland[Title/Abstract] OR Italy[Title/Abstract] OR Latvia[Title/Abstract] OR Lithuania[Title/Abstract] OR Luxembourg[Title/Abstract] OR Moldova[Title/Abstract] OR Montenegro[Title/Abstract] OR Netherlands[Title/Abstract] OR Norway[Title/Abstract] OR Poland[Title/Abstract] OR Portugal[Title/Abstract] OR Romania[Title/Abstract] OR Serbia[Title/Abstract] OR Slovakia[Title/Abstract] OR Slovenia[Title/Abstract] OR Spain[Title/Abstract] OR Sweden[Title/Abstract] OR Switzerland[Title/Abstract] OR Ukraine[Title/Abstract] OR "United Kingdom"[Title/Abstract] OR England[Title/Abstract] OR Scotland[Title/Abstract] OR Wales[Title/Abstract]) |
| Europe PMC | (TITLE_ABS:Apodemus OR TITLE_ABS:"wood mouse" OR TITLE_ABS:"yellow-necked mouse" OR TITLE_ABS:"striped field mouse") AND (TITLE_ABS:"Clostridioides difficile" OR TITLE_ABS:"Clostridium difficile" OR TITLE_ABS:"C. difficile") AND (TITLE_ABS:Europe OR TITLE_ABS:European OR TITLE_ABS:Austria OR TITLE_ABS:Belarus OR TITLE_ABS:Belgium OR TITLE_ABS:Bosnia OR TITLE_ABS:Bulgaria OR TITLE_ABS:Croatia OR TITLE_ABS:Czechia OR TITLE_ABS:"Czech Republic" OR TITLE_ABS:Denmark OR TITLE_ABS:Estonia OR TITLE_ABS:Finland OR TITLE_ABS:France OR TITLE_ABS:Germany OR TITLE_ABS:Greece OR TITLE_ABS:Hungary OR TITLE_ABS:Ireland OR TITLE_ABS:Italy OR TITLE_ABS:Latvia OR TITLE_ABS:Lithuania OR TITLE_ABS:Luxembourg OR TITLE_ABS:Moldova OR TITLE_ABS:Montenegro OR TITLE_ABS:Netherlands OR TITLE_ABS:Norway OR TITLE_ABS:Poland OR TITLE_ABS:Portugal OR TITLE_ABS:Romania OR TITLE_ABS:Serbia OR TITLE_ABS:Slovakia OR TITLE_ABS:Slovenia OR TITLE_ABS:Spain OR TITLE_ABS:Sweden OR TITLE_ABS:Switzerland OR TITLE_ABS:Ukraine OR TITLE_ABS:"United Kingdom" OR TITLE_ABS:England OR TITLE_ABS:Scotland OR TITLE_ABS:Wales) |
| Web of Science | TS=((Apodemus OR "wood mouse" OR "yellow-necked mouse" OR "striped field mouse") AND ("Clostridioides difficile" OR "Clostridium difficile" OR "C. difficile") AND (Europe OR European OR Austria OR Belarus OR Belgium OR Bosnia OR Bulgaria OR Croatia OR Czechia OR "Czech Republic" OR Denmark OR Estonia OR Finland OR France OR Germany OR Greece OR Hungary OR Ireland OR Italy OR Latvia OR Lithuania OR Luxembourg OR Moldova OR Montenegro OR Netherlands OR Norway OR Poland OR Portugal OR Romania OR Serbia OR Slovakia OR Slovenia OR Spain OR Sweden OR Switzerland OR Ukraine OR "United Kingdom" OR England OR Scotland OR Wales)) |
| CABI Digital Library | (Apodemus OR "wood mouse" OR "yellow-necked mouse" OR "striped field mouse") AND ("Clostridioides difficile" OR "Clostridium difficile" OR "C. difficile") AND (Europe OR European OR Austria OR Belarus OR Belgium OR Bosnia OR Bulgaria OR Croatia OR Czechia OR "Czech Republic" OR Denmark OR Estonia OR Finland OR France OR Germany OR Greece OR Hungary OR Ireland OR Italy OR Latvia OR Lithuania OR Luxembourg OR Moldova OR Montenegro OR Netherlands OR Norway OR Poland OR Portugal OR Romania OR Serbia OR Slovakia OR Slovenia OR Spain OR Sweden OR Switzerland OR Ukraine OR "United Kingdom" OR England OR Scotland OR Wales) |

## Escherichia coli

| **Database** | **Full search query** |
| --- | --- |
| PubMed | (Apodemus[Title/Abstract] OR "wood mouse"[Title/Abstract] OR "yellow-necked mouse"[Title/Abstract] OR "striped field mouse"[Title/Abstract]) AND ("Escherichia coli"[Title/Abstract] OR "E. coli"[Title/Abstract] OR "antimicrobial resistance"[Title/Abstract] OR AMR[Title/Abstract] OR ESBL[Title/Abstract] OR "extended-spectrum beta-lactamase"[Title/Abstract]) AND (Europe[Title/Abstract] OR European[Title/Abstract] OR Austria[Title/Abstract] OR Belarus[Title/Abstract] OR Belgium[Title/Abstract] OR Bosnia[Title/Abstract] OR Bulgaria[Title/Abstract] OR Croatia[Title/Abstract] OR Czechia[Title/Abstract] OR "Czech Republic"[Title/Abstract] OR Denmark[Title/Abstract] OR Estonia[Title/Abstract] OR Finland[Title/Abstract] OR France[Title/Abstract] OR Germany[Title/Abstract] OR Greece[Title/Abstract] OR Hungary[Title/Abstract] OR Ireland[Title/Abstract] OR Italy[Title/Abstract] OR Latvia[Title/Abstract] OR Lithuania[Title/Abstract] OR Luxembourg[Title/Abstract] OR Moldova[Title/Abstract] OR Montenegro[Title/Abstract] OR Netherlands[Title/Abstract] OR Norway[Title/Abstract] OR Poland[Title/Abstract] OR Portugal[Title/Abstract] OR Romania[Title/Abstract] OR Serbia[Title/Abstract] OR Slovakia[Title/Abstract] OR Slovenia[Title/Abstract] OR Spain[Title/Abstract] OR Sweden[Title/Abstract] OR Switzerland[Title/Abstract] OR Ukraine[Title/Abstract] OR "United Kingdom"[Title/Abstract] OR England[Title/Abstract] OR Scotland[Title/Abstract] OR Wales[Title/Abstract]) |
| Europe PMC | (TITLE_ABS:Apodemus OR TITLE_ABS:"wood mouse" OR TITLE_ABS:"yellow-necked mouse" OR TITLE_ABS:"striped field mouse") AND (TITLE_ABS:"Escherichia coli" OR TITLE_ABS:"E. coli" OR TITLE_ABS:"antimicrobial resistance" OR TITLE_ABS:AMR OR TITLE_ABS:ESBL OR TITLE_ABS:"extended-spectrum beta-lactamase") AND (TITLE_ABS:Europe OR TITLE_ABS:European OR TITLE_ABS:Austria OR TITLE_ABS:Belarus OR TITLE_ABS:Belgium OR TITLE_ABS:Bosnia OR TITLE_ABS:Bulgaria OR TITLE_ABS:Croatia OR TITLE_ABS:Czechia OR TITLE_ABS:"Czech Republic" OR TITLE_ABS:Denmark OR TITLE_ABS:Estonia OR TITLE_ABS:Finland OR TITLE_ABS:France OR TITLE_ABS:Germany OR TITLE_ABS:Greece OR TITLE_ABS:Hungary OR TITLE_ABS:Ireland OR TITLE_ABS:Italy OR TITLE_ABS:Latvia OR TITLE_ABS:Lithuania OR TITLE_ABS:Luxembourg OR TITLE_ABS:Moldova OR TITLE_ABS:Montenegro OR TITLE_ABS:Netherlands OR TITLE_ABS:Norway OR TITLE_ABS:Poland OR TITLE_ABS:Portugal OR TITLE_ABS:Romania OR TITLE_ABS:Serbia OR TITLE_ABS:Slovakia OR TITLE_ABS:Slovenia OR TITLE_ABS:Spain OR TITLE_ABS:Sweden OR TITLE_ABS:Switzerland OR TITLE_ABS:Ukraine OR TITLE_ABS:"United Kingdom" OR TITLE_ABS:England OR TITLE_ABS:Scotland OR TITLE_ABS:Wales) |
| Web of Science | TS=((Apodemus OR "wood mouse" OR "yellow-necked mouse" OR "striped field mouse") AND ("Escherichia coli" OR "E. coli" OR "antimicrobial resistance" OR AMR OR ESBL OR "extended-spectrum beta-lactamase") AND (Europe OR European OR Austria OR Belarus OR Belgium OR Bosnia OR Bulgaria OR Croatia OR Czechia OR "Czech Republic" OR Denmark OR Estonia OR Finland OR France OR Germany OR Greece OR Hungary OR Ireland OR Italy OR Latvia OR Lithuania OR Luxembourg OR Moldova OR Montenegro OR Netherlands OR Norway OR Poland OR Portugal OR Romania OR Serbia OR Slovakia OR Slovenia OR Spain OR Sweden OR Switzerland OR Ukraine OR "United Kingdom" OR England OR Scotland OR Wales)) |
| CABI Digital Library | (Apodemus OR "wood mouse" OR "yellow-necked mouse" OR "striped field mouse") AND ("Escherichia coli" OR "E. coli" OR "antimicrobial resistance" OR AMR OR ESBL OR "extended-spectrum beta-lactamase") AND (Europe OR European OR Austria OR Belarus OR Belgium OR Bosnia OR Bulgaria OR Croatia OR Czechia OR "Czech Republic" OR Denmark OR Estonia OR Finland OR France OR Germany OR Greece OR Hungary OR Ireland OR Italy OR Latvia OR Lithuania OR Luxembourg OR Moldova OR Montenegro OR Netherlands OR Norway OR Poland OR Portugal OR Romania OR Serbia OR Slovakia OR Slovenia OR Spain OR Sweden OR Switzerland OR Ukraine OR "United Kingdom" OR England OR Scotland OR Wales) |

## Mycobacterium spp.

| **Database** | **Full search query** |
| --- | --- |
| PubMed | (Apodemus[Title/Abstract] OR "wood mouse"[Title/Abstract] OR "yellow-necked mouse"[Title/Abstract] OR "striped field mouse"[Title/Abstract]) AND (Mycobacterium[Title/Abstract] OR "Mycobacterium microti"[Title/Abstract] OR "Mycobacterium avium"[Title/Abstract] OR "non-tuberculous mycobacteria"[Title/Abstract] OR NTM[Title/Abstract] OR "M. microti"[Title/Abstract] OR "M. avium"[Title/Abstract]) AND (Europe[Title/Abstract] OR European[Title/Abstract] OR Austria[Title/Abstract] OR Belarus[Title/Abstract] OR Belgium[Title/Abstract] OR Bosnia[Title/Abstract] OR Bulgaria[Title/Abstract] OR Croatia[Title/Abstract] OR Czechia[Title/Abstract] OR "Czech Republic"[Title/Abstract] OR Denmark[Title/Abstract] OR Estonia[Title/Abstract] OR Finland[Title/Abstract] OR France[Title/Abstract] OR Germany[Title/Abstract] OR Greece[Title/Abstract] OR Hungary[Title/Abstract] OR Ireland[Title/Abstract] OR Italy[Title/Abstract] OR Latvia[Title/Abstract] OR Lithuania[Title/Abstract] OR Luxembourg[Title/Abstract] OR Moldova[Title/Abstract] OR Montenegro[Title/Abstract] OR Netherlands[Title/Abstract] OR Norway[Title/Abstract] OR Poland[Title/Abstract] OR Portugal[Title/Abstract] OR Romania[Title/Abstract] OR Serbia[Title/Abstract] OR Slovakia[Title/Abstract] OR Slovenia[Title/Abstract] OR Spain[Title/Abstract] OR Sweden[Title/Abstract] OR Switzerland[Title/Abstract] OR Ukraine[Title/Abstract] OR "United Kingdom"[Title/Abstract] OR England[Title/Abstract] OR Scotland[Title/Abstract] OR Wales[Title/Abstract]) |
| Europe PMC | (TITLE_ABS:Apodemus OR TITLE_ABS:"wood mouse" OR TITLE_ABS:"yellow-necked mouse" OR TITLE_ABS:"striped field mouse") AND (TITLE_ABS:Mycobacterium OR TITLE_ABS:"Mycobacterium microti" OR TITLE_ABS:"Mycobacterium avium" OR TITLE_ABS:"non-tuberculous mycobacteria" OR TITLE_ABS:NTM OR TITLE_ABS:"M. microti" OR TITLE_ABS:"M. avium") AND (TITLE_ABS:Europe OR TITLE_ABS:European OR TITLE_ABS:Austria OR TITLE_ABS:Belarus OR TITLE_ABS:Belgium OR TITLE_ABS:Bosnia OR TITLE_ABS:Bulgaria OR TITLE_ABS:Croatia OR TITLE_ABS:Czechia OR TITLE_ABS:"Czech Republic" OR TITLE_ABS:Denmark OR TITLE_ABS:Estonia OR TITLE_ABS:Finland OR TITLE_ABS:France OR TITLE_ABS:Germany OR TITLE_ABS:Greece OR TITLE_ABS:Hungary OR TITLE_ABS:Ireland OR TITLE_ABS:Italy OR TITLE_ABS:Latvia OR TITLE_ABS:Lithuania OR TITLE_ABS:Luxembourg OR TITLE_ABS:Moldova OR TITLE_ABS:Montenegro OR TITLE_ABS:Netherlands OR TITLE_ABS:Norway OR TITLE_ABS:Poland OR TITLE_ABS:Portugal OR TITLE_ABS:Romania OR TITLE_ABS:Serbia OR TITLE_ABS:Slovakia OR TITLE_ABS:Slovenia OR TITLE_ABS:Spain OR TITLE_ABS:Sweden OR TITLE_ABS:Switzerland OR TITLE_ABS:Ukraine OR TITLE_ABS:"United Kingdom" OR TITLE_ABS:England OR TITLE_ABS:Scotland OR TITLE_ABS:Wales) |
| Web of Science | TS=((Apodemus OR "wood mouse" OR "yellow-necked mouse" OR "striped field mouse") AND (Mycobacterium OR "Mycobacterium microti" OR "Mycobacterium avium" OR "non-tuberculous mycobacteria" OR NTM OR "M. microti" OR "M. avium") AND (Europe OR European OR Austria OR Belarus OR Belgium OR Bosnia OR Bulgaria OR Croatia OR Czechia OR "Czech Republic" OR Denmark OR Estonia OR Finland OR France OR Germany OR Greece OR Hungary OR Ireland OR Italy OR Latvia OR Lithuania OR Luxembourg OR Moldova OR Montenegro OR Netherlands OR Norway OR Poland OR Portugal OR Romania OR Serbia OR Slovakia OR Slovenia OR Spain OR Sweden OR Switzerland OR Ukraine OR "United Kingdom" OR England OR Scotland OR Wales)) |
| CABI Digital Library | (Apodemus OR "wood mouse" OR "yellow-necked mouse" OR "striped field mouse") AND (Mycobacterium OR "Mycobacterium microti" OR "Mycobacterium avium" OR "non-tuberculous mycobacteria" OR NTM OR "M. microti" OR "M. avium") AND (Europe OR European OR Austria OR Belarus OR Belgium OR Bosnia OR Bulgaria OR Croatia OR Czechia OR "Czech Republic" OR Denmark OR Estonia OR Finland OR France OR Germany OR Greece OR Hungary OR Ireland OR Italy OR Latvia OR Lithuania OR Luxembourg OR Moldova OR Montenegro OR Netherlands OR Norway OR Poland OR Portugal OR Romania OR Serbia OR Slovakia OR Slovenia OR Spain OR Sweden OR Switzerland OR Ukraine OR "United Kingdom" OR England OR Scotland OR Wales) |

## Staphylococcus aureus

| **Database** | **Full search query** |
| --- | --- |
| PubMed | (Apodemus[Title/Abstract] OR "wood mouse"[Title/Abstract] OR "yellow-necked mouse"[Title/Abstract] OR "striped field mouse"[Title/Abstract]) AND ("Staphylococcus aureus"[Title/Abstract] OR "S. aureus"[Title/Abstract] OR MRSA[Title/Abstract] OR "methicillin-resistant Staphylococcus aureus"[Title/Abstract] OR mecC[Title/Abstract]) AND (Europe[Title/Abstract] OR European[Title/Abstract] OR Austria[Title/Abstract] OR Belarus[Title/Abstract] OR Belgium[Title/Abstract] OR Bosnia[Title/Abstract] OR Bulgaria[Title/Abstract] OR Croatia[Title/Abstract] OR Czechia[Title/Abstract] OR "Czech Republic"[Title/Abstract] OR Denmark[Title/Abstract] OR Estonia[Title/Abstract] OR Finland[Title/Abstract] OR France[Title/Abstract] OR Germany[Title/Abstract] OR Greece[Title/Abstract] OR Hungary[Title/Abstract] OR Ireland[Title/Abstract] OR Italy[Title/Abstract] OR Latvia[Title/Abstract] OR Lithuania[Title/Abstract] OR Luxembourg[Title/Abstract] OR Moldova[Title/Abstract] OR Montenegro[Title/Abstract] OR Netherlands[Title/Abstract] OR Norway[Title/Abstract] OR Poland[Title/Abstract] OR Portugal[Title/Abstract] OR Romania[Title/Abstract] OR Serbia[Title/Abstract] OR Slovakia[Title/Abstract] OR Slovenia[Title/Abstract] OR Spain[Title/Abstract] OR Sweden[Title/Abstract] OR Switzerland[Title/Abstract] OR Ukraine[Title/Abstract] OR "United Kingdom"[Title/Abstract] OR England[Title/Abstract] OR Scotland[Title/Abstract] OR Wales[Title/Abstract]) |
| Europe PMC | (TITLE_ABS:Apodemus OR TITLE_ABS:"wood mouse" OR TITLE_ABS:"yellow-necked mouse" OR TITLE_ABS:"striped field mouse") AND (TITLE_ABS:"Staphylococcus aureus" OR TITLE_ABS:"S. aureus" OR TITLE_ABS:MRSA OR TITLE_ABS:"methicillin-resistant Staphylococcus aureus" OR TITLE_ABS:mecC) AND (TITLE_ABS:Europe OR TITLE_ABS:European OR TITLE_ABS:Austria OR TITLE_ABS:Belarus OR TITLE_ABS:Belgium OR TITLE_ABS:Bosnia OR TITLE_ABS:Bulgaria OR TITLE_ABS:Croatia OR TITLE_ABS:Czechia OR TITLE_ABS:"Czech Republic" OR TITLE_ABS:Denmark OR TITLE_ABS:Estonia OR TITLE_ABS:Finland OR TITLE_ABS:France OR TITLE_ABS:Germany OR TITLE_ABS:Greece OR TITLE_ABS:Hungary OR TITLE_ABS:Ireland OR TITLE_ABS:Italy OR TITLE_ABS:Latvia OR TITLE_ABS:Lithuania OR TITLE_ABS:Luxembourg OR TITLE_ABS:Moldova OR TITLE_ABS:Montenegro OR TITLE_ABS:Netherlands OR TITLE_ABS:Norway OR TITLE_ABS:Poland OR TITLE_ABS:Portugal OR TITLE_ABS:Romania OR TITLE_ABS:Serbia OR TITLE_ABS:Slovakia OR TITLE_ABS:Slovenia OR TITLE_ABS:Spain OR TITLE_ABS:Sweden OR TITLE_ABS:Switzerland OR TITLE_ABS:Ukraine OR TITLE_ABS:"United Kingdom" OR TITLE_ABS:England OR TITLE_ABS:Scotland OR TITLE_ABS:Wales) |
| Web of Science | TS=((Apodemus OR "wood mouse" OR "yellow-necked mouse" OR "striped field mouse") AND ("Staphylococcus aureus" OR "S. aureus" OR MRSA OR "methicillin-resistant Staphylococcus aureus" OR mecC) AND (Europe OR European OR Austria OR Belarus OR Belgium OR Bosnia OR Bulgaria OR Croatia OR Czechia OR "Czech Republic" OR Denmark OR Estonia OR Finland OR France OR Germany OR Greece OR Hungary OR Ireland OR Italy OR Latvia OR Lithuania OR Luxembourg OR Moldova OR Montenegro OR Netherlands OR Norway OR Poland OR Portugal OR Romania OR Serbia OR Slovakia OR Slovenia OR Spain OR Sweden OR Switzerland OR Ukraine OR "United Kingdom" OR England OR Scotland OR Wales)) |
| CABI Digital Library | (Apodemus OR "wood mouse" OR "yellow-necked mouse" OR "striped field mouse") AND ("Staphylococcus aureus" OR "S. aureus" OR MRSA OR "methicillin-resistant Staphylococcus aureus" OR mecC) AND (Europe OR European OR Austria OR Belarus OR Belgium OR Bosnia OR Bulgaria OR Croatia OR Czechia OR "Czech Republic" OR Denmark OR Estonia OR Finland OR France OR Germany OR Greece OR Hungary OR Ireland OR Italy OR Latvia OR Lithuania OR Luxembourg OR Moldova OR Montenegro OR Netherlands OR Norway OR Poland OR Portugal OR Romania OR Serbia OR Slovakia OR Slovenia OR Spain OR Sweden OR Switzerland OR Ukraine OR "United Kingdom" OR England OR Scotland OR Wales) |

## Search results

| **Pathogen block** | **Total** |
| --- | --- |
| Arenaviridae/LCMV | 152 |
| Hantaviridae/orthohantaviruses | 753 |
| Picornaviridae | 141 |
| Poxviridae/cowpox virus | 191 |
| Francisella tularensis | 228 |
| Leptospira spp. | 325 |
| Yersinia spp. | 249 |
| Astroviridae | 145 |
| Coronaviridae | 494 |
| Paramyxoviridae | 247 |
| Campylobacter spp. | 149 |
| Clostridioides difficile | 122 |
| Escherichia coli | 303 |
| Mycobacterium spp. | 310 |
| Staphylococcus aureus | 242 |
| Total | 4,051 |

**Note.** Values are pooled across the four databases for each pathogen-specific search block after duplicate removal within that block. They are not database-specific retrieval counts or counts of unique publications across the review. The 15 pathogen-block totals sum to the 4,051 records screened in the PRISMA flow diagram. Broad host–pathogen sensitivity searches and selected country–pathogen searches yielded no additional eligible records and were not included as a separate PRISMA retrieval stream.
