# Supplementary material for: Small rodents, huge impact: A scoping review of European Apodemus species in the ecology of directly transmitted, environmentally mediated, and interface-associated zoonotic pathogens
Source: One Health. 2026 Jul 28;23:101532. doi: 10.1016/j.onehlt.2026.101532 (PMC13452164; doi:10.1016/j.onehlt.2026.101532)
Supplement: Supplementary file 2 [file mmc2.docx]

| Criterion domain | Inclusion criteria | Exclusion criteria |
| --- | --- | --- |
| Publication type | Peer-reviewed primary research articles | Reviews, editorials, opinion papers, book chapters, theses, conference abstracts, and grey literature |
| Publication period | Published between 2000 and 2025 | Published before 2000 or outside the search period |
| Language | English-language full text | Non-English full text |
| Geographic scope | Sampling conducted in Europe, defined according to the UNSD M49 classification, excluding the Asian portion of the Russian Federation | Non-European sampling or records from the Asian portion of the Russian Federation |
| Host scope | Wild, free-ranging, synanthropic, or peri-domestic *Apodemus* spp.; host identified at least at genus level | No *Apodemus* data, or pathogen results not attributable to *Apodemus* at genus or species level |
| Study setting | Natural infection, exposure, carriage, or detection in field-derived *Apodemus* populations | Captive or laboratory-only studies unless they included original field-derived European *Apodemus* data |
| Pathogen scope | Zoonotic viral or bacterial pathogens transmitted directly, environmentally, foodborne, or through non-vector-borne routes within the scope of the review | Vector-borne pathogens outside the review scope; human-only, livestock-only, or non-*Apodemus* wildlife-only studies |
| Diagnostic evidence | Positive PCR, RT-PCR, culture, isolation, sequencing, metagenomics, serology, antigen detection, microscopy, or equivalent pathogen evidence attributable to *Apodemus* spp. | No positive *Apodemus*-specific pathogen evidence |
| Full-text availability | Full text obtainable for eligibility assessment | Full text unobtainable after reasonable retrieval attempts |
| Data extractability | Number tested and number positive, prevalence/seroprevalence, or otherwise interpretable positive detection/exposure data | Neither a denominator nor an otherwise interpretable Apodemus-specific detection or exposure result; or results pooled across host taxa without Apodemus-specific attribution |

**Table S2. Inclusion and exclusion criteria applied during study selection.**

Eligibility criteria used to identify peer-reviewed European studies reporting documented positive diagnostic, serological, molecular, culture-based, metagenomic, or otherwise interpretable evidence of zoonotic viral or bacterial pathogens in *Apodemus* spp. The review was designed to map documented positive *Apodemus*-associated pathogen evidence rather than estimate pathogen absence or zero prevalence. Studies in which *Apodemus* spp. were tested but reported exclusively negative results were therefore outside the scope of the main evidence map.
