# Supplementary material for: Small rodents, huge impact: A scoping review of European Apodemus species in the ecology of directly transmitted, environmentally mediated, and interface-associated zoonotic pathogens
Source: One Health. 2026 Jul 28;23:101532. doi: 10.1016/j.onehlt.2026.101532 (PMC13452164; doi:10.1016/j.onehlt.2026.101532)
Supplement: Supplementary file 3 [file mmc3.docx]

Supplementary Table S3: Viral pathogens detected in European Apodemus populations with extractable denominators. The table reports denominator-based positive records only. Percentages were calculated as n/N × 100 and rounded to one decimal place. Broad pathogen labels, such as “hantavirus” or “LCMV / arenavirus”, are used where the original extraction did not provide finer species- or genotype-level resolution. Rows were sorted alphabetically by pathogen group, then country, host species, and reference.

† Sample-level result; n/N denotes positive samples over total samples rather than positive animals over animals tested; ns, not specified. Records with positive Apodemus-specific evidence but no extractable denominator were moved to detection-only supplementary tables and were not used for percentage calculations. Rows marked as samples report sample-level positivity rather than individual-level prevalence.

| Arenaviridae | | | | | | | | | | |
| --- | --- | --- | --- | --- | --- | --- | --- | --- | --- | --- |
| Host species | Pathogen(s) | | n/N | (%) | | Method | | Country | | References |
| *A. agrarius* | LCMV / arenavirus | | 3/26 | 11.5 | | IFA | | Croatia + Slovenia | | [1] |
| *A. flavicollis* | LCMV / arenavirus | | 4/21 | 19.0 | | IFA | | Croatia + Slovenia | | [1] |
| *A. sylvaticus* | LCMV / arenavirus | | 4/16 | 25.0 | | IFA | | Croatia + Slovenia | | [1] |
| *A. agrarius* | LCMV / arenavirus | | 10/63 | 15.9 | | IFA | | Finland | | [2] |
| *A. flavicollis* | LCMV / arenavirus | | 6/105 | 5.7 | | IFA | | Finland | | [2] |
| *A. flavicollis* | LCMV / arenavirus | | 2/26 | 7.7 | | Antibody assays | | Finland | | [3] |
| *A. sylvaticus* | LCMV | | 2/13 | 15.4 | | RT-PCR | | Germany | | [4] |
| *A. flavicollis* | LCMV / arenavirus | | 205/2732† | 7.5 | | Antibody assays | | Italy | | [5] |
| *A. flavicollis* | LCMV / arenavirus | | 72/1181 | 6.1 | | IFA | | Italy | | [6] |
| *A. sylvaticus* | LCMV | | 21/536 | 3.9 | | Antibody assays | | Spain | | [7] |
| *A. sylvaticus* | LCMV / arenavirus | | 2/34 | 5.9 | | IFA | | Spain | | [8] |
| *A. sylvaticus* | LCMV / arenavirus | | 6/217 | 2.8 | | Antibody assays | | United Kingdom | | [9] |
| Hantaviridae | | | | | | | | | | |
| Host species | Pathogen(s) | | n/N | | (%) | | Method | | Country | References |
| *A. flavicollis* | hantavirus | | 10/148 | | 6.8 | | Genetic sequencing | | Albania | [10] |
| *A. agrarius* | hantavirus | | 2/35 | | 5.7 | | RT-PCR | | Austria | [11] |
| *A. sylvaticus* | hantavirus | | 14/35 | | 40.0 | | ELISA | | Belgium | [12] |
| *A. agrarius* | hantavirus | | 2/102 | | 2.0 | | RT-PCR | | Bulgaria | [13] |
| *A. flavicollis* | hantavirus | | 7/92 | | 7.6 | | RT-PCR | | Bulgaria | [13] |
| *A. agrarius* | hantavirus | | 1/234 | | 0.4 | | RT-PCR | | Bulgaria | [14] |
| *A. flavicollis* | hantavirus | | 7/333 | | 2.1 | | RT-PCR | | Bulgaria | [14] |
| *A. agrarius* | hantavirus | | 1/15 | | 6.7 | | ELISA | | Croatia | [15] |
| *A. flavicollis* | hantavirus | | 3/27 | | 11.1 | | RT-PCR | | Croatia | [15] |
| *A. agrarius* | hantavirus | | 2/18 | | 11.1 | | WB | | Croatia | [16] |
| *A. flavicollis* | hantavirus | | 6/66 | | 9.1 | | WB | | Croatia | [16] |
| *A. sylvaticus* | hantavirus | | 12/151 | | 7.9 | | WB | | Croatia | [16] |
| *A. flavicollis* | hantavirus | | 21/86 | | 24.4 | | RT-PCR | | Croatia | [17] |
| *A. flavicollis* | hantavirus | | 16/28 | | 57.1 | | IFA | | Croatia | [18] |
| *A. flavicollis* | hantavirus | | 6/61 | | 9.8 | | RT-PCR | | Croatia | [19] |
| *A. agrarius* | hantavirus | | 36/131 | | 27.5 | | PCR | | Croatia | [20] |
| *A. flavicollis* | hantavirus | | 2/53 | | 3.8 | | PCR | | Croatia | [20] |
| *A. agrarius* | hantavirus | | 12/46 | | 26.1 | | RT-PCR | | Czechia | [21] |
| *A. flavicollis* | hantavirus | | 2/38 | | 5.3 | | RT-PCR | | Czechia | [21] |
| *A. sylvaticus* | hantavirus | | 4/25 | | 16.0 | | RT-PCR | | Czechia | [21] |
| *A. flavicollis* | hantavirus | | 1/77 | | 1.3 | | RT-PCR | | Czechia | [22] |
| *A. sylvaticus* | hantavirus | | 1/34 | | 2.9 | | RT-PCR | | Czechia | [22] |
| *A. agrarius* | hantavirus | | 1/127 | | 0.8 | | RT-PCR | | Denmark | [23] |
| *A. agrarius* | hantavirus | | 4/90 | | 4.4 | | ELISA | | Estonia | [24] |
| *A. agrarius* | hantavirus | | 1/63 | | 1.6 | | IFA | | Finland | [2] |
| *A. sylvaticus* | hantavirus | | 1/151 | | 0.7 | | IFA | | France | [25] |
| *A. flavicollis* | hantavirus | | 26/366 | | 7.1 | | ELISA | | Germany | [26] |
| *A. agrarius* | hantavirus | | 3/11 | | 27.3 | | PCR | | Hungary | [27] |
| *A. agrarius* | hantavirus | | 10/148 | | 6.8 | | RT-PCR | | Hungary | [28] |
| *Apodemus* spp. | hantavirus | | 114/661 | | 17.2 | | ELISA | | Hungary | [29] |
| *A. agrarius* | hantavirus | | 6/52 | | 11.5 | | ELISA | | Hungary | [15] |
| *A. flavicollis* | hantavirus | | 2/31 | | 6.5 | | RT-PCR | | Hungary | [15] |
| *Apodemus* spp. | hantavirus | | 38/362 | | 10.5 | | RT-PCR | | Hungary | [30] |
| *A. flavicollis* | hantavirus | | 3/1416 | | 0.2 | | Antibody assay | | Italy | [6] |
| *A. flavicollis* | hantavirus | | 4/22 | | 18.2 | | Genetic sequencing | | Italy | [31] |
| *Apodemus* spp. | hantavirus | | 38/1887 | | 2.0 | | IFA | | Italy | [32] |
| *A. agrarius* | hantavirus | | 24/442 | | 5.4 | | RT-PCR | | Lithuania | [33] |
| *A. agrarius* | hantavirus | | 1/47 | | 2.1 | | RT-PCR | | Poland | [34] |
| *A. flavicollis* | hantavirus | | 9/57 | | 15.8 | | RT-PCR | | Poland | [34] |
| *A. agrarius* | hantavirus | | 2/42 | | 4.8 | | RT-PCR | | Poland | [35] |
| *A. agrarius* | hantavirus | | 1/4 | | 25.0 | | ELISA | | Poland | [36] |
| *A. flavicollis* | hantavirus | | 35/401 | | 8.7 | | ELISA | | Poland | [36] |
| *A. flavicollis* | hantavirus | | 3/31 | | 9.7 | | PCR | | Romania | [37] |
| *A. agrarius* | hantavirus | | 1/60 | | 1.7 | | IFA | | Russia | [38] |
| *A. flavicollis* | hantavirus | | 20/39 | | 51.3 | | IFA | | Russia | [38] |
| *A. flavicollis* | hantavirus | | 16/30 | | 53.3 | | ELISA | | Russia | [39] |
| *A. flavicollis* | hantavirus | | 1/19 | | 5.3 | | IFA | | Russia | [40] |
| *A. flavicollis* | hantavirus | | 22/185 | | 11.9 | | IFA | | Serbia | [41] |
| *A. agrarius* | hantavirus | | 14/82 | | 17.1 | | RT-PCR | | Slovakia | [42] |
| *A. agrarius* | hantavirus | | 42/306 | | 13.7 | | RT-PCR | | Slovakia | [43] |
| *A. flavicollis* | hantavirus | | 2/55 | | 3.6 | | RT-PCR | | Slovakia | [43] |
| *Apodemus* spp. | hantavirus | | 53/260 | | 20.4 | | ns | | Slovenia | [44] |
| *A. flavicollis* | hantavirus | | 10/129 | | 7.8 | | ELISA | | Sweden | [45] |
| *A. agrarius* | hantavirus | | 9/404 | | 2.2 | | IFA | | Ukraine | [46] |
| *A. agrarius* | hantavirus | | 21/62 | | 33.9 | | IFA | | Ukraine | [47] |
| *A. flavicollis* | hantavirus | | 25/122 | | 20.5 | | IFA | | Ukraine | [47] |
| Paramyxoviridae | | | | | | | | | | |
| Host species | | Pathogen(s) | n/N | | (%) | | Method | | Country | References |
| A. flavicollis | | ns | 8/18 | | 44.4 | | RT-PCR | | Belgium | [48] |
| A. sylvaticus | | ns | 1/3 | | 33.3 | | RT-PCR | | Belgium | [49] |
| A. flavicollis | | ns | 1/1 | | 100.0 | | RT-PCR | | Germany | [50] |
| Picornaviridae | | | | | | | | | | |
| Host species | | Pathogen(s) | n/N | | (%) | | Method | | Country | References |
| A. agrarius | | Ljungan virus | 4/48 | | 8.3 | | RT-PCR | | Germany | [51] |
| A. flavicollis | | Ljungan virus | 12/93 | | 12.9 | | RT-PCR | | Germany | [51] |
| A. sylvaticus | | Ljungan virus | 1/18 | | 5.6 | | RT-PCR | | Germany | [51] |
| A. sylvaticus | | Encephalomyocarditis virus | 1/15 | | 6.7 | | VNT | | Greece | [52] |
| A. agrarius | | Ljungan virus | 5/135 | | 3.7 | | RT-PCR | | Italy | [53] |
| A. flavicollis | | Ljungan virus | 2/20 | | 10.0 | | RT-PCR | | Italy | [54] |
| A. sylvaticus | | Ljungan virus | 13/66 | | 19.7 | | PCR | | United Kingdom | [55] |
| Poxviridae | | | | | | | | | | |
| Host species | | Pathogen(s) | n/N | | (%) | | Method | | Country | References |
| *A. flavicollis* | | Orthopoxvirus / cowpox virus | 7/43 | | 16.3 | | IFA | | France | [25] |
| A. sylvaticus | | Orthopoxvirus / cowpox virus | 10/151 | | 6.6 | | IFA | | France | [25] |
| *A. flavicollis* | | Orthopoxvirus / cowpox virus | 27/326 | | 8.3 | | SNT | | Germany | [56] |
| A. sylvaticus | | Orthopoxvirus / cowpox virus | 2/47 | | 4.3 | | SNT | | Germany | [56] |
| A. agrarius | | Orthopoxvirus / cowpox virus | 14/22 | | 63.6 | | IFA | | Germany | [57] |
| A. agrarius | | Orthopoxvirus / cowpox virus | 15/36 | | 41.7 | | IFA | | Hungary | [58] |
| *A. flavicollis* | | Orthopoxvirus / cowpox virus | 50/1114 | | 4.5 | | IFA | | Hungary | [58] |
| A. sylvaticus | | Orthopoxvirus / cowpox virus | 6/122 | | 4.9 | | IFA | | Hungary | [58] |

| Supplementary Table S4. Bacterial pathogens detected in European Apodemus populations with extractable denominators. The table reports denominator-based positive records only. Percentages were calculated as n/N × 100 and rounded to one decimal place. Pathogen species, serovars, strains, or genotypes are reported where available; “ns” indicates that the pathogen was not specified beyond the reported taxonomic level. Rows were sorted alphabetically by pathogen group, then country, host species, and reference. For *Yersinia* spp., records including *Y. enterocolitica* and/or *Y. pseudotuberculosis* were interpreted as potentially zoonotic evidence. Records in which these species were pooled with other *Yersinia* congeners, or where *Yersinia* was not resolved to species level, were retained as detection context but were not interpreted as species-specific prevalence of pathogenic *Yersinia*.  † Sample-level result; n/N denotes positive samples over total samples rather than positive animals over animals tested; ns, not specified. Records with positive Apodemus-specific evidence but no extractable denominator were moved to detection-only supplementary tables and were not used for percentage calculations. Rows marked as samples report sample-level positivity rather than individual-level prevalence. | | | | | | |
| --- | --- | --- | --- | --- | --- | --- |
| Campylobacter spp. | | | | | | |
| Host species | Pathogen(s) | n/N | (%) | Method | Country | References |
| A. flavicollis | C. jejuni | 59/89 | 66.3 | WGS | Finland | [59] |
| A. flavicollis | C. jejuni | 217/482 | 45.0 | PCR | Finland | [60] |
| A. flavicollis | C. jejuni | 27/65 | 41.5 | PCR | Finland | [61] |
| A. flavicollis | C. jejuni | 7/18 | 38.9 | PCR | Sweden | [62] |
| A. flavicollis | C. upsaliensis | 2/18 | 11.1 | PCR | Sweden | [62] |
| A. flavicollis | C. jejuni | 1/45 | 2.2 | Culture | Sweden | [63] |
| Clostridioides difficile | | | | | | |
| Host species | Pathogen(s) | n/N | (%) | Method | Country | References |
| A. sylvaticus | C. difficile | 1/19 | 5.3 | PCR | Netherlands | [64] |
| A. sylvaticus | C. difficile | 2/13 | 15.4 | PCR | Netherlands | [65] |
| Apodemus spp. | C. difficile | 7/53 | 13.2 | PCR | Spain | [66] |
| Escherichia coli | | | | | | |
| Host species | Pathogen(s) | n/N | (%) | Method | Country | References |
| A. flavicollis | E. coli | 3/7 | 42.9 | PCR | Bulgaria | [67] |
| A. flavicollis | E. coli | 5/482 | 1.0 | PCR | Finland | [60] |
| A. sylvaticus | E. coli | 3/25 | 12.0 | PCR | France | [68] |
| A. agrarius | E. coli | 32/124 | 25.8 | PCR | Germany | [69] |
| A. flavicollis | E. coli | 65/408 | 15.9 | PCR | Germany | [69] |
| A. sylvaticus | E. coli | 13/95 | 13.7 | PCR | Germany | [69] |
| A. sylvaticus | E. coli | 2/176 | 1.1 | PCR | Netherlands | [70] |
| A. sylvaticus | E. coli | 12/95 | 12.6 | PCR | Spain | [71] |
| A. sylvaticus | E. coli | 1/59† | 1.7 | PCR | United Kingdom | [72] |
| A. sylvaticus | E. coli | 241/1012 † | 23.8 | PCR | United Kingdom | [73] |
| *Francisella tularensis* | | | | | | |
| Host species | Pathogen(s) | n/N | (%) | Method | Country | References |
| *A. agrarius* | *F. tularensis* | 2/197 | 1.0 | PCR | Croatia | [74] |
| *A. agrarius* | *F. tularensis* | 2/53 | 3.8 | PCR | Croatia | [20] |
| *A. sylvaticus* | *F. tularensis* | 1/78 | 1.3 | PCR | Croatia | [74] |
| *A. agrarius* | *F. tularensis* | 2/40 | 5.0 | PCR | Czechia | [75] |
| *A. flavicollis* | *F. tularensis* | 18/324 | 5.6 | PCR | Czechia | [75] |
| *A. flavicollis* | *F. tularensis* | 2/37 | 5.4 | ELISA | Czechia | [76] |
| *A. flavicollis* | *F. tularensis* | 34/168 | 20.2 | ELISA | Czechia | [77] |
| *A. sylvaticus* | *F. tularensis* | 1/48 | 2.1 | PCR | Czechia | [75] |
| *A. sylvaticus* | *F. tularensis* | 1/10 | 10.0 | ELISA | Czechia | [76] |
| *A. flavicollis* | *F. tularensis* | 2/69 | 2.9 | PCR | Germany | [78] |
| *A. agrarius* | *F. tularensis* | 1/75 | 1.3 | PCR | Russia | [79] |
| *A. flavicollis* | *F. tularensis* | 15/39 | 38.5 | RT-PCR | Russia | [38] |
| *A. flavicollis* | *F. tularensis* | 4/30 | 13.3 | ELISA | Russia | [39] |
| *A. sylvaticus* | *F. tularensis* | 1/336 | 0.3 | PCR | Russia | [79] |
| *A. agrarius* | *F. tularensis* | 13/72 | 18.1 | PCR | Serbia | [80] |
| *A. sylvaticus* | *F. tularensis* | 3/22 | 13.6 | PCR | Spain | [81] |
| *Leptospira* spp. | | | | | | |
| Host species | Pathogen(s) | n/N | (%) | Method | Country | References |
| *A. agrarius* | *L. kirschneri; L. borgpetersenii* | 1/35 | 2.9 | RT-PCR | Austria | [11] |
| *A. sylvaticus* | *L. kirschneri* | 2/26 | 7.7 | RT-PCR | Austria | [82] |
| *Apodemus* spp. | *ns* | 15/53 | 28.3 | PCR | Bulgaria | [83] |
| *A. agrarius* | serovars: Grippotyphosa; Icterohaemorrhagiae; Pomona; Serjoe; Tarassovi; Hardjo | 16/108 | 14.8 | Antibody titre | Croatia | [84] |
| *A. agrarius* | *L. interrogans* | 15/108 | 13.9 | MAT | Croatia | [85] |
| *A. agrarius* | *L. kirschneri; L. interrogans; L. borgpetersenii* | 29/96 | 30.2 | PCR | Croatia | [86] |
| *A. agrarius* | *L. kirschneri* | 10/29 | 34.5 | PCR | Croatia | [87] |
| *A. agrarius* | *L. interrogans; L. kirschneri; L. borgpetersenii* | 12/53 | 22.6 | PCR | Croatia | [20] |
| *A. flavicollis* | serovars: Australis; Pomona; Saxkoebing; Batavie | 12/111 | 10.8 | Antibody titre | Croatia | [84] |
| *A. flavicollis* | *L. interrogans* | 12/111 | 10.8 | MAT | Croatia | [85] |
| *A. flavicollis* | *L. kirschneri; L. interrogans; L. borgpetersenii* | 32/262 | 12.2 | PCR | Croatia | [86] |
| *A. flavicollis* | *L. interrogans; L. borgpetersenii* | 5/20 | 25.0 | PCR | Croatia | [87] |
| *A. flavicollis* | serovars: Australis; Grippotyphosa | 13/44 | 29.5 | MAT | Croatia | [18] |
| *A. flavicollis* | *L. interrogans; L. kirschneri; L. borgpetersenii* | 37/131 | 28.2 | PCR | Croatia | [20] |
| *A. sylvaticus* | serovars: Serjoe; Pomona; Tarassovi | 4/62 | 6.5 | Antibody titre | Croatia | [84] |
| *A. sylvaticus* | *L. interrogans* | 4/62 | 6.5 | MAT | Croatia | [85] |
| *A. sylvaticus* | *L. kirschneri; L. interrogans* | 22/122 | 18.0 | PCR | Croatia | [86] |
| *A. sylvaticus* | *L. interrogans* | 3/9 | 33.3 | PCR | Croatia | [87] |
| *A. agrarius* | *L. interrogans* | 1/32 | 3.1 | PCR | Czechia | [88] |
| *A. agrarius* | *L. interrogans* | 7/50 | 14.0 | MAT | Czechia | [89] |
| *A. flavicollis* | serovar: Grippotyphosa | 1/17 | 5.9 | MAT | Czechia | [76] |
| *A. flavicollis* | *L. interrogans* | 21/497 | 4.2 | MAT | Czechia | [89] |
| *A. sylvaticus* | *L. interrogans* | 6/57 | 10.5 | MAT | Czechia | [89] |
| *Apodemus* spp. | *L. interrogans* | 17/297 | 5.7 | PCR | Czechia | [88] |
| *Apodemus* spp. | serovar: Grippotyphosa | 17/165 | 10.3 | MAT | Czechia | [90] |
| *Apodemus* spp. | serovars: Grippotyphosa; Bulgarica | 74/981 | 7.5 | MAT | Czechia | [91] |
| *A. flavicollis* | *L. interrogans* | 7/38 | 18.4 | PCR | France | [92] |
| *A. sylvaticus* | *L. borgpetersenii* | 1/13 | 7.7 | PCR | France | [93] |
| *A. sylvaticus* | *L. interrogans* | 28/255 | 11.0 | PCR | France | [92] |
| *A. agrarius* | *L. kirschneri* | 6/44 | 13.6 | PCR | Germany | [94] |
| *A. agrarius* | *L. kirschneri* | 19/86 | 22.1 | PCR | Germany | [95] |
| *A. agrarius* | *L. kirschneri* | 23/190 | 12.1 | PCR | Germany | [96] |
| *A. agrarius* | *L. kirschneri* | 3/7 | 42.9 | PCR | Germany | [97] |
| *A. flavicollis* | *ns* | 2/4 | 50.0 | PCR | Germany | [98] |
| *A. flavicollis* | *L. kirschneri; L. interrogans; L. borgpetersenii* | 122/925 | 13.2 | PCR | Germany | [94] |
| *A. flavicollis* | *L. kirschneri; L. borgpetersenii* | 55/345 | 15.9 | PCR | Germany | [95] |
| *A. flavicollis* | *L. kirschneri; L. interrogans; L. borgpetersenii* | 71/792 | 9.0 | PCR | Germany | [96] |
| *A. flavicollis* | *L. kirschneri; L. borgpetersenii* | 12/247 | 4.9 | PCR | Germany | [97] |
| *A. sylvaticus* | *L. interrogans* | 9/105 | 8.6 | PCR | Germany | [94] |
| *A. sylvaticus* | *L. kirschneri* | 8/60 | 13.3 | PCR | Germany | [95] |
| *A. sylvaticus* | *L. kirschneri; L. interrogans* | 27/154 | 17.5 | PCR | Germany | [96] |
| *Apodemus* spp. | *L. kirschneri; L. interrogans; L. borgpetersenii* | 12/184 | 6.5 | PCR | Germany | [97] |
| *A. agrarius* | *ns* | 29/148 | 19.6 | PCR | Hungary | [28] |
| *A. flavicollis* | *ns* | 4/35 | 11.4 | PCR | Hungary | [28] |
| *A. sylvaticus* | *ns* | 1/29 | 3.4 | PCR | Hungary | [28] |
| *A. sylvaticus* | *L. interrogans* | 2/13 | 15.4 | PCR | Italy | [99] |
| *A. sylvaticus* | *L. interrogans; L. borgpetersenii* | 19/60 | 31.7 | PCR | Italy | [100] |
| *A. agrarius* | *L. kirschneri* | 16/432 | 3.7 | RT-PCR | Lithuania | [33] |
| *A. flavicollis* | *L. kirschneri* | 30/380 | 7.9 | RT-PCR | Lithuania | [33] |
| *A. agrarius* | serovars: Grippotyphosa; Icterohaemorrhagiae; Pomona | 35/315 approx. | ns | Serological assay | Moldova | [101] |
| *A. agrarius* | serovar: Pomona | 2/235 approx. | ns | Serological assay | Moldova | [101] |
| *A. agrarius* | serovars: Grippotyphosa; Icterohaemorrhagiae; Ballum; Pomona | 18/410 | 4.4 | MAT | Moldova | [102] |
| *A. flavicollis* | serovars: Grippotyphosa; Pomona; Autumnalis | 9/140 | 6.4 | MAT | Moldova | [102] |
| *A. sylvaticus* | serovar: Pomona | 2/235 approx. | ns | Serological assay | Moldova | [101] |
| *A. sylvaticus* | serovars: Grippotyphosa; Pomona | 17/158 | 10.8 | MAT | Moldova | [102] |
| *A. uralensis* | serovar: Grippotyphosa | 2/86 | 2.3 | MAT | Moldova | [102] |
| *A. flavicollis* | *ns* | 8/29 | 27.6 | PCR | Netherlands | [70] |
| *A. sylvaticus* | *L. interrogans* | 2/19 | 10.5 | RT-PCR | Netherlands | [103] |
| *A. sylvaticus* | *ns* | 19/374 | 5.1 | PCR | Netherlands | [70] |
| *A. agrarius* | *L. kirschneri* | 14/39 | 35.9 | PCR | Poland | [104] |
| *A. agrarius* | *ns* | 7/7 | 100.0 | PCR | Serbia | [105] |
| *A. flavicollis* | *ns* | 12/54 | 22.2 | PCR | Serbia | [105] |
| *A. sylvaticus* | *L. kirschneri; L. interrogans; L. borgpetersenii* | 30/266 | 11.3 | PCR | Spain | [106] |
| *A. flavicollis* | *ns* | 1/5 | 20.0 | PCR | Sweden | [62] |
| *A. sylvaticus* | *ns* | 7/60 | 11.7 | PCR | Switzerland | [107] |
| *A. agrarius* | *ns* | 104/724 | 14.4 | MAT | Ukraine | [108] |
| *A. agrarius* | *ns* | 140/1953 | 7.2 | MAT | Ukraine | [109] |
| *A. flavicollis* | *ns* | 11/117 | 9.4 | MAT | Ukraine | [108] |
| *A. sylvaticus* | *ns* | 20/249 | 8.0 | MAT | Ukraine | [108] |
| Mycobacterium spp. | | | | | | |
| Host species | Pathogen(s) | n/N | (%) | Method | Country | References |
| A. flavicollis | M. avium; ns | 3/15 | 20.0 | Culture | Czechia & Slovakia | [110] |
| A. flavicollis | M. microti | 6/75 | 8.0 | RT-PCR | Italy | [111] |
| A. sylvaticus | ns | 1/30 | 3.3 | RT-PCR | Italy | [111] |
| A. sylvaticus | M. fortuitum; M. intracellulare; M. gordonae; M. celatum; M. avium | 6/94 | 6.4 | PCR & culture | Spain | [112] |
| Staphylococcus aureus | | | | | | |
| Host species | Pathogen(s) | n/N | (%) | Method | Country | References |
| A. flavicollis | S. aureus | 11/61 | 18.0 | PCR | France | [68] |
| A. sylvaticus | S. aureus | 1/25 | 4.0 | PCR | Germany | [113] |
| A. sylvaticus | S. aureus | 2/13 | 15.4 | PCR | Netherlands | [65] |
| A. sylvaticus | S. aureus | 12/174 | 6.9 | PCR | Netherlands | [70] |
| A. sylvaticus | S. aureus | 4/29 | 13.8 | PCR | Spain | [114] |
| Yersinia spp  ‡ At least one potentially zoonotic Yersinia species—Y. enterocolitica and/or Y. pseudotuberculosis—was reported. § Potentially zoonotic and other Yersinia congeners were pooled; n/N and positivity cannot be attributed to an individual pathogenic species. ¶ Yersinia was not resolved to species level; zoonotic relevance is uncertain. | | | | | | |
| Host species | Pathogen(s) | n/N | (%) | Method | Country | References |
| *A. flavicollis* | *Y. enterocolitica; Y. kristensenii‡§* | 2/7 | 28.6 | PCR | Bulgaria | [67] |
| *A. agrarius* | *Y. enterocolitica; Y. kristensenii; Y. mollaretii/Y. bercovieri; Y. frederiksenii; Y. intermedia‡§* | 13/52 | 25.0 | PCR | Finland | [115] |
| *A. flavicollis* | *Y. enterocolitica; Y. kristensenii; Y. mollaretii/Y. bercovieri; Y. frederiksenii; Y. intermedia‡§* | 30/225 | 13.3 | PCR | Finland | [115] |
| *A. agrarius* | *Y. pseudotuberculosis‡* | 1/66 | 1.5 | PCR | Poland | [116] |
| *A. agrarius* | *Y. enterocolitica‡* | 21/132 | 15.9 | PCR | Poland | [117] |
| *A. flavicollis* | *Y. enterocolitica‡* | 4/114 | 3.5 | PCR | Poland | [116] |
| *A. agrarius* | *Y. enterocolitica; Y. pseudotuberculosis‡* | 52/8793 | 0.6 | MAT | Ukraine | [118] |
| *A. flavicollis* | *Y. enterocolitica‡* | 13/918 | 1.4 | MAT | Ukraine | [118] |
| *A. uralensis* | *Y. enterocolitica; Y. pseudotuberculosis‡* | 35/2777 | 1.3 | MAT | Ukraine | [118] |
| *A. sylvaticus* | *Y. enterocolitica‡* | 2/75 | 2.7 | PCR | United Kingdom | [119] |
| *A. sylvaticus* | *ns¶* | 3/163 | 1.8 | PCR | United Kingdom | [120] |

| Supplementary Table S5. Viral detection-only records without extractable denominators  Detection-only records comprise positive Apodemus-specific records for which a numerator and/or denominator could not be extracted from the source. These records were retained as qualitative evidence of detection but were not used for prevalence or positivity calculations. Abbreviations: ns, not specified; nr, not reported. | | | | | | |
| --- | --- | --- | --- | --- | --- | --- |
| Pathogen group | Host species | Pathogen(s) | Detection reported | Method | Country | References |
| *Arenaviridae* | *A. sylvaticus* | LCMV / arenavirus | Positive detection reported | IFA | Austria | [82] |
| *Hantaviridae* | *A. flavicollis* | hantavirus / orthohantavirus | Positive detection reported | Indirect immunofluorescence | Austria | [82] |
| *Hantaviridae* | *Apodemus* spp. | hantavirus / orthohantavirus | Positive detection reported | RT-PCR | Czechia | [121] |
| *Hantaviridae* | *A. sylvaticus* | hantavirus / orthohantavirus | Positive detection reported | ELISA | France | [122] |
| *Hantaviridae* | *A. flavicollis* | hantavirus / orthohantavirus | Positive detection reported | RT-PCR | Greece | [123] |
| *Hantaviridae* | *A. sylvaticus* | hantavirus / orthohantavirus | Positive detection reported | ELISA | Netherlands | [12] |
| *Hantaviridae* | *A. agrarius* | hantavirus / orthohantavirus | Positive detection reported | Genetic sequencing | Poland | [124] |
| *Hantaviridae* | *A. agrarius* | hantavirus / orthohantavirus | Positive detection reported | RT-PCR | Serbia | [125] |
| *Hantaviridae* | *Apodemus* spp. | hantavirus / orthohantavirus | Positive detection reported | ELISA | Slovakia | [126] |
| *Picornaviridae* | *Apodemus* spp. | ns | Positive detection reported | Genetic sequencing | United Kingdom | [127] |
| *Poxviridae* | *A. sylvaticus* | Orthopoxvirus / cowpox virus | Positive detection reported | IFA | United Kingdom | [128] |
| *Astroviridae* | *A. flavicollis* | astrovirus | Positive detection reported | Genetic sequencing | Hungary | [129] |
| *Astroviridae* | *A. flavicollis* | astrovirus | Positive detection reported | Genetic sequencing | United Kingdom | [127] |
| *Coronaviridae* | *A. sylvaticus* | coronavirus | Positive detection reported | IFA | Belgium | [130] |
| *Coronaviridae* | *Apodemus* spp. | coronavirus | Positive detection reported | Genetic sequencing | France | [131] |
| *Coronaviridae* | *A. sylvaticus* | coronavirus | Positive detection reported | Genetic sequencing | United Kingdom | [127] |
| *Paramyxoviridae* | *A. flavicollis* | ns | Positive detection reported | Genetic sequencing | United Kingdom | [127] |

| Supplementary Table S6. Bacterial detection-only records without extractable denominators  Detection-only records comprise positive Apodemus-specific records for which a numerator and/or denominator could not be extracted from the source. These records were retained as qualitative evidence of detection but were not used for prevalence or positivity calculations. Abbreviations: ns, not specified; nr, not reported. | | | | | | | |
| --- | --- | --- | --- | --- | --- | --- | --- |
| Pathogen group | **Host species** | **Pathogen(s)** | **Detection reported** | **Method** | **Country** | **References** | |
| *Francisella tularensis* | *A. sylvaticus* | *F. tularensis* | Positive detection reported | PCR | Spain | [132] | |
| *Francisella tularensis* | *A. flavicollis* | *F. tularensis* | Positive detection reported | PCR | Sweden | [133] | |
| *Yersinia* spp. | *A. flavicollis* | ns | Positive detection reported | PCR | Finland | [60] | *Yersinia* spp. not resolved; zoonotic relevance uncertain |
| *Campylobacter* spp. | *A. flavicollis* | ns | Positive detection reported | Genetic sequencing | Slovakia | [134] | |
| *Campylobacter* spp. | *A. sylvaticus* | ns | Positive detection reported | Genetic sequencing | Slovakia | [134] | |
| *Campylobacter* spp. | *A. uralensis* | ns | Positive detection reported | Genetic sequencing | Slovakia | [134] | |
| *Escherichia coli* | *A. flavicollis* | *E. coli* | Positive detection reported | PCR | Czechia | [135] | |
| *Escherichia coli* | *Apodemus* spp. | *E. coli* | Positive detection reported | Culture | Italy | [136] | |
| *Escherichia coli* | *A. agrarius* | *E. coli* | Positive detection reported | WGS | Poland | [137] | |
| *Escherichia coli* | *A. flavicollis* | *E. coli* | Positive detection reported | WGS | Poland | [137] | |
| *Mycobacterium* spp. | *A. sylvaticus* | *M. muris* | Positive detection reported | Culture | United Kingdom | [138] | |
| *Staphylococcus aureus* | *A. flavicollis* | *S. aureus* | Positive detection reported | WGS | Germany | [139] | |
| *Staphylococcus aureus* | *A. flavicollis* | *S. aureus* | Positive detection reported | PCR | Slovakia | [140] | |
| *Staphylococcus aureus* | *A. agrarius* | *S. aureus* | Positive detection reported | PCR | Slovakia | [140] | |
|  |  |  |  |  |  |  | |

References

[1]	D. Duh, S. Hasic, E. Buzan, The impact of illegal waste sites on a transmission of zoonotic viruses, Virol. J. 14 (2017) 134. https://doi.org/10.1186/s12985-017-0798-1.

[2]	J. Laakkonen, E.R. Kallio, H. Kallio-Kokko, O. Vapalahti, A. Vaheri, H. Henttonen, Is there an association of Pneumocystis infection with the presence of arena-, hanta-, and poxvirus antibodies in wild mice and shrews in Finland?, Parasitology 132 (2006) 461–466. https://doi.org/10.1017/S0031182005009315.

[3]	J. Laakkonen, H. Kallio-Kokko, O. Vapalahti, A. Vaheri, M. Vysko?ilov�, P. Munclinger, M. Machol�n, H. Henttonen, The screening of parasites and viral pathogens of small mammals from a farm in southern Finland, and genetic identification of the Finnish house mouse, Mus musculus, Ann. Zool. Fenn. 44 (2007) 202–208.

[4]	C. Mehl, O.A. Adeyemi, C. Wylezich, D. Höper, M. Beer, C. Triebenbacher, G. Heckel, R.G. Ulrich, Lymphocytic Choriomeningitis Virus Lineage V in Wood Mice, Germany, Emerg. Infect. Dis. 30 (2024) 399–401. https://doi.org/10.3201/eid3002.230868.

[5]	V. Tagliapietra, R. Rosà, H.C. Hauffe, J. Laakkonen, L. Voutilainen, O. Vapalahti, A. Vaheri, H. Henttonen, A. Rizzoli, Spatial and Temporal Dynamics of Lymphocytic Choriomeningitis Virus in Wild Rodents, Northern Italy, Emerg. Infect. Dis. (2009). https://doi.org/10.3201/eid1507.081524.

[6]	H. Kallio-Kokko, J. Laakkonen, A. Rizzoli, V. Tagliapietra, I. Cattadori, S.E. Perkins, P.J. Hudson, A. Cristofolini, W. Versini, O. Vapalahti, A. Vaheri, H. Henttonen, Hantavirus and arenavirus antibody prevalence in rodents and humans in Trentino, Northern Italy, Epidemiol. Infect. 134 (2006) 830–836. https://doi.org/10.1017/S0950268805005431.

[7]	J. Ledesma, C.G. Fedele, F. Carro, L. Lledó, M.P. Sánchez-Seco, A. Tenorio, R.C. Soriguer, J.V. Saz, G. Domínguez, M.F. Rosas, J.F. Barandika, M.I. Gegúndez, Independent Lineage of Lymphocytic Choriomeningitis Virus in Wood Mice (Apodemus sylvaticus), Spain, Emerg. Infect. Dis. 15 (2009) 1677–1680. https://doi.org/10.3201/eid1510.090563.

[8]	S. Herrero-Cófreces, F. Mougeot, T. Sironen, H. Meyer, R. Rodríguez-Pastor, J.J. Luque-Larena, Viral Zoonoses in Small Wild Mammals and Detection of Hantavirus, Spain, Emerg. Infect. Dis. 28 (2022) 1294–1296. https://doi.org/10.3201/eid2806.212508.

[9]	K.R. Blasdell, S.D. Becker, J. Hurst, M. Begon, M. Bennett, Host Range and Genetic Diversity of Arenaviruses in Rodents, United Kingdom, Emerg. Infect. Dis. 14 (2008) 1455–1458. https://doi.org/10.3201/eid1409.080209.

[10]	A. Papa, E. Rogozi, E. Velo, E. Papadimitriou, S. Bino, Genetic detection of hantaviruses in rodents, Albania: Hantaviruses in Rodents, Albania, J. Med. Virol. 88 (2016) 1309–1313. https://doi.org/10.1002/jmv.24486.

[11]	K. Jeske, B. Herzig-Straschil, C. Răileanu, D. Kunec, O. Tauchmann, D. Emirhar, S. Schmidt, J. Trimpert, C. Silaghi, G. Heckel, R.G. Ulrich, S. Drewes, Zoonotic pathogen screening of striped field mice (Apodemus agrarius) from Austria, Transbound. Emerg. Dis. 69 (2022) 886–890. https://doi.org/10.1111/tbed.14015.

[12]	P. Heyman, R. Van Mele, F. De Jaegere, J. Klingström, C. Vandenvelde, Å. Lundkvist, F. Rozenfeld, M. Zizi, Distribution of hantavirus foci in Belgium, Acta Trop. 84 (2002) 183–188. https://doi.org/10.1016/S0001-706X(02)00235-8.

[13]	T.G. Chassovnikarova, N.I. Atanassov, I.S. Christova, H.A. Dimitrov, V.I. Mitkovska, I.P. Trifonova, T.K. Gladnishka, N.Z. Kalvatchev, E. Mohareb, Hantavirus Infections in Host Populations of Yellow-Necked and Field Mice (Rodentia: Muridae) in South Bulgaria, ACTA Zool. Bulg. 65 (2013) 397–402.

[14]	I. Christova, A. Plyusnina, T. Gladnishka, N. Kalvatchev, I. Trifonova, H. Dimitrov, V. Mitkovska, E. Mohareb, A. Plyusnin, Detection of Dobrava hantavirus RNA in Apodemus mice in Bulgaria, J. Med. Virol. 87 (2015) 263–268. https://doi.org/10.1002/jmv.24033.

[15]	V. Németh, M. Madai, A. Maráczi, B. Bérczi, G. Horváth, M. Oldal, P. Kisfali, K. Bányai, F. Jakab, Detection of Dobrava-Belgrade hantavirus using recombinant-nucleocapsid-based enzyme-linked immunosorbent assay and SYBR Green-based real-time reverse transcriptase-polymerase chain reaction, Arch. Virol. 156 (2011) 1655–1660. https://doi.org/10.1007/s00705-011-1013-0.

[16]	A. Plyusnina, L.C. Krajinović, J. Margaletić, J. Niemimaa, K. Nemirov, Å. Lundkvist, A. Markotić, M. Miletić-Medved, T. Avšič-Županc, H. Henttonen, A. Plyusnin, Genetic evidence for the presence of two distinct hantaviruses associated with Apodemus mice in Croatia and analysis of local strains, J. Med. Virol. 83 (2011) 108–114. https://doi.org/10.1002/jmv.21929.

[17]	P. Svoboda, G. Dobler, A. Markotić, I.-C. Kurolt, S. Speck, J. Habuš, M. Vucelja, L.C. Krajinović, A. Tadin, J. Margaletić, S. Essbauer, Survey for Hantaviruses, Tick-Borne Encephalitis Virus, and *Rickettsia* spp. in Small Rodents in Croatia, Vector-Borne Zoonotic Dis. 14 (2014) 523–530. https://doi.org/10.1089/vbz.2013.1457.

[18]	A. Tadin, N. Turk, M. Korva, J. Margaletić, R. Beck, M. Vucelja, J. Habuš, P. Svoboda, T.A. Županc, H. Henttonen, A. Markotić, Multiple Co-infections of Rodents with Hantaviruses, *Leptospira,* and *Babesia* in Croatia, Vector-Borne Zoonotic Dis. 12 (2012) 388–392. https://doi.org/10.1089/vbz.2011.0632.

[19]	A. Tadin, L. Bjedov, J. Margaletic, B. Zibrat, L.C. Krajinovic, P. Svoboda, I.C. Kurolt, Z.S. Majetic, N. Turk, O.D. Rode, R. Civljak, I. Kuzman, A. Markotic, High infection rate of bank voles ( *Myodes glareolus* ) with Puumala virus is associated with a winter outbreak of haemorrhagic fever with renal syndrome in Croatia, Epidemiol. Infect. 142 (2014) 1945–1951. https://doi.org/10.1017/S095026881300321X.

[20]	A. Tadin, R. Tokarz, A. Markotić, J. Margaletić, N. Turk, J. Habuš, P. Svoboda, M. Vucelja, A. Desai, K. Jain, W. Ian Lipkin, Molecular Survey of Zoonotic Agents in Rodents and Other Small Mammals in Croatia, Am. J. Trop. Med. Hyg. 94 (2016) 466–473. https://doi.org/10.4269/ajtmh.15-0517.

[21]	V. Hönig, J. Kamiš, A. Maršíková, T. Matějková, P. Stopka, A. Mácová, D. Růžek, J. Kvičerová, Orthohantaviruses in Reservoir and Atypical Hosts in the Czech Republic: Spillover Infection and Indication of Virus-Specific Tissue Tropism, Microbiol. Spectr. 10 (2022) e01306-22. https://doi.org/10.1128/spectrum.01306-22.

[22]	M. Weidmann, P. Schmidt, M. Vackova, K. Krivanec, P. Munclinger, F.T. Hufert, Identification of genetic evidence for dobrava virus spillover in rodents by nested reverse transcription (RT)-PCR and TaqMan RT-PCR, J. Clin. Microbiol. 43 (2005) 808–812. https://doi.org/10.1128/jcm.43.2.808-812.2005.

[23]	K. Nemirov, H.K. Andersen, H. Leirs, H. Henttonen, A. Vaheri, Å. Lundkvist, A. Plyusnin, Saaremaa hantavirus in Denmark, J. Clin. Virol. 30 (2004) 254–257. https://doi.org/10.1016/j.jcv.2003.12.009.

[24]	I. Golovljova, K. Brus Sjölander, G. Lindegren, S. Vene, V. Vasilenko, A. Plyusnin, Å. Lundkvist, Hantaviruses in Estonia, J. Med. Virol. 68 (2002) 589–598. https://doi.org/10.1002/jmv.10231.

[25]	J.L. Abbate, M. Galan, M. Razzauti, T. Sironen, L. Voutilainen, H. Henttonen, P. Gasqui, J.-F. Cosson, N. Charbonnel, Pathogen community composition and co-infection patterns in a wild community of rodents, Peer Community J. 4 (2024). https://doi.org/10.24072/pcjournal.370.

[26]	M. Schlegel, B. Klempa, B. Auste, M. Bemmann, J. Schmidt-Chanasit, T. Büchner, M.H. Groschup, M. Meier, A. Balkema-Buschmann, H. Zoller, D.H. Krüger, R.G. Ulrich, Dobrava-Belgrade Virus Spillover Infections, Germany, Emerg. Infect. Dis. 15 (2009) 2017–2020. https://doi.org/10.3201/eid1512.090923.

[27]	F. Jakab, G. Horváth, E. Ferenczi, J. Sebők, Z. Varecza, G. Szűcs, Detection of Dobrava hantaviruses in *Apodemus agrarius* mice in the Transdanubian region of Hungary, Virus Res. 128 (2007) 149–152. https://doi.org/10.1016/j.virusres.2007.04.015.

[28]	K. Kurucz, M. Madai, D. Bali, D. Hederics, G. Horváth, G. Kemenesi, F. Jakab, Parallel Survey of Two Widespread Renal Syndrome-Causing Zoonoses: Leptospira spp. and Hantavirus in Urban Environment, Hungary, Vector-Borne Zoonotic Dis. 18 (2018) 200–205. https://doi.org/10.1089/vbz.2017.2204.

[29]	M. Madai, V. Németh, M. Oldal, G. Horváth, R. Herczeg, K. Kelemen, G. Kemenesi, F. Jakab, Temporal Dynamics of Two Pathogenic Hantaviruses Among Rodents in Hungary, Vector-Borne Zoonotic Dis. 20 (2020) 212–221. https://doi.org/10.1089/vbz.2019.2438.

[30]	A. Plyusnina, E. Ferenczi, G.R. Rácz, K. Nemirov, Å. Lundkvist, A. Vaheri, O. Vapalahti, A. Plyusnin, Co-circulation of three pathogenic hantaviruses: Puumala, Dobrava, and Saaremaa in Hungary, J. Med. Virol. 81 (2009) 2045–2052. https://doi.org/10.1002/jmv.21635.

[31]	S. Leopardi, P. Drzewnioková, M. Baggieri, A. Marchi, P. Bucci, M. Bregoli, P. De Benedictis, F. Gobbo, L. Bellinati, C. Citterio, I. Monne, A. Pastori, G. Zamperin, E. Palumbo, F. Festa, M. Castellan, M. Zorzan, E. D’Ugo, P. Zucca, C. Terregino, F. Magurano, Identification of Dobrava-Belgrade Virus in Apodemus flavicollis from North-Eastern Italy during Enhanced Mortality, Viruses 14 (2022) 1241. https://doi.org/10.3390/v14061241.

[32]	A. Rizzoli, V. Tagliapietra, R. Rosà, H.C. Hauffe, G. Marini, L. Voutilainen, T. Sironen, C. Rossi, D. Arnoldi, H. Henttonen, Recent increase in prevalence of antibodies to Dobrava-Belgrade virus (DOBV) in yellow-necked mice in northern Italy, Epidemiol. Infect. 143 (2015) 2241–2244. https://doi.org/10.1017/S0950268814003525.

[33]	K. Jeske, J. Schulz, D. Tekemen, L. Balčiauskas, L. Balčiauskienė, M. Hiltbrunner, S. Drewes, A. Mayer-Scholl, G. Heckel, R.G. Ulrich, Cocirculation of Leptospira spp. and multiple orthohantaviruses in rodents, Lithuania, Northern Europe, Transbound. Emerg. Dis. 69 (2022) e3196–e3201. https://doi.org/10.1111/tbed.14470.

[34]	A. Michalski, M. Niemcewicz, A. Bielawska-Drózd, A. Nowakowska, J. Gaweł, G. Pitucha, J. Joniec, K. Zielonka, A. Marciniak-Niemcewicz, J. Kocik, Surveillance of Hantaviruses in Poland: A Study of Animal Reservoirs and Human Hantavirus Disease in Subcarpathia, Vector Borne Zoonotic Dis. 14 (2014) 514–522. https://doi.org/10.1089/vbz.2013.1468.

[35]	S.-H. Lee, J.S. No, W.-K. Kim, E. Gajda, A. Perec-Matysiak, J.-A. Kim, J. Hildebrand, R. Yanagihara, J.-W. Song, Molecular Epidemiology and Genetic Diversity of Orthohantaviruses in Small Mammals in Western Poland, Am. J. Trop. Med. Hyg. 103 (2020) 193–199. https://doi.org/10.4269/ajtmh.19-0802.

[36]	M. Sadkowska-Todys, D. Dudek-Godeau, S. Kamińska, A. Baumann-Popczyk, M. Czerwiński, B. Kucharczyk, A. Zieliński, Occurrence and maintenance of hantavirus infections among rodent populations in their natural habitat--results of a field study from Podkarpackie province, Poland 2010-2012, Przegl. Epidemiol. 69 (2015) 283–288, 395–399.

[37]	R.I. Panculescu-Gatej, A. Sirbu, S. Dinu, M. Waldstrom, P. Heyman, D. Murariu, A. Petrescu, C. Szmal, G. Oprisan, A. Lundkvist, C.S. Ceianu, Dobrava virus carried by the yellow-necked field mouse Apodemus flavicollis, causing hemorrhagic fever with renal syndrome in Romania, Vector Borne Zoonotic Dis. Larchmt. N 14 (2014) 358–364. https://doi.org/10.1089/vbz.2013.1400.

[38]	A. Andreychev, E. Boyarova, O. Brandler, A. Tukhbatullin, S. Kapustina, Terrestrial and Subterranean Mammals as Reservoirs of Zoonotic Diseases in the Central Part of European Russia, Diversity 15 (2023) 39. https://doi.org/10.3390/d15010039.

[39]	M. Maydanov, A. Andreychev, E. Boyarova, V. Kuznetsov, E. Ilykaeva, Small mammals as reservoirs of tularemia and hfrs in the forest zone of saransk, in: Ideas, 2021: pp. 128–135.

[40]	H. Kariwa, E.A. Tkachenko, V.G. Morozov, T. Seto, Y. Tanikawa, S.I. Kolominov, S.N. Belov, I. Nakamura, N. Hashimoto, A.E. Balakiev, T.K. Dzagurnova, N.H. bin A. Daud, D. Miyashita, O.A. Medvedkina, M. Nakauchi, M. Ishizuka, K. Yoshii, K. Yoshimatsu, J. Arikawa, I. Takashima, Epidemiological Study of Hantavirus Infection in the Samara Region of European Russia, J. Vet. Med. Sci. 71 (2009) 1569–1578. https://doi.org/10.1292/jvms.001569.

[41]	G. Stamenković, V. Nikolić, J. Blagojević, V. Bugarski-Stanojević, T. Adnađević, M. Stanojević, M. Vujošević, Genetic Analysis of Dobrava–Belgrade Virus from Western Serbia – A Newly Detected Focus in the Balkan Peninsula, Zoonoses Public Health 62 (2015) 141–150. https://doi.org/10.1111/zph.12136.

[42]	B. Klempa, M. Stanko, M. Labuda, R. Ulrich, H. Meisel, D.H. Krüger, Central European Dobrava Hantavirus Isolate from a Striped Field Mouse (Apodemus agrarius), J. Clin. Microbiol. 43 (2005) 2756–2763. https://doi.org/10.1128/jcm.43.6.2756-2763.2005.

[43]	C. Sibold, R. Ulrich, M. Labuda, Å. Lundkvist, H. Martens, M. Schütt, P. Gerke, K. Leitmeyer, H. Meisel, D.H. Krüger, Dobrava hantavirus causes hemorrhagic fever with renal syndrome in central Europe and is carried by two different Apodemus mice species, J. Med. Virol. 63 (2001) 158–167. https://doi.org/10.1002/1096-9071(20000201)63:2%3C158::AID-JMV1011%3E3.0.CO;2-%23.

[44]	T. Avsic-Zupanc, K. Nemirov, M. Petrovec, T. Trilar, M. Poljak, A. Vaheri, A. Plyusnin, Genetic analysis of wild-type Dobrava hantavirus in Slovenia: co-existence of two distinct genetic lineages within the same natural focus, J. Gen. Virol. 81 (2000) 1747–1755. https://doi.org/10.1099/0022-1317-81-7-1747.

[45]	M. Lõhmus, J. Verner-Carlsson, O. Borg, A. Albihn, Å. Lundkvist, Hantavirus in new geographic regions, Sweden, Infect. Ecol. Epidemiol. 6 (2016) 31465. https://doi.org/10.3402/iee.v6.31465.

[46]	I.V. Demchyshyna, G.E. Glass, O.A. Hluzd, V.V. Kutseva, M.K. Taylor, E.P. Williams, V. Kurpita, C.B. Jonsson, Cocirculation of Two Orthohantavirus Species in Small Mammals of the Northwestern Ukraine, J. Wildl. Dis. 56 (2020) 640–645. https://doi.org/10.7589/2019-09-238.

[47]	E.P. Williams, M.K. Taylor, I. Demchyshyna, I. Nebogatkin, O. Nesterova, I. Khuda, L. Chernenko, O.A. Hluzd, V.V. Kutseva, G.E. Glass, N. Yanko, C.B. Jonsson, Prevalence of Hantaviruses Harbored by Murid Rodents in Northwestern Ukraine and Discovery of a Novel Puumala Virus Strain, Viruses 13 (2021) 1640. https://doi.org/10.3390/v13081640.

[48]	M. Horemans, J. Van Bets, T. Joly Maes, P. Maes, B. Vanmechelen, Discovery and genome characterization of six new orthoparamyxoviruses in small Belgian mammals, Virus Evol. 9 (2023) vead065. https://doi.org/10.1093/ve/vead065.

[49]	B. Vanmechelen, S. Meurs, M. Horemans, A. Loosen, T. Joly Maes, L. Laenen, V. Vergote, F.R. Koundouno, N. Magassouba, M.K. Konde, I.S. Condé, M.W. Carroll, P. Maes, The characterization of multiple novel paramyxoviruses highlights the diverse nature of the subfamily Orthoparamyxovirinae, Virus Evol. 8 (2022) veac061. https://doi.org/10.1093/ve/veac061.

[50]	W. Ghawar, H. Pascalis, J. Bettaieb, J. Mélade, A. Gharbi, M.A. Snoussi, D. Laouini, S.M. Goodman, A. Ben Salah, K. Dellagi, Insight into the global evolution of Rodentia associated Morbilli-related paramyxoviruses, Sci. Rep. 7 (2017) 1974. https://doi.org/10.1038/s41598-017-02206-0.

[51]	R. Kallies, Ljungan virus–prevalence in rodents and virus pathogenesis, PhD Thesis, Freie Universität Berlin, 2010.

[52]	C. Billinis, Encephalomyocarditis virus infection in wildlife species in Greece., J. Wildl. Dis. 45 (2009) 522–526.

[53]	C. Fevola, C. Rossi, F. Rosso, M. Girardi, R. Rosà, M. Manica, L. Delucchi, D. Rocchini, C.X. Garzon-Lopez, D. Arnoldi, A. Bianchi, E. Buzan, N. Charbonnel, M. Collini, L. Ďureje, F. Ecke, N. Ferrari, S. Fischer, E.L. Gillingham, B. Hörnfeldt, M. Kazimírová, A. Konečný, M. Maas, M. Magnusson, A. Miller, J. Niemimaa, Å. Nordström, A. Obiegala, G. Olsson, P. Pedrini, J. Piálek, C.B. Reusken, F. Rizzolli, C. Romeo, C. Silaghi, T. Sironen, M. Stanko, V. Tagliapietra, R.G. Ulrich, O. Vapalahti, L. Voutilainen, L. Wauters, A. Rizzoli, A. Vaheri, A.J. Jääskeläinen, H. Henttonen, H.C. Hauffe, Geographical Distribution of Ljungan Virus in Small Mammals in Europe, Vector-Borne Zoonotic Dis. 20 (2020) 692–702. https://doi.org/10.1089/vbz.2019.2542.

[54]	H.C. Hauffe, B. Niklasson, T. Olsson, A. Bianchi, A. Rizzoli, W. Klitz, Ljungan Virus Detected in Bank Voles (Myodes glareolus) and Yellow-Necked Mice (Apodemus flavicollis) from Northern Italy, J. Wildl. Dis. 46 (2010) 262–266. https://doi.org/10.7589/0090-3558-46.1.262.

[55]	A.-M. Salisbury, M. Begon, W. Dove, B. Niklasson, J.P. Stewart, Ljungan virus is endemic in rodents in the UK, Arch. Virol. 159 (2014) 547–551. https://doi.org/10.1007/s00705-013-1731-6.

[56]	S. Essbauer, S. Hartnack, K. Misztela, J. Kießling-Tsalos, W. Bäumler, M. Pfeffer, Patterns of Orthopox Virus Wild Rodent Hosts in South Germany, Vector-Borne Zoonotic Dis. 9 (2009) 301–311. https://doi.org/10.1089/vbz.2008.0205.

[57]	P.M. Kinnunen, H. Henttonen, B. Hoffmann, E.R. Kallio, C. Korthase, J. Laakkonen, J. Niemimaa, A. Palva, M. Schlegel, H.S. Ali, P. Suominen, R.G. Ulrich, A. Vaheri, O. Vapalahti, Orthopox Virus Infections in Eurasian Wild Rodents, Vector-Borne Zoonotic Dis. 11 (2011) 1133–1140. https://doi.org/10.1089/vbz.2010.0170.

[58]	M. Oldal, T. Sironen, H. Henttonen, O. Vapalahti, M. Madai, G. Horváth, B. Dallos, A. Kutas, F. Földes, G. Kemenesi, V. Németh, K. Bányai, F. Jakab, Serologic Survey of Orthopoxvirus Infection Among Rodents in Hungary, Vector-Borne Zoonotic Dis. 15 (2015) 317–322. https://doi.org/10.1089/vbz.2014.1731.

[59]	S. Olkkola, M. Rossi, A. Jaakkonen, M. Simola, J. Tikkanen, M. Hakkinen, P. Tuominen, O. Huitu, J. Niemimaa, H. Henttonen, R. Kivistö, Host-Dependent Clustering of Campylobacter Strains From Small Mammals in Finland, Front. Microbiol. 11 (2021). https://doi.org/10.3389/fmicb.2020.621490.

[60]	M. Simola, S. Hallanvuo, H. Henttonen, O. Huitu, J. Niemimaa, H. Rossow, L. Seppä-Lassila, J. Ranta, Small mammals as carriers of zoonotic bacteria on pig and cattle farms – Prevalence and risk of exposure in an integrative approach, Prev. Vet. Med. 229 (2024) 106228. https://doi.org/10.1016/j.prevetmed.2024.106228.

[61]	M. Ylänen, Campylobacter spp. in Finnish small mammals, (2020).

[62]	A. Backhans, M. Jacobson, I. Hansson, M. Lebbad, S.T. Lambertz, E. Gammelgård, M. Saager, O. Akande, C. Fellström, Occurrence of pathogens in wild rodents caught on Swedish pig and chicken farms, Epidemiol. Infect. 141 (2013) 1885–1891. https://doi.org/10.1017/S0950268812002609.

[63]	M. Lõhmus, A. Albihn, Gastrointestinal Pathogens in Rodents Overwintering in Human Facilities around Uppsala, Sweden, J. Wildl. Dis. 49 (2013) 747–749. https://doi.org/10.7589/2013-02-028.

[64]	I.M. Krijger, B.G. Meerburg, C. Harmanus, S.A. Burt, Clostridium difficile in wild rodents and insectivores in the Netherlands, Lett. Appl. Microbiol. 69 (2019) 35–40. https://doi.org/10.1111/lam.13159.

[65]	S.A. Burt, K. Meijer, P. Burggraaff, W.S. Kamerich, C. Harmanus, Wild mice in and around the city of Utrecht, the Netherlands, are carriers of Clostridium difficile but not ESBL‐producing Enterobacteriaceae, Salmonella spp. or MRSA, Lett. Appl. Microbiol. 67 (2018) 513–519. https://doi.org/10.1111/lam.13066.

[66]	S. Andrés‐Lasheras, R. Bolea, R.C. Mainar‐Jaime, E. Kuijper, E. Sevilla, I. Martín‐Burriel, M. Chirino‐Trejo, Presence of Clostridium difficile in pig faecal samples and wild animal species associated with pig farms, J. Appl. Microbiol. 122 (2017) 462–472. https://doi.org/10.1111/jam.13343.

[67]	Y. Ilieva, M.M. Zaharieva, L. Dimitrova, M.D. Kaleva, J. Jordanova, M. Dimitrova, M. Beltcheva, I. Aleksieva, Y. Georgiev, Y. Manasiev, H. Najdenski, Preliminary Data on Escherichia coli, Yersinia enterocolitica, and Other Bacteria, as Well as Absent African Swine Fever Virus in the Gut Microbiota of Wild Mice and Voles from Bulgaria, Microbiol. Res. 14 (2023) 1788–1819. https://doi.org/10.3390/microbiolres14040123.

[68]	M. Vittecoq, E. Elguero, L. Brazier, N. Renaud, T. Blanchon, F. Roux, F. Renaud, P. Durand, F. Thomas, Antimicrobial-Resistant Bacteria Carriage in Rodents According to Habitat Anthropization, EcoHealth 20 (2023) 84–92. https://doi.org/10.1007/s10393-023-01638-7.

[69]	S. Guenther, M. Grobbel, K. Heidemanns, M. Schlegel, R.G. Ulrich, C. Ewers, L.H. Wieler, First insights into antimicrobial resistance among faecal *Escherichia coli* isolates from small wild mammals in rural areas, Sci. Total Environ. 408 (2010) 3519–3522. https://doi.org/10.1016/j.scitotenv.2010.05.005.

[70]	M.P. de Cock, V.O. Baede, H.J. Esser, M. Fonville, A. de Vries, W.F. de Boer, C. Mehl, R.G. Ulrich, G. Schares, R.W. Hakze-van der Honing, W.H.M. van der Poel, H. Sprong, M. Maas, T(r)icky Environments: Higher Prevalence of Tick-Borne Zoonotic Pathogens in Rodents from Natural Areas Compared with Urban Areas, Vector-Borne Zoonotic Dis. 24 (2024) 478–488. https://doi.org/10.1089/vbz.2023.0151.

[71]	C.A. Alonso, D. González-Barrio, C. Tenorio, F. Ruiz-Fons, C. Torres, Antimicrobial resistance in faecal *Escherichia coli* isolates from farmed red deer and wild small mammals. Detection of a multiresistant *E. coli* producing extended-spectrum beta-lactamase, Comp. Immunol. Microbiol. Infect. Dis. 45 (2016) 34–39. https://doi.org/10.1016/j.cimid.2016.02.003.

[72]	F. Occhibove, N.J. McKeown, C. Risley, J.E. Ironside, Eco-epidemiological screening of multi-host wild rodent communities in the UK reveals pathogen strains of zoonotic interest, Int. J. Parasitol. Parasites Wildl. 17 (2022) 278–287. https://doi.org/10.1016/j.ijppaw.2022.02.010.

[73]	N.J. Williams, C. Sherlock, T.R. Jones, H.E. Clough, S.E. Telfer, M. Begon, N. French, C.A. Hart, M. Bennett, The prevalence of antimicrobial‐resistant Escherichia coli in sympatric wild rodents varies by season and host, J. Appl. Microbiol. 110 (2011) 962–970. https://doi.org/10.1111/j.1365-2672.2011.04952.x.

[74]	M. Mihelčić, J. Habuš, M. Vucelja, P. Svoboda, I.-C. Kurolt, A. Markotić, N. Turk, J. Margaletić, M. Šantić, Prevalence of *Francisella tularensis* in the population of small mammals species in continental forests of Croatia, Šumar. List 142 (2018) 481–485. https://doi.org/10.31298/sl.142.9-10.3.

[75]	E. Bártová, H.L. Kučerová, A. Žákovská, M. Budíková, H. Nejezchlebová, *Coxiella burnetii* and *Francisella tularensis* in wild small mammals from the Czech Republic, Ticks Tick-Borne Dis. 11 (2020) 101350. https://doi.org/10.1016/j.ttbdis.2019.101350.

[76]	P. Pittermannová, A. Žákovská, P. Váňa, J. Marková, F. Treml, L. Černíková, M. Budíková, E. Bártová, Wild Small Mammals and Ticks in Zoos—Reservoir of Agents with Zoonotic Potential?, Pathogens 10 (2021) 777. https://doi.org/10.3390/pathogens10060777.

[77]	A. Žákovská, E. Bártová, P. Pittermannová, M. Budíková, Antibodies Related to Borrelia burgdorferi sensu lato, Coxiella burnetii, and Francisella tularensis Detected in Serum and Heart Rinses of Wild Small Mammals in the Czech Republic, Pathogens 10 (2021) 419. https://doi.org/10.3390/pathogens10040419.

[78]	P. Kaysser, E. Seibold, K. Mätz-Rensing, M. Pfeffer, S. Essbauer, W.D. Splettstoesser, Re-emergence of tularemia in Germany: Presence of Francisella tularensis in different rodent species in endemic areas, BMC Infect. Dis. 8 (2008) 157. https://doi.org/10.1186/1471-2334-8-157.

[79]	O.B. Zlenko, G.E. Tkach, A.B. Sukhorukova, L.V. Kylypko, L.S. Machota, O.S. Ignatenkov, K.V. Vinokurova, G.R. Shamychkova, O.P. Shtepa, V.G. Rezvykh, J. Schwarz, A. Duerr, C. Popp, H. von Buttlar, R. Wolfel, O.S. Solodiankin, A.P. Gerilovych, PCR Based Prevalence Study of Francisella Tularensis in Kharkiv, Dnipropetrovsk, and Mykolaiv Oblasts during 2015–2018, J. Vet. Res. 64 (2020) 63–71. https://doi.org/10.2478/jvetres-2020-0007.

[80]	T. Radovanović, G. Jokić, T. Blažić, G. Veinović, S. Radojičić, N. Stević, Presence of Francisella tularensis in Apodemus spp. on the Edges of Forest Areas in Belgrade, Serbia, Integr. Zool. n/a (2026). https://doi.org/10.1111/1749-4877.12975.

[81]	I. Lopes de Carvalho, A. Toledo, C.L. Carvalho, J.F. Barandika, L.B. Respicio-Kingry, C. Garcia-Amil, A.L. García-Pérez, A.S. Olmeda, L. Zé-Zé, J.M. Petersen, P. Anda, M.S. Núncio, R. Escudero, *Francisella* species in ticks and animals, Iberian Peninsula, Ticks Tick-Borne Dis. 7 (2016) 159–165. https://doi.org/10.1016/j.ttbdis.2015.10.009.

[82]	S. Schmidt, S.S. Essbauer, A. Mayer-Scholl, S. Poppert, J. Schmidt-Chanasit, B. Klempa, K. Henning, G. Schares, M.H. Groschup, F. Spitzenberger, D. Richter, G. Heckel, R.G. Ulrich, Multiple Infections of Rodents with Zoonotic Pathogens in Austria, Vector-Borne Zoonotic Dis. 14 (2014) 467–475. https://doi.org/10.1089/vbz.2013.1504.

[83]	E. Taseva, I. Christova, E. Panayotova, I. Trifonova, Investigation of murine rodents for the presence of Leptospira DNA by nested PCR, Probl. Infect. Parasit. Dis. 47 (2019) 25–29. https://doi.org/10.58395/pipd.v47i1.14.

[84]	J. Margaletić, M. Glavaš, N. Turk, Z. Milas, V. Starešina, Small rodents reservoires of leptospiroses in the forests of Posavina in Croatia., Glas. Za Šumske Pokuse 39 (2002) 43–65.

[85]	Z. Milas, N. Turk, V. Staresina, J. Margaletic, A. Slavica, D. Zivkovic, Z. Modric, The role of myomorphous mammals as reservoirs of leptospira in the pedunculate oak forests of Croatia, Vet. Arh. 72 (2002) 119–130.

[86]	Z. Milas, Z. Majetić, J. Habuš, V. Perko, V. Starešina, L. Barbić, V. Stevanović, M. Perharić, B. Ljubić, N. Turk, The occurrence and maintenance of Leptospira serovars Australis and Bratislava in domestic and wild animals in Croatia., Vet. Arh. 83 (2013) 357–369.

[87]	Z. Stritof Majetic, R. Galloway, E. Ruzic Sabljic, Z. Milas, V. Mojcec Perko, J. Habus, J. Margaletic, R. Pernar, N. Turk, Epizootiological survey of small mammals as *Leptospira* spp. reservoirs in Eastern Croatia, Acta Trop. 131 (2014) 111–116. https://doi.org/10.1016/j.actatropica.2013.12.009.

[88]	A. Balážová, E. Nosková, P. Široký, C. Durrant, V. Baláž, Diversity and dynamics of zoonotic pathogens within a local community of small mammals, Biologia (Bratisl.) 76 (2021) 3267–3273. https://doi.org/10.1007/s11756-021-00797-8.

[89]	A. Žákovská, F. Treml, H. Nejezchlebová, J. Nepeřený, M. Budíková, E. Bártová, Leptospira interrogans Sensu Lato in Wild Small Mammals in Three Moravian Localities of the Czech Republic, Pathogens 11 (2022) 888. https://doi.org/10.3390/pathogens11080888.

[90]	F. Treml, M. Pejcoch, Z. Holesovska, Small mammals-natural reservoir of pathogenic leptospires, Vet. Med.-PRAHA- 47 (2002) 309–314.

[91]	F. Treml, J. Nepeřený, E. Jánová, H. Banďouchová, J. Pikula, Prevalence of antibodies against leptospires in small mammals in relation to age, sex and season, Acta Vet. Brno 81 (2012) 97–102. https://doi.org/10.2754/avb201281020097.

[92]	M. Garcia-Lopez, T. Lurier, M. Bouilloud, J. Pradel, C. Tatard, D. Sepulveda, G. Anfray, J. Dussert, P. Bourhy, N. Charbonnel, Z. Djelouadji, Prevalence, genetic diversity and eco-epidemiology of pathogenic Leptospira species in small mammal communities in urban parks Lyon city, France, PLOS ONE 19 (2024) e0300523. https://doi.org/10.1371/journal.pone.0300523.

[93]	E. Izquierdo-Rodríguez, Á. Fernández-Álvarez, N. Martín-Carrillo, B. Marchand, C. Feliu, J. Miquel, P. Foronda, Y. Quilichini, Pathogenic Leptospira species in rodents from Corsica (France), PLOS ONE 15 (2020) e0233776. https://doi.org/10.1371/journal.pone.0233776.

[94]	S. Fischer, A. Mayer-Scholl, C. Imholt, N.G. Spierling, E. Heuser, S. Schmidt, D. Reil, U.M. Rosenfeld, J. Jacob, K. Nöckler, R.G. Ulrich, Leptospira Genomospecies and Sequence Type Prevalence in Small Mammal Populations in Germany, Vector-Borne Zoonotic Dis. 18 (2018) 188–199. https://doi.org/10.1089/vbz.2017.2140.

[95]	K. Jeske, J. Jacob, S. Drewes, M. Pfeffer, G. Heckel, R.G. Ulrich, C. Imholt, Hantavirus– *Leptospira* coinfections in small mammals from central Germany, Epidemiol. Infect. 149 (2021) e97. https://doi.org/10.1017/S0950268821000443.

[96]	A. Mayer-Scholl, J.A. Hammerl, S. Schmidt, R.G. Ulrich, M. Pfeffer, D. Woll, H.C. Scholz, A. Thomas, K. Nöckler, Leptospira spp. in Rodents and Shrews in Germany, Int. J. Environ. Res. Public. Health 11 (2014) 7562–7574. https://doi.org/10.3390/ijerph110807562.

[97]	A. Obiegala, D. Woll, C. Karnath, C. Silaghi, S. Schex, S. Eßbauer, M. Pfeffer, Prevalence and Genotype Allocation of Pathogenic Leptospira Species in Small Mammals from Various Habitat Types in Germany, PLoS Negl. Trop. Dis. 10 (2016) e0004501. https://doi.org/10.1371/journal.pntd.0004501.

[98]	A. Obiegala, C. Albrecht, M. Dafalla, S. Drewes, C. Oltersdorf, H. Turni, C. Imholt, J. Jacob, C. Wagner-Wiening, R.G. Ulrich, M. Pfeffer, Leptospira spp. in Small Mammals from Areas with Low and High Human Hantavirus Incidences in South-West Germany, Vector-Borne Zoonotic Dis. 17 (2017) 312–318. https://doi.org/10.1089/vbz.2016.2036.

[99]	M. Vitale, S. Agnello, M. Chetta, B. Amato, G. Vitale, C.D. Bella, D. Vicari, V.D.M.L. Presti, Human leptospirosis cases in Palermo Italy. The role of rodents and climate, J. Infect. Public Health 11 (2018) 209–214. https://doi.org/10.1016/j.jiph.2017.07.024.

[100]	I. Piredda, M.N. Ponti, B. Palmas, M. Noworol, A. Pedditzi, L. Rebechesu, V. Chisu, Molecular Typing of Pathogenic Leptospira Species Isolated from Wild Mammal Reservoirs in Sardinia, Animals 11 (2021) 1109. https://doi.org/10.3390/ani11041109.

[101]	V. Burlacu, Recent evolutions of natural foci of leptospirosis and small mammal communities (rodentia, insectivora) in the Republic of Moldova, One Health Risk Manag. 2 (2020) 23–33.

[102]	S. Gheorghiţa, V. Kiku, V. Nistreanu, V. Burlacu, A. Guţu, V. Melnic, E. Culibacinaia, O. Beneş, The role of micromammals in the maintenance of leptospirosis foci in the Republic of Moldova, Olten.-Stud. Si Comun. Stiintele Nat. (2009) 291–296.

[103]	I.M. Krijger, A.A.A. Ahmed, M.G.A. Goris, J.B.W.J. Cornelissen, P.W.G. Groot Koerkamp, B.G. Meerburg, Wild rodents and insectivores as carriers of pathogenic Leptospira and Toxoplasma gondii in The Netherlands, Vet. Med. Sci. 6 (2020) 623–630. https://doi.org/10.1002/vms3.255.

[104]	A. Wójcik-Fatla, V. Zajac, J. Sroka, M. Piskorski, E. Cisak, J. Dutkiewicz, A small scale survey of Leptospira in mammals from eastern Poland, Ann. Agric. Environ. Med. 20 (2013).

[105]	J. Blagojević, M. Šekler, M. Rajičić, B. Pejić, I. Budinski, V.M. Jovanović, T. Adnađević, D. Vidanović, K. Matović, M. Vujošević, The prevalence of pathogenic forms of Leptospira in natural populations of small wild mammals in Serbia, (2019). https://doi.org/10.1556/004.2019.035.

[106]	J. Millán, A. Cevidanes, A.D. Chirife, M.G. Candela, L. León-Vizcaíno, Risk factors of Leptospira infection in Mediterranean periurban micromammals, Zoonoses Public Health 65 (2018) e79–e85. https://doi.org/10.1111/zph.12411.

[107]	H. Adler, S. Vonstein, P. Deplazes, C. Stieger, R. Frei, Prevalence of Leptospira spp. in various species of small mammals caught in an inner-city area in Switzerland, Epidemiol. Infect. 128 (2002) 107–109. https://doi.org/10.1017/S0950268801006380.

[108]	O. Markovych, V. Tymchyk, I. Kolesnikova, Leptospirosis in Zakarpattia Oblast (2005-2015)., Vector Borne Zoonotic Dis. Larchmt. N 19 (2019) 333–340. https://doi.org/10.1089/vbz.2018.2356.

[109]	O. Zubach, O. Semenyshyn, L. Hatsji, M. Demchyshyn, A. Zinchuk, Leptospira interrogans in mammals in Lviv Oblast, Ukraine, 2001-2015, PLoS Negl. Trop. Dis. 13 (2019) e0007793. https://doi.org/10.1371/journal.pntd.0007793.

[110]	O. Fischer, L. Mátlová, J. Bartl, L. Dvorská, I. Melichárek, I. Pavlík, Findings of mycobacteria in insectivores and small rodents, Folia Microbiol. (Praha) 45 (2000) 147–152. https://doi.org/10.1007/BF02817414.

[111]	V. Tagliapietra, M.B. Boniotti, A. Mangeli, I. Karaman, G. Alborali, M. Chiari, M. D’Incau, M. Zanoni, A. Rizzoli, M.L. Pacciarini, Mycobacterium microti at the Environment and Wildlife Interface, Microorganisms 9 (2021) 2084. https://doi.org/10.3390/microorganisms9102084.

[112]	L. Varela-Castro, O. Torrontegi, I.A. Sevilla, M. Barral, Detection of Wood Mice (Apodemus sylvaticus) Carrying Non-Tuberculous Mycobacteria Able to Infect Cattle and Interfere with the Diagnosis of Bovine Tuberculosis, Microorganisms 8 (2020) 374. https://doi.org/10.3390/microorganisms8030374.

[113]	D.M. Mrochen, D. Schulz, S. Fischer, K. Jeske, H. El Gohary, D. Reil, C. Imholt, P. Trübe, J. Suchomel, E. Tricaud, J. Jacob, M. Heroldová, B.M. Bröker, B. Strommenger, B. Walther, R.G. Ulrich, S. Holtfreter, Wild rodents and shrews are natural hosts of *Staphylococcus aureus*, Int. J. Med. Microbiol. 308 (2018) 590–597. https://doi.org/10.1016/j.ijmm.2017.09.014.

[114]	P. Gómez, D. González-Barrio, D. Benito, J.T. García, J. Viñuela, M. Zarazaga, F. Ruiz-Fons, C. Torres, Detection of methicillin-resistant Staphylococcus aureus (MRSA) carrying the mecC gene in wild small mammals in Spain, J. Antimicrob. Chemother. 69 (2014) 2061–2064. https://doi.org/10.1093/jac/dku100.

[115]	S. Joutsen, R. Laukkanen-Ninios, H. Henttonen, J. Niemimaa, L. Voutilainen, E.R. Kallio, H. Helle, H. Korkeala, M. Fredriksson-Ahomaa, Yersinia spp. in Wild Rodents and Shrews in Finland, Vector-Borne Zoonotic Dis. 17 (2017) 303–311. https://doi.org/10.1089/vbz.2016.2025.

[116]	A. Platt-Samoraj, J. Żmudzki, J. Pajdak-Czaus, A. Szczerba-Turek, A. Bancerz-Kisiel, Z. Procajło, S. Łabuć, W. Szweda, The Prevalence of *Yersinia enterocolitica* and *Yersinia pseudotuberculosis* in Small Wild Rodents in Poland, Vector-Borne Zoonotic Dis. 20 (2020) 586–592. https://doi.org/10.1089/vbz.2019.2586.

[117]	A. Platt-Samoraj, K. Kończyk-Kmiecik, T. Bakuła, Occurrence and Genetic Correlations of Yersinia spp. Isolated from Commensal Rodents in Northeastern Poland, Pathogens 10 (2021) 1247. https://doi.org/10.3390/pathogens10101247.

[118]	I. Evstafiev, N. Tovpinets, Small mammals as reservoirs and vectors of yersiniosis pathogens (Yersinia enterocolitica and Y. pseudotuberculosis), Theriol Ukr. 19 (2020) 115–121.

[119]	K. Arden, K. Gedye, O. Angelin-Bonnet, E. Murphy, D. Antic, Yersinia enterocolitica in wild and peridomestic rodents within Great Britain, a prevalence study, Zoonoses Public Health 69 (2022) 537–549. https://doi.org/10.1111/zph.12943.

[120]	T.A. Giles, A.D. Greenwood, K. Tsangaras, T.C. Giles, P.A. Barrow, D. Hannant, A.-B. Abu-Median, L. Yon, Detection of a Yersinia pestis gene homologue in rodent samples, PeerJ 4 (2016) e2216. https://doi.org/10.7717/peerj.2216.

[121]	H. Zelena, P. Strakova, M. Heroldova, J. Mrazek, T. Kastl, A. Zakovska, D. Ruzek, J. Smetana, I. Rudolf, Molecular Epidemiology of Hantaviruses in the Czech Republic, Emerg Infect Dis 25 (2019) 2133–2135. https://doi.org/10.3201/eid2511.190449.

[122]	F. Sauvage, C. Penalba, P. Vuillaume, F. Boue, D. Coudrier, D. Pontier, M. Artois, Puumala hantavirus Infection in Humans and in the Reservoir Host, Ardennes Region, France, Emerg. Infect. Dis. 8 (2002) 1509–1511. https://doi.org/10.3201/eid0812.010518.

[123]	A. Papa, K. Nemirov, H. Henttonen, J. Niemimaa, A. Antoniadis, A. Vaheri, A. Plyusnin, O. Vapalahti, Isolation of Dobrava Virus from Apodemus flavicollis in Greece, J. Clin. Microbiol. 39 (2001) 2291–2293. https://doi.org/10.1128/jcm.39.6.2291-2293.2001.

[124]	A. Wójcik-Fatla, V. Zajac, J.P. Knap, J. Sroka, E. Cisak, A. Sawczyn, J. Dutkiewicz, A small-scale survey of hantavirus in mammals from eastern Poland, Ann. Agric. Environ. Med. 20 (2013). http://agro.icm.edu.pl/agro/element/bwmeta1.element.agro-082b272a-fdf6-4a80-8c5e-cfd842d8de00 (accessed June 12, 2025).

[125]	V. Nikolić, N. Stajković, G. Stamenković, R. Cekanac, P. Marušić, N. Jovanović, M. Krstić, J. Mladenović, M. Šiljić, A. Gligić, others, Comparative phylogenetic analysis of Dobrava-Belgrade virus L and S genetic segments isolated from an animal reservoir in Serbia, Arch. Biol. Sci. 66 (2014) 497–506.

[126]	B. Klempa, H.A. Schmidt, R. Ulrich, S. Kaluz, M. Labuda, H. Meisel, B. Hjelle, D.H. Krüger, Genetic Interaction between Distinct Dobrava Hantavirus Subtypes in Apodemus agrarius and A. flavicollis in Nature, J. Virol. 77 (2003) 804–809. https://doi.org/10.1128/jvi.77.1.804-809.2003.

[127]	J. Raghwani, C.L. Faust, S. François, D. Nguyen, K. Marsh, A. Raulo, S.C. Hill, K.V. Parag, P. Simmonds, S.C.L. Knowles, O.G. Pybus, Seasonal dynamics of the wild rodent faecal virome, Mol. Ecol. 32 (2023) 4763–4776. https://doi.org/10.1111/mec.16778.

[128]	S. Hazel, M. Bennett, J. Chantrey, K. Bown, R. Cavanagh, T. Jones, D. Baxby, M. Begon, A longitudinal study of an endemic disease in its wildlife reservoir: cowpox and wild rodents, Epidemiol Infect 124 (2000) 551–562. https://doi.org/10.1017/s0950268899003799.

[129]	T. Farkas, B. Fey, G. Keller, V. Martella, L. Egyed, Molecular detection of novel astroviruses in wild and laboratory mice, Virus Genes 45 (2012) 518–525. https://doi.org/10.1007/s11262-012-0803-0.

[130]	V. Bourret, L. Dutra, H. Alburkat, S. Mäki, E. Lintunen, M. Wasniewski, R. Kant, M. Grzybek, V. Venkat, H. Asad, J. Pradel, M. Bouilloud, H. Leirs, V.C. Colombo, V. Sluydts, P. Stuart, A. McManus, J.A. Eccard, J. Firozpoor, C. Imholt, J. Nowicka, A. Goll, N. Ranc, G. Castel, N. Charbonnel, T. Sironen, Serologic Surveillance for SARS-CoV-2 Infection among Wild Rodents, Europe, Emerg. Infect. Dis. 28 (2022). https://doi.org/10.3201/eid2812.221235.

[131]	E. Monchatre-Leroy, F. Boué, J.-M. Boucher, C. Renault, F. Moutou, M. Ar Gouilh, G. Umhang, Identification of Alpha and Beta Coronavirus in Wildlife Species in France: Bats, Rodents, Rabbits, and Hedgehogs, Viruses 9 (2017) 364. https://doi.org/10.3390/v9120364.

[132]	O. Mínguez-González, C.-B. Gutiérrez-Martín, M. del C. Martínez-Nistal, M. del R. Esquivel-García, J.-I. Gómez-Campillo, J.-Á. Collazos-Martínez, L.-M. Fernández-Calle, C. Ruiz-Sopeña, S. Tamames-Gómez, S. Martínez-Martínez, C. Caminero-Saldaña, M. Hernández, D. Rodríguez-Lázaro, E.-F. Rodríguez-Ferri, Tularemia Outbreaks in Spain from 2007 to 2020 in Humans and Domestic and Wild Animals, Pathogens 10 (2021) 892. https://doi.org/10.3390/pathogens10070892.

[133]	G. Hestvik, H. Uhlhorn, R. Mattsson, E. Westergren, F. Södersten, S. Åkerström, D. Gavier-Widén, Pathology of natural Francisella tularensis subsp. holarctica infection in two yellow-necked mice (Apodemus flavicollis), Acta Vet. Scand. 60 (2018) 26. https://doi.org/10.1186/s13028-018-0381-9.

[134]	T. Matějková, P. Hájková, R. Stopková, M. Stanko, J.-F. Martin, J. Kreisinger, P. Stopka, Oral and vaginal microbiota in selected field mice of the genus Apodemus: a wild population study, Sci. Rep. 10 (2020) 13246. https://doi.org/10.1038/s41598-020-70249-x.

[135]	I. Literak, M. Dolejska, J. Rybarikova, A. Cizek, P. Strejckova, M. Vyskocilova, M. Friedman, J. Klimes, Highly Variable Patterns of Antimicrobial Resistance in Commensal Escherichia coli Isolates from Pigs, Sympatric Rodents, and Flies, Microb. Drug Resist. 15 (2009) 229–237. https://doi.org/10.1089/mdr.2009.0913.

[136]	G. Zanardi, T. Iemmi, C. Spadini, S. Taddei, S. Cavirani, C.S. Cabassi, Wild Micromammals as Bioindicators of Antibiotic Resistance in Ecopathology in Northern Italy, Anim. Open Access J. MDPI 10 (2020) E1184. https://doi.org/10.3390/ani10071184.

[137]	M. Skarżyńska, M. Zając, E. Kamińska, A. Bomba, J. Żmudzki, A. Jabłoński, D. Wasyl, Salmonella and Antimicrobial Resistance in Wild Rodents—True or False Threat?, Pathogens 9 (2020) 771. https://doi.org/10.3390/pathogens9090771.

[138]	R. Cavanagh, M. Begon, M. Bennett, T. Ergon, I.M. Graham, P.E.W. de Haas, C.A. Hart, M. Koedam, K. Kremer, X. Lambin, P. Roholl, D. van Soolingen, Mycobacterium microti Infection (Vole Tuberculosis) in Wild Rodent Populations, J. Clin. Microbiol. 40 (2002) 3281–3285. https://doi.org/10.1128/jcm.40.9.3281-3285.2002.

[139]	G. Yebra, D. Mrochen, S. Fischer, F. Pfaff, R.G. Ulrich, K. Pritchett-Corning, S. Holtfreter, J.R. Fitzgerald, Bacteriophage-driven emergence and expansion of Staphylococcus aureus in rodent populations, PLOS Pathog. 20 (2024) e1012378. https://doi.org/10.1371/journal.ppat.1012378.

[140]	V. Kmeť, A. Čuvalová, M. Stanko, Small mammals as sentinels of antimicrobial-resistant staphylococci, Folia Microbiol. (Praha) 63 (2018) 665–668. https://doi.org/10.1007/s12223-018-0594-3.
